# Findings from the Kids in Communities Study (KiCS): A mixed methods study examining community-level influences on early childhood development

**DOI:** 10.1371/journal.pone.0256431

**Published:** 2021-09-01

**Authors:** Sharon Goldfeld, Karen Villanueva, Robert Tanton, Ilan Katz, Sally Brinkman, Billie Giles-Corti, Geoffrey Woolcock

**Affiliations:** 1 Centre for Community Child Health, The Royal Children’s Hospital Melbourne, Melbourne, Victoria, Australia; 2 Policy, Equity and Translation, Murdoch Children’s Research Institute, Melbourne, Victoria, Australia; 3 Department of Paediatrics, The University of Melbourne, Melbourne, Victoria, Australia; 4 Centre for Urban Research, RMIT University, Melbourne, Victoria, Australia; 5 National Centre for Social and Economic Modelling, University of Canberra, Canberra, Australian Capital Territory, Australia; 6 Social Policy Research Centre, The University of New South Wales, Sydney, New South Wales, Australia; 7 Fraser Mustard Centre, Telethon Kids Institute, The University of Western Australia, Perth, Western Australia, Australia; 8 School of Public Health, Faculty of Health Sciences and Medicine, The University of Adelaide, South Australia, Australia; 9 Institute for Resilient Regions, The University of Southern Queensland, Brisbane, Queensland, Australia; The University of the South Pacific, FIJI

## Abstract

There is increasing international interest in place-based approaches to improve early childhood development (ECD) outcomes. The available data and evidence are limited and precludes well informed policy and practice change. Developing the evidence-base for community-level effects on ECD is one way to facilitate more informed and targeted community action. This paper presents overall final findings from the Kids in Communities Study (KiCS), an Australian mixed methods investigation into community-level effects on ECD in five domains of influence–physical, social, governance, service, and sociodemographic. Twenty five local communities (suburbs) across Australia were selected based on ‘diagonality type’ i.e. whether they performed better (off-diagonal positive), worse (off-diagonal negative), or ‘as expected’ (on-diagonal) on the Australian Early Development Census (AEDC) relative to their socioeconomic profile. The approach was designed to determine replicable and modifiable factors that were separate to socioeconomic status. Between 2015–2017, stakeholder interviews (n = 146), parent and service provider focus groups (n = 51), and existing socio-economic and early childhood education and care administrative data were collected. Qualitative and quantitative data analyses were undertaken to understand differences between 14 paired disadvantaged local communities (i.e. on versus off-diagonal). Further analysis of qualitative data elicited important factors for all 25 local communities. From this, we developed a draft set of ‘Foundational Community Factors’ (FCFs); these are the factors that lay the foundations of a good community for young children.

## Introduction

Healthy early child development (ECD) is the foundation for human capital and the basis for future community and economic development [1]. A large and growing body of research emphasises the importance of the prenatal and early years (0–8 years) for health and developmental outcomes throughout the life course [2]. For a growing number of children, sub-optimal developmental trajectories are well established by the time they start school, and become increasingly difficult and costly to modify with the passage of time [3].

The ecological theory asserts that children’s development is impacted by interactions between the developing child and the proximal and distal contexts or environments in which the child is developing [4]. The environments in which children are exposed (e.g. their family, the community or neighbourhood in which they live, and local, state and federal government policies) influence their health and development; commonly referred to as the ecology of childhood [5]. Aside from genetic and biological influences, previous research has focused on more proximal family determinants of ECD [6] such as socio-demographics of the family, parental mental health, exposure to family violence and parenting styles [7]. Yet there is increasing interest and research into the effects of the neighbourhood or community environments on early childhood health and development [8–10]. ‘Community’ may refer to a place or group of people with something in common, whereas ‘neighbourhood’ tends to refer to geographic constructs or boundaries [11]. In the Australian context ‘neighbourhood’ and ‘community’ are often used interchangeably [11]. Thus in this paper, both neighbourhood and community are used.

The research into neighbourhood or community effects on the early years of development shows increasingly strong evidence that communities have an impact on children’s development. There is a social gradient in the way community level factors impact children’s developmental trajectories; the more disadvantaged the community in which a child grows up, the worse their developmental trajectory [12]. This finding is evident in Australian national data. For example, the Australian Early Development Census (AEDC), a national population measure and census of early childhood development captures information on five domains of development: physical, social competence, emotional maturity, language and cognitive skills and communication for children their first year of full-time schooling [3]. The AEDC was adapted from the Canadian Early Development Instrument (EDI), and implemented across Australia every three years from 2009 to 2018 by the Australian Government [13]. In 2018, data on 96.4% (n = 308,953) of Australian children (mean age 5 years, 7 months) were collected; 21.7% were developmentally vulnerable (i.e. children falling below the 10^th^ percentile) on at least one of the five AEDC developmental domains [14]. Furthermore, children in the most disadvantaged communities were twice as likely to be developmentally vulnerable, relative to children in the least disadvantaged areas [15]. The trend is supported by recent US evidence showing that children’s outcomes vary across census-tract neighbourhoods (average 4,250 people per area); children who live in poorer areas tend to have worse outcomes [16].

Previous research suggests that the impacts of neighbourhood disadvantage can be mitigated by community-level factors (e.g. social capital, neighbourhood facilities) that affect the functioning of families and children, particularly on the resources families can access for promoting good development [17, 18]. These converge and align with the social determinants of health—the daily living conditions in which children are born, grow, and develop [19]—focusing on the interactions between children, families and the wider social and physical environments [5]. For example, community attributes or features such as housing quality, learning environments of day cares and schools, play spaces, access to community infrastructure (physical and service environments), neighbourhood safety, social networks (social environment), and local policies (governance environment) are examples of some of the most important social determinants of child development [5, 9, 20].

Alongside the research, there is also growing *policy* recognition that communities are important environments for early childhood, as evidenced by the increasing interest in place-based approaches to child health and development. Current Australian and global ‘child-friendly city’ agendas and place-based initiatives seek to promote and protect child wellbeing through healthy communities. Globally, some ongoing examples include ‘Child Friendly Cities’ initiatives [21] launched in 1996 by UNICEF, New Deal for Communities established in 1998 in the UK [22] and the 2011 Collective Impact Initiative in the US [23]. Some examples of Australian place-based efforts include Communities for Children [24], Opportunity Child [25], Logan Together [26], and Stronger Places, Stronger People [27]. While there are context differences, what these place-based initiatives have in common is that they advocate the need for healthy communities for families and children and to tailor interventions to the local population. Despite socio-ecological frameworks of early childhood [28], there is still limited understanding of the community-level factors likely to benefit outcomes for young children and little empirical evidence of the specific modifiable community factors that may be translated into policy and action for healthier early childhood outcomes [9].

To address the need for community-level evidence for ECD, The Kids in Communities Study (KiCS) was established to examine what community attributes, other than SES, might make a difference to ECD [29]. Most importantly, the study aimed to determine which community-level factors consistently influence children’s developmental and health outcomes. To investigate this aim, the KiCS project developed a conceptual framework encompassing five domain areas (and corresponding subdomains) derived from previous research—**physical environment, social environment, socio-economic factors, access to services, and governance** [30]. Using a mixed methods approach, community-level factors posited to influence children’s health and developmental outcomes were investigated in 25 areas of high and low disadvantage across Australia. As such, we determined a draft set of foundational community factors for ECD that can be further understood in communities around Australia. This paper presents our overall results for KiCS, including the foundational community factors for ECD (FCFS); the factors that lay the foundations of a good community for young children.

## Materials and methods

The detailed study design is previously published [29], but briefly described here. In this cross-sectional mixed methods study there were three sequential phases: (1) Selection of communities (2013–14); (2) Data collection (2015–17); and (3) Data analysis (2017–18). Qualitative and quantitative data were collected to provide a better understanding of the complex and dynamic nature of the community context [31]. The qualitative research in this study investigated factors that were difficult to previously capture because of limited data availability. From qualitative and quantitative findings, we developed a draft set of FCFs, the factors that lay the foundations for creating better communities for ECD [32].

### Phase 1: Selection of KiCS local communities

KiCS capitalised on the unique availability of the Australian Early Development Census (AEDC), a population measure of ECD completed by teachers every three years for all students in their first year of school [13]. These data provide a snapshot of ECD at the local community level (suburb) [3]. Our definition of ‘local community’ thus aligns with AEDC nomenclature and geographic boundaries, the size of which varies, but in metropolitan and large regional areas, equates to approximately 10,000 persons per area on average [33, 34]. AEDC local communities are clustered within larger AEDC “communities” or local government areas (municipalities).

Selection of local communities was based on local community disadvantage using a quintile-quintile matrix of the Australian Bureau of Statistics Socio-Economic Index for Areas–Index of Relative Advantage and Disadvantage (SEIFA-IRSD) [35] and the AEDC. A local community (suburb) “diagonality type” was created i.e. those performing better or worse (“off-diagonal”), or as expected (“on-diagonal”) on the AEDC relative to area-level disadvantage. Further details are found here [33]. Through our “off diagonal” approach, there were 25 local communities clustered within 11 larger municipalities in Victoria (VIC), New South Wales (NSW), Queensland (QLD), South Australia (SA), and the Australian Capital Territory (ACT). The key objective of KiCS was to compare off diagonal communities with similar on diagonal communities in the same local government area in order to identify community factors other than SES which support early childhood development.

### Phase 2: Data collection

Between 2015 and 2017, a mix of qualitative (semi-structured interviews with stakeholders, focus groups with parents and service providers) and quantitative (community surveys, geographic information systems (GIS) software, existing demographic data, community and service surveys) methods was used to examine community factors in the each of the 25 local communities for the five community domains hypothesised to influence ECD; governance, physical, social, service, and socio-economic environments. Each community domain had a number of community factors clustered within sub-domains (21 in total; e.g. physical environment (domain): parks (subdomain); park access (factor)) as published in the KiCS study protocol [36].

Measures for each community factor were derived from a combination of the existing literature and expert advice.

The KiCS framework was derived from a synthesis of neighbourhood effects literature; more information about the framework’s domains and subdomains is published elsewhere [2, 29]. Briefly, the physical domain focuses primarily on the built form (e.g. houses, streets, local land uses e.g. parks, schools, food outlets, and access to public transport) and its social effects. The physical domain also includes environment stressors such as noise, lighting, pollution, and crowding. The social domain includes social interaction, norms and collective efficacy as well as crime, trust and safety. The socio-economic domain focuses on demographics of the community (e.g. ethnicity, education, affluence, poverty). The service domain encompasses quantity, quality, access and coordination of health care services (e.g. general practititioners, maternal and child health) and services focused on family and child wellbeing (early childhood education and care services, playgroups). The governance domain includes citizen engagement, leadership and policies and programs for the early years.

### Qualitative data

#### Semi-structured interviews

Semi-structured interviews (35 mins-1.5 hours) were conducted with key early childhood stakeholders in the local community or municipality (e.g. managers of early years’ services, local government and non-government staff involved in the early years, and school principals). Stakeholders were recruited through purposive and snowball sampling [37]. We aimed to interview approximately 8–15 participants per cluster of local communities (i.e. AEDC ‘community’ or local government area). The interview questions primarily focused on the governance and service domains, but perspectives on positive and negative (challenges or difficulties) community factors for young children and families were also asked (**S1 Appendix**). Interviews were conducted by female researchers from each state and territory (LD, AJ, JL, AS, AW, GS; RR and KV also conducted some interviews). Interviewer credentials included a mix of PhD, Master, and undergraduate degrees. LD and AJ had prior expertise and experience in qualitative methods, and assisted with mentoring the team.

Training was conducted during a face-to-face workshop, online support, email support, and fortnightly meetings between the research team. Qualitative research methods and queries were overseen by IK. To further assist with reliability, an active log was kept to document coding queries and subsequent decisions made by the team.

#### Focus groups

Focus groups were also conducted by the same female researchers who conducted the interviews. Focus groups were conducted with local service providers and parents of children aged 0–8 years living in the local community (45–90 minutes). Parents/carers also had to live in and/or use services and facilities within the local community. There were no further exclusion criteria. Focus groups had open-ended questions about each community domain (**S2 Appendix**). Service providers and parents were recruited through purposive and snowball sampling [37]. Parents were also recruited by distributing flyers through local organisations and reimbursed with an AUD $25 shopping gift card for their time and participation. We aimed to have at least 4 participants in each focus group.

For both focus groups and interviews, participants provided written and verbal consent to participate and have the interview recorded, transcribed and analysed. Participants indicated in their own words what they felt were the community factors that support or hinder children’s development in their community. Best efforts to ‘control’ for perceptions of the pre-defined local community geographic boundary were implemented (a map of the boundary was shown to participants). No further interviews or focus groups were conducted when data saturation (i.e. no ‘new’ information obtained) was reached.

#### Policy documents

While the interviews and focus group questions mainly focused on the five community domains, policy documents relating to the early years were collected to contextualise the local communities, particularly with regard to local governance. Examples of relevant policy documents were local government documents such as municipal early years’ plans, annual reports focused on early childhood and infrastructure reports. As governance structures are likely to exist across a municipality, approximately 10–12 policy documents were collected per municipality or cluster of local communities. Only those that had a reference to local communities of interest were explored.

### Quantitative data

#### Community survey

Between March and June 2016, a survey about perceptions and attitudes about the community was distributed to 1000 residents per local community (i.e. 25,000 in total), through random sampling of residents (aged 18 years or older) registered in the Australian Electoral Commission (AEC) database. Survey questions included a combination of validated items from existing surveys and derived items where existing items were non-existent [29]. Prior to the main data collection, the survey underwent test-retest reliability (two weeks apart) with a small convenience sample, to ensure face validity (content and structure). The main survey was distributed online, phone and/or hardcopy in multiple waves (pre-notification, survey, and reminder), a method adapted from Dillman [38]. Participants were offered a chance to win one of three AUD $300 supermarket gift vouchers. A sample size of at least 350–390 surveys per local community was required at 95% confidence level, and 0.05 confidence interval (i.e. a 35–39% response rate). The community survey included questions related to all five community domains of the KiCS framework, although more questions were specific to the social and physical environment domains. Survey questions included involvement in groups and activities, use and availability of local places and services, perceived traffic safety, crime safety, neighbourhood surroundings (e.g. greenery and natural sights, litter, graffti), neighbours and other people in the neighbourhood (e.g. friendships, social interaction, people out and about, trust) (**S3 Appendix**).

#### Service data and surveys

The service data included quantity, quality, access, and coordination for services related to children and families. Information such as staff capacity, clientele, opening hours, accreditation etc. were collected from online websites. Local service providers such as government representatives, school principals, general practitioners, and playgroup leaders were also asked to complete a service survey (to obtain data on service coordination and local networks) online or in hardcopy at a focus group in which they attended. The aim was to obtain a service survey from at least one service provider representative from each service type within the local community (e.g. general practitioner, primary school, and child care).

#### ABS Census data for demographic data

Data from the 2011 Australian Bureau of Statistics Census of Population and Housing (Australian Census) were used to extract SES measures (e.g. housing tenure, income, level of education) available via the ABS website and extracted using *TableBuilder* [39]. Data were extracted for the 25 AEDC local communities; this aligned closely with the geographic area, Statistical Area Level 2 (SA2; suburbs in many Australian capital cities but larger areas in regional cities). The data were extracted for the whole suburb, and not only for people with young children.

The 2011 Census was used because this was temporally closest to the 2012 AEDC data used. Further, the 2016 Census data were not available when this analysis was conducted.

#### GIS data

GIS software (ArcGIS v10.3.1) [40] was also used to create physical environment measures for the local community boundary (e.g. presence of, and distance to selected destinations, and walkability) using existing spatial datasets where possible (e.g. destination data from the Raising Children Network [41], the Australian Urban Research and Infrastructure Network (AURIN) [42] and local government websites). Measures included those aligned with the physical and service sub-domains (e.g. distance to child care, count of child care centres), and included measures previously found to be associated with child and adult behavioural and health outcomes (e.g. walking, cycling) [43, 44].

‘Quality’ data were collected on parks in each local community. Features of parks in each local community were audited using a validated remote desktop park auditing tool [45]. The ‘Public Open Space Desktop Auditing Tool’ (POSDAT) uses a combination of GIS software, Google Earth and Google Street view to capture park features and attributes. Park features include the presence or number of amenities (e.g. seating and benches, barbeque facilities, playgrounds), aesthetics (e.g. water features, shade along paths) and sporting activities (e.g. tennis courts, basketball courts). To create a child/family-friendly ‘park quality’ score for each park, each feature will be weighted and summed; this has previously been done with both adults [46], and adolescents [47].

### Phase 3: Data analysis

Data analysis involved two stages with two different analytical sub-samples: (1) Differentiating qualitative and quantitative factors for ECD in seven matched disadvantaged local community pairs (i.e. 14 communities); and (2) Important qualitative factors for ECD in all 25 local communities. In both stages, factors ‘within’ and ‘across’ local communities were examined. Findings from Stage 1 (Differentiating) and Stage 2 (Important) formed the basis of draft FCFs for ECD i.e. the factors that lay the foundations for creating better communities for ECD.

Some data were deemed unreliable for data analysis. For example, the community survey did not yield a representative sample (e.g. high proportion of older adults) or a high response rate (i.e. 16.2%), and thus was *not* recommended for further consideration in the findings. Similar to the community survey, service surveys were unsuccessful. While service providers participated in the focus groups, low response rates to the service surveys may mean that a more targeted approach (e.g. tailored to service type) to collecting information from parents and service providers about perceived service availability, access, quality, coordination, and use, is needed. Policy documents were mostly used to understand and describe the context, rather than undergo systematic analysis for this project. Policy documents were used to better understand, describe and contextualise details about local communities. For example, whether local communities had policies, frameworks, and community groups focused on the early years, and whether a historical focus on the early years existed, such as using ECD data for local decision making. Details are published elsewhere but are not the focus of this paper [48].

#### Qualitative data analysis

All focus groups and interviews were recorded and transcribed for analysis. Transcripts were imported and coded in QSR International’s NVivo v11 software program [49]. Content was deductively coded by seven researchers using a predetermined coding framework relating to the five community domains and sub-domains. The coding framework was developed and reviewed by the research team. Information that did not ‘fit’ within the existing codes but seemed to be important to the study was coded as ‘other useful information’. Issues with coding were consolidated through regular team discussions and shared documentation to ensure analytical rigour and consistency [50, 51].

Coded content were exported to MS Word for analysis using Iterative Categorisation techniques, which aims to systematically analyse qualitative data [52]. The purpose of the qualitative analysis was to identify strong emerging themes or factors related to ECD. A particular theme/factor was considered ‘strong’ if: (1) participants mentioned a particular factor without prompting or probing, or they indicated that a factor or theme as important; (2) different groups of participants (parents, professionals, policy makers) identified a common theme as being important; and/or (3) several participants indicated that a particular factor was important. Significant or important factors in the local community and how participants explained the difference between the on- and off-diagonal communities were investigated.

#### Quantitative data analysis

Strong factors differentiating an on- and off-diagonal local community were identified. Quantitative data were cleaned (e.g. missing data) in preparation for descriptive analysis. Descriptive analyses were undertaken using Microsoft (MS) Excel and Stata v14 [53] for each local community and any differences within and across matched community pairs were identified. There was no further scope to explore associations between quantitative data and the AEDC (for example, using regression models) due to the small number of local communities in our sample.

#### Stage 1: Differentiating foundational community factors

The analytical sample consisted of seven pairs of neighbouring on- and off-diagonal local communities *matched* on socioeconomic disadvantage (i.e. 14 disadvantaged local communities in total). This is important because a ‘true’ comparison is an off-diagonal matched with an on-diagonal, both holding the same SES. While quantitative and qualitative data were analysed concurrently, the qualitative data helped to inform which factors should be further investigated with quantitative data. Comparing on- and off-diagonal local communities involved assessing ‘differences’ between these communities based on Table 1.

**Table 1:** Assessment of difference between on- and off-diagonal local communities.

Data type		Assessment of ‘difference’ between on- and off-diagonal local communities
1	Qualitative data (e.g. focus groups, and interviews)	Strong emerging themes from the data were identified based on:
		Participant’s views: participants spontaneously mention a particular factor (i.e. without prompting or probing) or they indicate that a factor or theme is important.
		Triangulation: different groups of participants (parents, professionals, policy makers) identify a common theme as being important.
		Numbers: large numbers of participants indicate that a particular factor is important then this is an indication.
2	Australian Bureau of Statistics Census data (2011)	As the data used were from a Census, the data are accurate for small areas and there are no confidence intervals; we considered a ‘large’ change as a 10% difference between local communities.
3	Geographic Information Systems (GIS) and park audit data used to measure the built environment of the community	An absolute value for each physical environment feature was reported, thus it was not possible to conduct any meaningful statistical analyses to compare values within each matched pair. The magnitude of the ‘difference’ between on- and off-diagonal local communities within each matched pair was assessed by calculating the mean and standard deviation (SD) for each built environment measure across the 25 local communities in the overall Kids in Communities Study, and assessing whether the absolute value was less or more than one SD from the mean.

To help visualise the qualitative and quantitative findings for each of the community domains and sub-domains, a directional hypothesis or theory for each qualitative theme and quantitative factor was identified based on previous literature. For example, despite both being economically disadvantaged, there are more parks in the local community doing well, compared with the local community doing poorly. A three-staged approach was then used:

**Stage 1:***Within* community pairs: Does the theme/factor differentiate between on- and off-diagonal local communities?
**Stage 2:***Across* community pairs: Is there a consistent pattern emerging across community pairs? For both qualitative and quantitative measures, a ‘consistent pattern’ was whether the same finding appeared in at least *four or more* community pairs.
**Stage 3:***Overall triangulation* of qualitative and quantitative data: Do the qualitative and quantitative findings match? Where possible, a qualitative and (equivalent or proxy) quantitative measure were conceptually aligned (i.e. are they measuring the same construct?).

The aim of the triangulation was to provide more support for any consistent community-level factors associated with on-and off-diagonal communities. The triangulation process (or convergent validation) enabled a broader and deeper exploration of domain-specific community factors in on- and off-diagonal communities [31]. As such, integrating qualitative and quantitative data facilitates the conceptualisation of potential mechanisms with rich contextual understanding to explain complex interactions relating to community-level factors that may influence ECD.

#### Stage 2: Important foundational community factors

Stage 2 analysed only qualitative data for *all* local communities, regardless of their diagonality status. That is, are there any community-level factors that are consistently perceived as important for families and young children? Participants indicated in their own words what they felt were the factors that support children’s development in their community. Strong qualitative themes or factors were identified both within and across all 25 local communities in KiCS using the same qualitative approach described earlier except comparisons between ‘better than expected’ and ‘as expected’ local communities were not the focus. Factors considered *consistently* important for ECD were those that appeared in *at least 16* of the 25 local communities.

### Ethics

The Royal Children’s Hospital Melbourne Human Research Ethics Committee (30016) provided overall ethics approval, and further ethics or site approvals were received from other ethics committees and institutional boards: 1. Victorian Catholic Education Melbourne; 2. Victorian Catholic Education Sandhurst; 3. Victorian Department of Education and Early Childhood Development (now Victorian Department of Education and Training); 4. South Australian Department of Early Childhood and Development; 5. Children’s Health Queensland Human Research Ethics Committee; 6. Queensland Wide Bay Hospital and Health Service; 7. Queensland Cairns and Hinterland Hospital and Health Service; 8. Queensland Department of Education, Training and Employment; 9. New South Wales Catholic Education Diocese Maitland-Newcastle; 10. New South Wales Catholic Education Lake Macquarie; 11.New South Wales Catholic Schools Office Diocese of Broken Bay; 12. New South Wales Department of Education; 13. New South Wales Hunter New England Human Research Ethics Committee; 14. New South Wales Central Coast Local Health District; 15. New South Wales Illawarra Shoalhaven Local Health District; and 16. New South Wales Hunter New England Local Health District.

## Results

### Sample characteristics

Most of the 25 local communities were in urban (n = 18; 72%), and disadvantaged areas (n = 17; 68%), and 13 were considered off-diagonal (52%) (Table 2). The sub-sample of 14 on- and off-diagonal local communities matched on disadvantage SES (i.e. disadvantaged doing poorly vs. disadvantaged doing well) were spread across VIC (n = 2), NSW (n = 6), QLD (n = 4), and ACT (n = 2) and used for the differentiating factors analysis (Stage 1) while all 25 local communities were used for important factors analysis (Stage 2).

**Table 2:** Snapshot of local communities.

			Geographic region		Off-diagonal		On-diagonal	
State/Territory		Local communities	Urban	Regional	Positive	Negative	Advantaged	Disadvantaged
		n = 25	n = 18	n = 7	n = 8	n = 5	n = 3	n = 9
1	VIC	6	3	3	1	2	2	1
2	NSW	6	6	0	3	0	0	3
3	SA	4	4	0	1	1	0	2
4	QLD	6	2	4	2	1	0	3
5	ACT	3	3	0	1	1	0	1

VIC: Victoria; NSW: New South Wales; SA: South Australia; QLD: Queensland; ACT: Australian Capital Territory; Off-diagonal positive: Low socio-economic status, better than expected early childhood development outcomes; Off-diagonal negative: High socio-economic status, worse than expected early childhood development outcomes; On-diagonal advantaged: high socio-economic status, as expected early childhood development outcomes; On-diagonal disadvantaged: low socio-economic status, as expected early childhood development outcomes.

A summary of qualitative data collection (field work) is in Table 3. While KiCS aimed to conduct at least one parent and practitioner focus group per local community, at least 8 interviews per local government area (community), and obtain a 30% response rate for each local community, these ‘targets’ varied for each state and community. Despite best efforts, recruitment challenges varied by context, and ranged from ethics rejections (e.g. primary schools) to difficulties in accessing local parents and service providers. In communities where no parent focus groups were held, interviews using focus group questions were held where possible.

**Table 3:** Summary of field work in local communities.

Summary of fieldwork	Key stakeholder interviews	Focus groups with local service providers	Focus groups with parents of children aged 0–8 years
VIC-1-1 Off-diagonal positive		1 (14x participants)	2 (14x participants)
VIC-1-2 On-diagonal disadvantaged		1 (9x participants)	1 (6x participants)
VIC-1-3 On-diagonal advantaged		1 (7x participants)	1 (4x participants)
Total VIC-1	21	3	4
VIC-2-1 Off-diagonal negative		1 (10x participants)	1 (6x participants)
VIC-2-2 On-diagonal disadvantaged		1 (14x participants)	2 (22x participants)
VIC-2-3 On-diagonal advantaged		1 (6x participants)	2 (17x participants)
Total VIC-2	15	3	5
NSW-1-1 Off-diagonal positive		3 (15x participants)	2 (7x participants)
NSW-1-2 On-diagonal disadvantaged		1 (7x participants)	1 (3x participants)
Total NSW-1	10	4	3
NSW-2-1 Off-diagonal positive		3 (18x participants)	1 (9x participants)
NSW-2-2 On-diagonal disadvantaged		1 (11x participants)	1 (6x participants)
Total NSW-2	10	4	1
NSW-3-1 Off-diagonal positive		1 (5x participants)	2 (11x participants)
NSW-3-2 On-diagonal disadvantaged		1(8x participants)	2 (9x participants)
Total NSW-3	15	2	4
SA-1-1 Off-diagonal negative		1 (6x participants)	1 (7x participants)
SA1-2 On-diagonal advantaged		1 (6x participants)	1 (3x participants)
Total SA-1	14	2	2
SA-2-1 Off-diagonal positive		2 (6x participants)	1 (4x participants)
SA-2-2 On-diagonal advantaged		1 (8x participants)	1 (5x participants)
Total SA-2	14*	3	2
QLD-1-1 Off-diagonal positive		1 (5x participants)	0
QLD-1-2 On-diagonal disadvantaged		1(4x participants)	1 (5x participants)
Total QLD-1	12	2	1
QLD-2-1 Off-diagonal positive		1(7x participants)	1 (4x participants)
QLD-2-2 On-diagonal disadvantaged		1 (7x participants)	1 (3x participants)
Total QLD-2	10	2	2
QLD-3-1 Off-diagonal negative		1 (9x participants)	1 (3x participants)
QLD-3-2 On-diagonal disadvantaged		1(9x participants)	1 (6x participants)
Total QLD-3	10	2	2
ACT-1-1 Off-diagonal positive		See below	0
ACT-1-2 On-diagonal disadvantaged		See below	0
ACT-1-3 Off-diagonal negative		See below	0
Total ACT-1	9*	1 (4x participants)	2 (interviews with parents instead of focus groups)
TOTAL	146	28	26

Off-diagonal positive: Low socio-economic status, better than expected early childhood development outcomes; Off-diagonal negative: High socio-economic status, worse than expected early childhood development outcomes; On-diagonal advantaged: high socio-economic status, as expected early childhood development outcomes; On-diagonal disadvantaged: low socio-economic status, as expected early childhood development outcomes. ACT: Australian Capital Territory; QLD: Queensland; NSW: New South Wales; SA: South Australia. VIC: Victoria. Coding convention: ‘State-Community-Local community’. Note: 2x parent focus groups are pilot focus groups, 1 in VIC and 1 in NSW; 2x interviews for NSW-3 are also for NSW-1 and NSW-2;

*More than one interviewee in some SA and ACT interviews.

### Differentiating foundational community factors

There were 13 FCFs that consistently differentiated on- and off-diagonality in most disadvantaged local communities (i.e., at least four matched disadvantaged community pairs; Table 4). These FCFs were largely qualitative, with fewer FCFs with quantitative measurement. There were four FCFs with quantitative measurement; (1) income; (2) highest level of schooling; (3) housing tenure (stability); and: (4) public housing.

**Table 4:** Foundational community factors for early childhood.

**Differentiating Foundational Community Factor –**			**Summary finding**
**Factor that differentiates disadvantaged communities doing well or poorly on ECD**			**(≥4 of 7 community pairs)**
1	**Income** ^a^	Median household income^1^ and degree of economic diversity^2^ is greater in disadvantaged areas doing well on ECD	6 of 7^1^; 6 of 7^2^
2	**Highest level of schooling** ^a^	There is a higher proportion of the population that have completed Year 12 or equivalent^1^ in disadvantaged areas doing well on ECD	4 of 7^1^
3	**Gentrification** ^a^ ^,^ ^c^	Relatively higher income (but still disadvantaged) families are moving into disadvantaged areas doing well on ECD, resulting in the displacement of more disadvantaged groups^2^	5 of 7^2^
4	**Housing affordability** ^a^ ^,^ ^b^	Housing is perceived as more affordable in disadvantaged areas doing well on ECD^2^	4 of 7^2^
5	**Housing tenure (stability)** ^a^	There is a lower proportion of renters compared to private home owners in disadvantaged areas doing well^1^	6 of 7^1^
6	**Public housing** ^a^ ^,^ ^b^	There is a lower proportion of public renters^1^ and less perceived presence of public housing^2^ in disadvantaged areas doing well on ECD	5 of 7^1^; 5 of 7^2^
7	**Housing density** ^b^ *(linked to public housing)*	There is a lower proportion of high rise (three or more storeys)^1^ and perceived fewer high rise density dwellings (vs low rise housing developments)^2^ in disadvantaged areas doing well on ECD	4 of 7^1^; 5 of 7^2^
		*Public housing type*: Compared with OnDis, there are more public housing classified as separate houses in Off+ compared with public housing classified as town houses/apartments	5 of 7^1^
8	**Stigma** ^c^	Negative reputation of a local community^2^ is less in disadvantaged areas doing well on ECD^2^	5 of 7^2^
9	**Primary school reputation** ^c^	Primary school reputation was more positive in disadvantaged areas doing well on ECD^2^	6 of 7^2^
10	**Perceived ECEC availability** ^d^	There was more perceived ECEC availability in disadvantaged areas doing well on ECD^2^	4 of 7^2^
11	**Perceived crime** ^c^	There was less perceived crime in disadvantaged areas doing well on ECD^2^	5 of 7^2^
12	**Historical events** ^e^	Leaders respond to events in ways that bring local community members together to create a shared storyline and/or engage in activities of citizenship^2^ is greater in disadvantaged areas doing well	4 of 7^2^
13	**Local decision-making** ^e^	As a result of local decision-making, ‘novel approaches’ or locally tailored initiatives or solutions (including any with a focus on social capital) have been developed in the community doing well^2^	4 of 7^2^
**Important Foundational Community Factor**			**Summary finding (≥16 of 25 local communities)**
**Factors perceived as important for local communities** ^2^			
14	**Physical access to services** ^b^	Reported instances of ability to get to services	19 of 25
15	**Walkability** ^b^	Perceived walkability to facilities and services was seen as important for physical access	16 of 25
16	**Public transport availability** ^b^	Perceived presence of/access to public transport was seen as important for easy access within the suburb	20 of 25
17	**Traffic exposure** ^b^	Being away from traffic within the suburb is an important factor for children being safe	16 of 25
18	**Public open space–availability and quality** ^b^	Having parks in the suburb was seen as important for young children and families. Having good quality parks was seen as important for use, play and social interaction	20 of 25
19	**Facilities–availability and diversity** ^b^	Having a range of family-friendly destinations and activities is important for young families and children	20 of 25
20	**ECEC cost** ^c^	Perceived affordability of ECEC is considered important and affects use	17 of 25
21	**Leadership** ^e^	The presence of local champions, leaders and boundary spanners driving local governance	19 of 25

^1^Quantitative;

^2^Qualitative;

^a^SES;

^b^Physical;

^c^Social;

^d^Service;

^e^Governance

Income and public housing presence and density were the only FCFs that had overall triangulation for both qualitative and quantitative measurement. While a number of FCFs are considered cross-domain factors (i.e. they do not exclusively belong to one domain), in general, four differentiating FCFs were related to Housing (SES, physical and social domains), and two differentiating FCFs emerged from each of the service (perceived availability of early childhood education and care, primary school reputation), social (stigma, perceived crime), and governance domains (historical events and local decision-making). The socio-economic domain consisted of three differentiating FCFs. Aside from housing factors, no other definitive physical domain factors were found as differentiating on- and off-diagonal disadvantaged local communities.

### Important foundational community factors

There were eight important FCFs. While few physical domain factors differentiated diagonality, six appeared to be consistently important for local communities. The presence of local community champions (governance domain) and cost of early childhood education and care services (service domain) were also considered consistently important by communities.

## Discussion

We established a set of foundational community factors (FCFS) consisting of 13 differentiating FCFs (those associated with better ECD outcomes in disadvantaged local communities) and eight important FCFs (those found to be consistently related to ECD in qualitative data across communities). The differentiating FCFs were mostly related to socio-economic, local governance, service and social domains; noting the domains are interrelated and not mutually exclusive. In particular, socio-economic FCFs such as income, highest level of schooling, and housing factors (e.g. public housing, housing tenure, housing affordability) emerged in at least 6 of the 7 disadvantaged community pairs, and appear to be related to other differentiating FCFs (e.g. stigma, perceived primary school reputation). While we initially hypothesised that off-diagonal positive local communities would have ‘better’ physical environment conditions to facilitate child health and development than on-diagonal local communities, there were few physical domain FCFs that made a difference to diagonality. Nevertheless, many communities perceived physical environment features (e.g. parks, facilities, public transport) as important for families with young children.

Some key themes emerged from our research that are unique in terms of the current research related to child development and neighbourhoods. These focus on: (1) the potential implications of gentrification and economic diversity, (2) the impact of stigma, (3) the difficulty in measuring the impact of the physical environment, and (4) the potential disconnect between objective data and the subjective perceptions of the neighbourhoods in which families are raising their children (even within the limitations of our sample size).

Our main findings suggest it may be the combination of family and community, working in synergy, which contribute to children’s outcomes. These findings are in line with socio-ecological frameworks of child development, which posit that children grow, develop, interact, and are exposed to multiple levels of environments, including the family, school, and community environments [28]. While the KiCS framework provides a way to capture and organise community level influences, the emergent foundational community factors were not seen as being exclusive from each other, rather they may interact with each other (and with families and individuals) along the pathway to influence child development. Based on our data with support from previous literature, we attempted to explain some of the ways in which these factors act as determinants in children’s development.

### Income diversity, housing and gentrification

Income diversity remained a consistent factor which differentiated those disadvantaged communities with better ECD outcomes compared to their neighbouring disadvantaged communities where outcomes were relatively poor. These findings were supported by both the qualitative and quantitative measures. We found that in disadvantaged local communities, economic diversity (a mix of relatively higher and lower incomes) appeared to promote ECD.

Indicators of neighbourhood socio-economic status or composition (e.g. income, education) offer the strongest evidence available for ‘neighbourhood effects’ on ECD [54]. Neighbourhood disadvantage has been linked with poorer child health and development outcomes such as educational achievement and behavioural outcomes, even when controlling for individual-level income and education [55, 56]. Despite neighbourhood disadvantage (as measured by SEIFA-IRSD (quintiles), an area level index combining income, education etc) [35] being part of our selection criteria for on- and off- diagonal local communities (i.e. disadvantaged doing as expected compared to disadvantage doing better than expected), income differences still existed between our local communities even though they were both disadvantaged (e.g. in the same quintile) suggesting even those relatively small differences (and the factors contributing to those differences) potentially influences ECD.

Previous studies have suggested that the presence or absence of affluent neighbours may influence children’s development outcomes [57]. It was also found that young children’s IQs were higher in neighbourhoods with greater concentrations of affluent neighbours, while having low-income neighbours appears to increase the incidence of externalizing behaviour problems [58].

The deconcentration of poverty involves mixing households with different incomes. Gentrification appears to be the most prevalent means of deconcentrating poverty in recent years [59], which may consequently have a role at the neighbourhood level in promoting or mitigating developmental inequalities [9]. Gentrification is a process of socio-economically selective migration that sees relatively higher income and higher consuming households move into lesser valued, ‘more affordable’ urban areas where their investment sees more “bang for the buck” [60]. As such, gentrification is not just a characteristic of the urban growth and regeneration (physical domain), it includes changes to the community’s SES profile (e.g. high incomes, university-level qualifications, and employment in professional positions) (socio-economic domain). In KiCS, disadvantaged local communities where children were doing better than expected were gentrifying more than the disadvantaged local communities where children were not doing well. Our FCFs of income, highest level of schooling, and housing (affordability, tenure (mobility or transience)) may be related to gentrification processes.

Gentrification also implies the displacement (outflow) of lower-income residents and is therefore often a concern in driving inequalities. One key element contributing to this displacement is housing (socio-economic, physical domains) [60]. Gentrification processes can be driven by national policies and flexible housing market regulations (governance domain), which may contribute to housing affordability issues [61]. As demand for housing increases, a subsequent rise in property values may contribute to poorer residents being displaced as relatively wealthier people move in. We found housing affordability was ‘less of an issue’ in local communities where children were doing better than expected compared with local communities where children were not doing well, despite being socio-economically similar. As such, levels of displacement have been a contentious issue because it may further concentrate poverty for the more disadvantaged families. However, a recent study found that poorer residents were less likely to move out of gentrifying neighbourhoods than non-gentrifying neighbourhoods [62].

### Stigma

Stigma was an interesting finding that strongly emerged from the qualitative data. Based on previous literature, we did not initially intend to measure stigma. However, we found disadvantaged local communities where children were doing better than expected had a better reputation (less stigma). Stigma can be attached to particular pockets or areas within the community e.g. schools, housing estates, or the negative reputation can be attached to the whole community. Indeed, we found public housing and primary school reputation as differentiating FCFs. It may be growing up in areas or attending schools with a negative reputation may likely affect children’s self-esteem and aspirations for the future [10, 63]. Community stigma can be perpetuated through media and through children’s indirect exposure to negative judgements of parents and other family members, or through community norms or shared values, which may sustain community stigma [18]. It results in the risk of being judged, stereotyped, and consequently, children may experience bullying.

There may be ways to ameliorate stigma associated with living in public housing. For example, our findings show that it is not the mere presence (or absence) of public housing that might differentiate why some disadvantaged local communities are doing better than others on ECD. Rather, it refers to how public housing is *distributed* across the community (e.g. located in concentrated pockets or otherwise ‘scattered’), and the housing type (e.g. higher rise density public housing vs. more separate housing). For example, participants referred to public housing as ‘not being so obvious’ or it ‘looked like any other house’ if it wasn’t higher-rise density housing types located together in the same area.

### Lack of physical environment differentiating factors

Aside from housing factors, there were no other differentiating FCFs from the physical domain. Our findings are consistent with previous literature. Associations between neighbourhood disadvantage and children’s behavioural outcomes was not significantly explained by the quality of facilities such as parks, play spaces, roads, and access to shopping, services and transport [9]. It may be that physical environment factors are more distal to ECD and play its role by interacting with other community factors along the pathway of influence [54]. Complex interactions between community factors (e.g. social and socio-economic, social and physical environment) need to be explored in future research. That is, the physical environment provides the conditions that help facilitate or hinder family lifestyle choices and behaviours, which in turn, impact on children’s health and development. Another reason for few differentiating physical domain FCFs is measurement limitations (see Limitations section). Nevertheless, while there were no differentiating factors, it was clear that many factors from the physical environment domain were perceived as consistently important for families with young children and our findings re-emphasise the need for equitable environments for families with young children. Factors perceived to be important for local communities included having local facilities and physical access (e.g. public transport, walkable environments) to services. Families with more resources (e.g. income) for example, may likely travel outside neighbourhood to access important services and resources, while families with less resources, are likely to be more susceptible to the availability and quality of services and destinations within their local area. Thus, features such as quality and availability of local services, in particular physical access (e.g. walking distance to and from places, public transport accessibility) becomes more important for the most vulnerable and disadvantaged. Such findings support that children exist in relation to interacting with other people, resources and opportunities within the community [9].

### Strengths

Investigating community-level factors associated with ECD is a relatively unexplored area of research compared with family, school and individual factors. The main strength of this study includes the large qualitative component, providing in-depth data about communities. As such, it enabled further opportunities to explore why children may have higher AEDC scores in disadvantaged local communities and elicit factors consistently important for families with young children across all communities. Guided by previous literature and our conceptual framework (five community domains) [11], we had both quantitative and qualitative data to provide a better understanding of the local context for different types of local communities, and the community factors that are important for early childhood within these communities. While our objective measures and subjective findings did not always align (triangulate), the mixed quantitative and qualitative data helped to provide a more in-depth understanding of the community factors associated with ECD outcomes in communities and has the potential to extend lines of enquiry in future research. As such, we recommended a set of evidence-informed ‘foundational community factors’. Quantitative data provides the ‘what’ and the scale of the problem, while qualitative data provides the ‘why’ and how the problem can be improved. It is this complexity that is a major strength of the study.

### Limitations

The cross-sectional design and small sample size precluded any analysis that could determine the mechanisms in which communities make a difference to ECD, and which factors may be ‘more important’ than others. Others have tried to quantify and model the relationships between community-level effects and ECD with quantitative data, and isolate which community factors exert a larger ‘difference’ on ECD [40]. Such work has a long way to go. With further research, more robust evidence on the pathways (how and why) in which community factors (what) influence ECD, and at what scale (how much) will make a difference to ECD. While the KiCS framework provided a way to organise or capture community-level factors and processes, other factors not explicitly mentioned in the framework may have emerged from the qualitative data collected. Other factors which may influence ECD includes air pollution [64], food insecurity [65], and access to online and digital technologies [66]. A revised framework could include explicit mention of these factors.

The original intention was to derive a set of initial indicators from factors that either consistently differentiated or deemed important by communities (Stage 2). While we initially intended to create robust community indicators (specific, measurable and repeatable over time [67]), we faced challenges with the complexity of different community contexts, and limitations with quantitative measurement and modelling (e.g. small number of communities). Indicators have traditionally been quantitative (rather than qualitative) and such challenges limited the number of quantitative indicators. Quantitative data (e.g. ABS Census, service information, community survey, and GIS data) were sourced for a relatively small number of local communities. This presents challenges in the representativeness of results and ability to conduct further statistical modelling [68].

The intricacies of different community contexts also limit generalisability. For example, rural communities were not examined in this study so our findings may not be generalisable to other contexts. Nevertheless, the FCFs may provide communities with a ‘checklist’ or starting point in which communities can discuss priorities and insights into which factors play a role at the local level, and how they might address them. For example, fostering a sense of community may be of higher priority in one community than having walkable streets and accessible public transport.

There are also differences within and between neighbourhoods. Disadvantaged neighbourhoods may vary in terms of risk factors (e.g. crime rates, neighbourhood safety) and protective factors (e.g. social capital, collective efficacy).

The KiCS FCFs were measured at the suburb-level and may not necessarily capture ‘pockets of dis/advantage’ within each community or particular areas or places of concern (e.g. limiting the specificity of GIS data). There may be differences between sub-groups (e.g. cultural groups) within the community and the FCFs may likely perform differently across different groups [69]. We did not interview, conduct focus groups or implement surveys with children. Further research is needed to capture: (1) differences within neighbourhoods to identify opportunities to flatten inequities; and: (2) perceptions of sub-groups within the community, including children’s perceptions of the communities in which they live.

### Implications

Despite the increasing global interest in “place-based” strategies from governments and philanthropic agencies around the world the availability of rigorous approaches to data collection and indicators, underpinned by theory and tested for associations with ECD outcomes, is limited. The development of *initial* community-level ECD indicators and foundational community factors can contribute to place based efforts.

Relationships between the community factors, families and children are complex, with no ‘magic bullet’ approach to improving outcomes for young children. Nevertheless, the FCFs provide some key focus areas for communities to consider for possible local place-based initiatives, despite the caveats and interdependencies with other FCFs. From the qualitative information in particular, we highlight that every community is different, and the FCFs can be used as a starting point for communities to discuss and prioritise what may be important for their local context. The FCFs were developed to help communities understand the community-level factors that might be associated (rather than lead to) relatively better early childhood development.

The KiCS FCFs are the result of a “deep dive” into 25 communities. The level of depth has resulted in rich qualitative data with limitations on quantitative data due to the small sample size. As such, KiCS has produced a limited set of indicators We recommend two further programs of research to: (1) test the utility and replicability of the foundational community factors in communities; and: (2) strengthen the quantitative indicators through further analyses.

## Conclusion

KiCS aimed to examine community-level effects on young children’s development. Exploring factors that may be modified ‘at scale’ (i.e. has the potential to impact all young families and children in a community) is important. While quality early life experiences are important for all children, they have been shown to be particularly vital to overcoming the effects of disadvantage [70]. The differentiating FCFs provide potential focus areas that communities can consider to improve ECD outcomes through evidence-informed place-based initiatives [71]. Measuring, monitoring and stimulating collective local effort is important for creating and maintaining the continuity of intervention needed to sustain good ECD outcomes and create more equitable communities for children [72].

## Supporting information

S1 Appendix Interview guide. (PDF)

S2 Appendix Focus group guide. (PDF)

S3 Appendix Community survey. (PDF)

S1 File (PDF)
