# Supplementary material for: Findings from the Kids in Communities Study (KiCS): A mixed methods study examining community-level influences on early childhood development
Source: PLoS One. 2021 Sep 1;16(9):e0256431. doi: 10.1371/journal.pone.0256431 (PMC8409665; doi:10.1371/journal.pone.0256431)
Supplement: S1 Appendix — (PDF) [file pone.0256431.s001.pdf]

S1 Appendix: Interview guide

The data collection for the Kids in Communities Study (KiCS) project includes semi-structured interviews. The aims of the semi-structured interviews are:

- to consider how communities involve citizens, make decisions, solve problems, mobilise resources and create change at the local level (governance domain)
- collect qualitative information about the quality, quantity, access and coordination of service provision in the communities from the perspective of decision makers (service domain)

The semi-structured interviews are designed to collect information from decision makers and other individuals involved in the local community who are not involved in direct service delivery to families and children. This would include (where relevant):

- The Mayor/local councillors, other local leaders including any early childhood champions
- Council staff (local government) responsible for the early years
- Maternal and child health coordinator / managers of other early childhood education and care services, school principals, playgroup coordinators
- Partnership, leadership or governance groups established around early childhood
- CEOs, Managers and key staff of local NGOs, welfare services (eg church), local associations, community centres, hub schools, outreach service providers
- Chairs/members of local committees including early childhood (i.e. playgroups and childcare); school councils
- Migrant or refugee advocates (where applicable or relevant)
- May also include members of advocacy or residents groups, sport clubs, traders associations, local businesses
- May include local political parties, local Members of Parliament (state and territory) where relevant (i.e. where they have a portfolio or expressed personal interest in ECD – this should be considered in the desktop analysis).

The interviews do not target front line child and family workers, these stakeholders should be involved in focus groups rather than interviews.

The aim is to undertake 10-15 interviews per KiCS on and off diagonal constellation. There may be some communities where fewer interviews are required because there are few individuals involved in governance and services.

Researchers need to guide the interviews carefully as the KiCS on and off communities do not necessarily conform to geographic boundaries that make sense to the local communities. Researchers will need to ask individuals to specifically think about families with young children (0-8) and the universal health, care and development services for families with young children. Researchers should have the *KiCS local communities boundaries map*(s) to hand and these may also be emailed in advance. When conducting the interview, focus attention on the areas in the map, asking the interviewee to focus on each of the suburbs or areas in the constellation (where relevant) and also on the local government area more generally.

The interview style is semi-structured, this means that the questions have been prepared to be followed but open ended questions and prompts enable the researcher to follow 'topical trajectories', there is a nice short article on semi-structured interviewing here: http://www.qualres.org/HomeSemi-3629.html

Introductory Script

Before starting, refer to the *interview data collection checklist* and the protocols outlined in the document *KiCS language when talking to communities*. It will be necessary to have the KiCS local community boundaries map(s) with you for reference.

The Kids in Communities study (KiCS) was established in 2014 to better understand how different factors in communities influence the way children develop. We want to talk with you about the community/ies shown

in these maps. These are some of communities from 5 states and territories that have been selected to help better understand and capture the factors about where we live or work that predict healthier child development. In doing this we have selected a variety of measures to see how they vary between communities.

Your contribution to this data collection is really important to us. We want to know more about this community/ies and how things work here. There are no right or wrong answers – we would like to know your thoughts.

When thinking about the community, we hope you think of early childhood as the age group 0-8 and focus on areas you are familiar with, that are shown in the maps I have brought along and/or the local government area more generally. If you are unsure about anything in a question I ask, please let me know.

**Part A**<br>**About you**<br>Let's get started by finding out a bit more about you.	1. Can you please state your name, role in the community/ies, role title and organisation?<br>2. Can you tell me a little more about the geographical area you are involved with in this role?<br>3. Do you or your organisation represent or provide services to families or children? Can you tell me a bit more about what you do?
**Part B**<br>**About the community/ies**<br>As part of the KiCS study, we want to focus on suburb boundaries defined by the AEDC. We are interested to know more about whether these boundaries make sense to you and to your role. (using the map)<br>We are going to focus on these areas but if something comes to mind that you think is important outside these areas we would like to hear more about it.	*4.* Do you think the area(s) shown in the map and the name(s) of the communities make sense to people who live or work here?<br>*What do people who live or work in these area(s) call them? Are there other names for the community/ies or other ways people define the geographical boundaries of this community/ies?*<br>5. Can you identify which areas on the map(s) you are involved with in your professional and/or personal role?<br>6. How long have you had this role and/or been involved with the community around here?<br>*7.* Thinking about (community shown on the map), can you briefly tell me what you know about this community?<br>*What do you know about the history of the area? What sorts of people live here (ie young families, retired, migrants etc), what is the local economy like, what sorts of jobs do people have, do people work in the community or commute, is the population cohesive; do people come and go or do they stay? Why do they stay/leave?*<br>*8.* Thinking about (community), in your experience, what are the good things about this community for families and children?<br>*Is it good environment for families with young children, are there services and parks, do people find it easy to get around?*<br>*9.* Thinking about (community), in your experience what is not so good, do families face challenges or difficulties?<br>*Are there reasons why families experience difficulties?*<br>*10.* Thinking about (community), in your experience what are the services for families with children like in this area<br>*Are the services of high quality, is it possible to access services, are there long wait lists, are the times provided convenient and affordable?*<br>(Logic: Repeat 7, 8, 9 for each relevant community)

**Part C. Representation and leadership**<br><br>In this part of the interview, I want to find out more about which individuals and organisations are important in this community when it comes to services and policy for families and children.	*11.* In your experience, what or who are the important organisations or individuals in influencing decisions about early childhood (0-8years) services or policy in this community?<br>*Who do they represent? What role do they play?*<br>12. In your experience, how do decisions about early childhood policy and services get made in the community?<br>o *How much say do local people have about what happens in this community? Under what circumstances do people get to have a say?*<br>o *How much say do local organisations have about what happens in this community?*<br>o *What data or information is used for decision-making?*<br>13. Can you identify any particular individuals that have had a big influence on early childhood policy or services in this community? Who and what was this influence?<br>14. Do you think local people can easily get involved in early childhood policy and service improvement or provision in this community? Do they get involved?<br>o *How can citizens get involved (eg consultations, surveys, public meetings?)? How is participation supported? Do you think some groups are excluded? Do you think people would like to be more involved?*<br>15. Do the people you represent or provide services to get to have a say about services or policy? *If yes, can you elaborate?*<br>16. Would you say you have a specific role or knowledge about early childhood policy, services and programs in this community?<br>Logic: If yes, continue to Part D, if no, skip to Part F.
**Part D Macro/Meso environment**<br><br>In this part of the interview, I want to find out more about the role that you or your organisation has in development of services or policies.	17. Can you briefly describe your (organisational/individual) relationship with your local government? State government? Federal government?<br>18. Is the role of federal and state government important to the local policy context for early childhood here?<br>19. What is the current policy agenda or priorities for early childhood service provision? Are there particular grants for child and family services in these communities at present, can you describe them?<br>20. In your experience, do state and federal government personnel from different departments/portfolios cooperate in this area?<br>21. Are there local leaders, policy entrepreneurs, organisations that influence policy at the state / federal level (or influence state/federal policy implementation locally)<br>22. What do you know about the history/legacy of early childhood policy/services in this area? What existed ten years ago? Have things changes or have priorities shifted in the past ten years or so?<br>23. Do you know if local early childhood partnerships / or early childhood plans are required by local/state/federal government in this area? If yes, can you briefly describe them?<br>*24.* Are you or your organisation part of a network or partnership with a role in local early childhood services or policy? *If yes, does this influence extend*

	beyond the local government area to a regional, state or federal level? Can you tell me a bit more?<br><br>If yes to 23 (and you have time), go to part E, If no to 22, go to part F. If no time, think about making another appointment?
**Part E**<br>**Early years partnership**<br><br>You mentioned previously that you or your organisation are involved in a network or partnership with a focus on early childhood policy or services. I would like to find out more about that network or partnership – do you have time to talk about it now?	25. What is the name of the early years network or partnership?<br>26. Is the partnership formal / informal? How is it organised or structured? When does the partnership meet and how often? Is there a convenor or a champion?<br>27. How and why was the partnership initiated? Does it receive any funding or in-kind support?<br>28. Do partners have a common goal or vision? Are the roles and responsibilities of the partners clearly defined?<br>29. Who are the members, are there any state or federal government members?<br>30. Who attends meetings, CEOs, decision-makers? Or grass roots community staff? Is the partnership able to authorise resource allocation or change?<br>31. What data and evidence is used to guide decision-making. To what extent does the group use AEDC results and other ECD research?<br>32. Would you say that the professional relationships between partnership members are trusting? Conflictual? Consensual? Is there an effective process for solving conflict if it arises?<br>33. Does the partnership measure whether it has effective planning processes? Does the partnership have processes for measuring the impact of its role? How does the partnership measure, celebrate and embed success?
**Part F**<br>**Concluding points**<br><br>We have nearly completed the interview, I just wanted to check if you had any other information or contacts you think might be valuable to the study.	34. Thinking about our discussion, do you think there is anyone else I should talk to about early childhood policy or services in this community?<br>35. Are there any other points you would like to raise about children and families in this community/ies?<br>36. Are there other questions you think I should ask local people and organisations about leadership, policy or child and family services in this community?<br><br>That concludes the interview. Thank you for your time, we will be keeping in touch with participants. A summary of results will be prepared and you will get a copy.
