# Supplementary material for: Findings from the Kids in Communities Study (KiCS): A mixed methods study examining community-level influences on early childhood development
Source: PLoS One. 2021 Sep 1;16(9):e0256431. doi: 10.1371/journal.pone.0256431 (PMC8409665; doi:10.1371/journal.pone.0256431)
Supplement: S2 Appendix — (PDF) [file pone.0256431.s002.pdf]

S2 Appendix: Focus group guide

This is the focus group guide for the Kids in Communities Study.

1 Introduction

As you know, I'm from the [insert university name] and we are talking to people in [name of community] to find out what you think about the area you [live/work] in. In particular we want to learn what seems to be influencing to the way children grow up in this suburb. For example how friendly and safe the suburb feels, how much people feel they have a say about what happens in the community, as well as the availability and quality of services, parks, and public transport. Whatever feels most relevant to [name of community]. We also want to know if there have been any significant changes in the local area or suburb over the past few years.

If we can learn more about these issues, we hope to work with communities to help them become places where people like to live and where children grow up happy and healthy.

Because this is a university study we have a formal process we need to go through before we get started. This study has been approved by the research ethics committee at Royal Children's Hospital, Melbourne. This is an important process as it ensures that the research is being conducted in the most appropriate manner. So we have a formal consent process to go through together. Let's take some time to read through the consent document.

The key things to note are:

1. Confidentiality: As outlined in the information and consent form, everything you tell us is confidential, so we won't tell other people who you are, who took part in the focus group or what each of you said. If we use quotes of what people said during the focus group, the quotes are always general enough that no one can identify exactly who said it. The only time we might need to share personal information is when it required by law. This means that if you say something that makes us thing you are being harmed or that you might harm someone, we will have to report it. Because this is a group, it is also important that each of you respect each other's privacy and don't share what others have said with people outside this group.

2. Voluntary: participating in this group is entirely voluntary. If I ask anything you don't feel comfortable answering, that is totally fine. Just let me know. And, if you decide later that you prefer you hadn't told me something, let me know and we can remove it.

3. Recording: I will be taking notes and will also be recording what happens today. We only use the recordings because I can't write as quickly as everyone speaks, so it will help me make my notes better. No-one outside the research team will be able to listen to what you have said.

The information sheet is yours to keep. It has contact details on it if you would like to contact the research team. It also provides contact details of the ethics committee if you have any concerns or complaints about the research.

Do you have any questions, comments or concerns before we start?

2 Community residents (parents of young children)

2.1 About you

To start off, it is good for me to have a little bit of background on each of you here. If we go around the room, I am wondering if you can tell me:

- Your name
- How long you have lived [name of community]
- How many children you have and of what ages
- The type of place you live, that is, in a rental apartment or an apartment you own etc.

2.2 About the community

- Can you describe [name of community]

  Possible prompts

  - *What is it like to live here?*
  - *What sorts of people live here*
  - *What kind of housing is there*

- Are people similar or different in this community?

  Possible prompt

  - *Is there a mix of ages/ethnicity/employment status?*

- What is good about living in [name of community]?

  Possible prompts

  - *What makes it good for families with young children?*
  - *Why did you choose [name of suburb]?*

- What are the problems in [name of suburb]?

  Possible prompt

  - *Are there particular issues for young families?*

2.3 Additional questions (KiCS domains)

Please note that these questions may already have been addressed in the initial discussion about the community. Only ask those questions which haven't already been covered.

2.3.1 Social capital

- How do people get on in this suburb?

  Possible prompt

  What are peoples attitude toward young children?

- Is the community safe or dangerous? Why?
- Do people trust each other or are they suspicious?
- How stable is [name of community]?

  Possible prompt

  - *Do people move in and out of [name of community] or do people stay here for a long time?*
  - *What brings people to [name of community], what makes them stay, what makes them leave?*

2.3.2 Physical

- How is the physical environment

  Possible prompts

  - *Are public buildings in good repair?*
  - *What is the traffic like?*
  - *Are you exposed to pollution?*
- Are there good parks or other places for young children to play?
- How do people get around the in your suburb? (e.g. do they walk, cycle or use cars and/or public transport)

2.3.3 Services

- What is the availability of services in [name of community] (e.g. child care, medical, shopping, transport etc)

  Possible prompts

  - *Are specialist services such as speech therapy, optometry, counselling available?*
  - *Are there schools and preschools in [name of community]? How many and what are they like?*
- What do you think about the services (i.e. what is the quality)?

2.3.4 Governance

- How much do you feel you have a say about what happens in [name of community]?
- Do people come together to address issues?
- Are there strong community leaders?
- Is their leadership inclusive?

2.4 AEDC

- Have you noticed any significant changes in [name of community] over the past few years?

  Possible prompt

  - *How do you think these changes have affected young children in the community?*
- How good is [name of community] for young children compared to neighbouring suburbs?
- Children's outcomes were measured in 2012 (AEDC) and this showed that children in [name of community] were doing the same/better/worse than children in similar communities. How would you explain this? Would you expect to find a similar result now (compared with 2012)?

2.5 Reflection

You have spoken about a range of issues (include list) – which do you think are the most important or significant factors for child development and wellbeing within the community (if you were to rank the top 3)?

2.6 Finish

That's all the questions that I have to ask you. Is there anything else you would like to add?

Thank you for taking the time to talk to me. Our research is dependent on people volunteering to take part in groups like today so we really appreciate the time you have given us.

I have a gift voucher for each of you to in part reimburse you for the time you provided by taking part in the research.

3 Focus Group Questions for practitioners

3.1 About you

To start off, it is good for me to have a little bit of background on each of you here. If we go around the room, I am wondering if you can tell me:

- Job title, role, organisation
- How long you have worked in [name of community]
- How well you know the [name of community]
- Your level of engagement with [name of community]

3.2 About the community

- Can you describe [name of community]

  Possible prompts
  - *What sorts of people live here?*
  - *What kind of housing is there?*
  - *What kind of jobs to people tend to have?*
  - *Do people work locally or commute?*

- What is good about this community?

  Possible prompt
  - *How is it good for families with young children?*

- What are the problems in this community?

  Possible prompt
  - Are there particular issues for families with young children

- Have you noticed any significant changes in your community over the past few years?

  Possible prompt
  - How do you think these changes have affected young children in the community?

- How good is this community for young children compared to similar communities?
- What is your explanation for children in [name of community] doing the same/better/worse on the AEDC in 2012 than children in similar communities in NSW? Would you expect to find a similar result now?

- Do you think there are differences between [on diagonal community] and [off diagonal community] in terms of child development and child wellbeing? If so, what are the key differences?

3.3 Additional questions (KiCS domains)

Please note that these questions may already have been addressed in the initial discussion about the community. Only ask those questions which haven't already been covered.

3.3.1 Social capital

- How do people get on in [name of community]?

  Possible prompt

  - *What are peoples attitude toward young children?*
  - *Are community events held in [name of community]? How well are they attended?*

- Is the community safe or dangerous? Why?

  Possible prompt

  - *What are the safety concerns in [name of community] – domestic violence, illicit drug use, assault and/or burglary?*

- Do people trust each other or are they suspicious?
- How stable is [name of community]?

  Possible prompt

  - *Do people move in and out of [name of community] or do people stay here for a long time?*
  - *What brings people to [name of community], what makes them stay, what makes them leave?*

3.3.2 Services

- What is the availability of services in [name of community] (e.g. child care, medical, shopping, transport etc)
- Are specialist services such as speech therapy, optometry, counselling available?
- Are there schools and preschools in [name of community]? How many and what are they like?
- What do you think about the quality of services (i.e. what is the quality)?
- Are there existing collaborations or networks between services?

3.3.3 Governance

- How much say do residents/workers have about what happens in this community?
- Are there strong community leaders?
- Is their leadership inclusive?

3.3.4 Physical

- How is the physical environment

  Possible prompts

  - *Are public buildings in good repair?*
  - *What is the traffic like?*
  - *Are people exposed to pollution?*
- Are there good parks or other places for young children to play?
- How do people get around the in [name of community]? (e.g. do they walk, cycle or use cars and/or public transport)

3.4 Reflection

You have spoken about a range of issues including…. (list). If you were to rank the top 3, which do you think are the most important or significant factors for child development and wellbeing within the community?

3.5 Finish

That's all the questions that I have to ask you. Is there anything else you would like to add?

Thank you for taking the time to talk to me. Our research is dependent on people volunteering to take part in groups like today so we really appreciate the time you have given us.
