# Supplementary material for: Findings from the Kids in Communities Study (KiCS): A mixed methods study examining community-level influences on early childhood development
Source: PLoS One. 2021 Sep 1;16(9):e0256431. doi: 10.1371/journal.pone.0256431 (PMC8409665; doi:10.1371/journal.pone.0256431)
Supplement: S3 Appendix — (PDF) [file pone.0256431.s003.pdf]

S3 Appendix: Community survey

ID: XXXXXXXX

kids in communities study

THE UNIVERSITY OF MELBOURNE

SURVEY ABOUT <LOCAL COMMUNITY NAME>

This survey is about life in <local community name>. Thanks for taking time to participate! The survey should take you around 10-15 minutes to complete. There are no wrong answers and you don't have to answer every question, but it's really helpful to us if you answer as many questions as you can. We just want **your opinion** and all your answers are strictly confidential.

When you've completed the survey, you'll find a reply-paid envelope in this pack to make it easy to return. If you like, you can answer the survey online instead and not worry about getting to a postbox. The link for the online version is here: https://redcap.mcri.edu.au/kics

COMPLETION INSTRUCTIONS

Please use *blue* or *black* pen.

**Please *tick* the boxes**	**Please write clearly in**
☑	CAPITAL LETTERS
If you make a mistake or want to change anything, just cross out the incorrect response and tick the correct response.	If you make a mistake or want to change anything, just cross out the incorrect response and write your new response just above or below.

Thanks for taking part in the KiCS survey!

If you need assistance with the survey, please call 03 9345 5732. If no-one answers the phone, please leave a message and we will get back to you.

Section A: About your suburb or local area

1. Which suburb do you live in? ____

2. How long have you lived in this suburb? *(Tick one only)*

0-11 months 0☐ 1-5 years 1☐ 6-10 years 2☐ 11+ years 3☐

3. ACTIVITIES AND GROUPS IN YOUR SUBURB OR LOCAL AREA

a. In the past 12 months, which, if any, of the following community groups or activities have you been involved with in your suburb or local area, or elsewhere? *(Tick as many that apply)*

Activity	I have been involved in this activity	
	IN MY SUBURB OR LOCAL AREA	OUTSIDE MY SUBURB OR LOCAL AREA
Child care or school-related group or activity	0☐	1☐
Service club such as Lions or Rotary	0☐	1☐
Ethnic association or club	0☐	1☐
Volunteer organisation or activity	0☐	1☐
Fund raising activity	0☐	1☐
Resident or community action group	0☐	1☐
Church or religious group	0☐	1☐
Arts event as a participant *(e.g., arts and craft class, choir)*	0☐	1☐
Arts event as a spectator *(e.g., concert, theatre, film)*	0☐	1☐
Sport or recreation group, club or activity, *as a participant*	0☐	1☐
Sport or recreation group, club or activity, *as a coach, instructor, committee member, or manager/secretary*	0☐	1☐
Hobby/ interest class, club or group	0☐	1☐
Work-related professional group or association	0☐	1☐
Political or labour union group	0☐	1☐
Public interest meeting, event, or march	0☐	1☐
None of the above	0☐	1☐
Other (please specify) ________________________	0☐	1☐

b. Do you feel there are opportunities to have a real say on local issues that are important to you? *(Tick one only)*

Yes 1☐ No 0☐ Maybe/ don't know/ not sure 2☐

Section B: About places and services in your suburb or local area

This section is about access, availability, and quality of places and services in your suburb or local area.

1. **Which of the following are available in your suburb or local area?** *(Tick the box that best applies to you and your suburb or local area)*

	Yes	No	Do not know/ not sure
There are doctors/ medical clinics in my suburb or local area	1☐	0☐	2☐
There are maternal and child health services available in my suburb or local area	1☐	0☐	2☐
There are child care/ occasional care services available in my suburb or local area	1☐	0☐	2☐
There are preschool/ kindergartens available in my suburb or local area	1☐	0☐	2☐
There are parks and playgrounds in my suburb or local area	1☐	0☐	2☐

2. **On a scale of 1-5 (1 = very poor to 5 = very good), how would you rate the quality of places and services in your suburb or local area?** *(Tick the box that best applies to you and your suburb or local area)*

	1 Very poor	2 Poor	3 Average	4 Good	5 Very good	Do not know/ Not sure
Doctors/ medical clinics in my suburb or local area	1☐	2☐	3☐	4☐	5☐	6☐
Maternal and child health services in my suburb or local area	1☐	2☐	3☐	4☐	5☐	6☐
Child care/ occasional care services in my suburb or local area	1☐	2☐	3☐	4☐	5☐	6☐
Preschools/ kindergartens in my suburb or local area	1☐	2☐	3☐	4☐	5☐	6☐
Parks and playgrounds in my suburb or local area	1☐	2☐	3☐	4☐	5☐	6☐

3. **Which, if any, of the following services do you USUALLY use?** *(Tick one box per line)*

	In my suburb	In neighbouring suburb	In other suburb	Not applicable/ don't use
Doctors/ medical clinics	1☐	2☐	3☐	4☐
Maternal and child health services	1☐	2☐	3☐	4☐
Child care/ occasional care services	1☐	2☐	3☐	4☐
Preschool/ kindergarten	1☐	2☐	3☐	4☐
Parks and playgrounds	1☐	2☐	3☐	4☐

Section C: About your neighbourhood

This section is about your neighbourhood. By neighbourhood, we mean everywhere within a 10-15 minute walk of your home.

1. TRANSPORT

a. In a USUAL WEEK, how many times do you do each of the following? (PUT 0 IF YOU DON'T DO THIS ACTIVITY USUALLY)

Activity	Number of times in usual week
**Walk** as a means of **transport**, such as going to and from work, walking to shops or walking to public transport in your neighbourhood?	
**Walk** for **recreation, health or fitness** (including walking a dog) in or around your neighbourhood?	
**Cycle** as a means of **transport,** such as going to or from work, cycling to the shop or cycling to public transport in your neighbourhood?	
**Cycle** for **recreation, health or fitness** in or around your neighbourhood?	
Take **public transport** from your neighbourhood?	

b. Please estimate the total time (in minutes) you spend doing each of the following activities in a USUAL WEEK e.g., 5 times by 20 minutes = 100 minutes (PUT 0 IF YOU DON'T DO THIS ACTIVITY USUALLY)

Total time in a usual week:	Minutes
**Walk** as a means of **transport**, such as going to and from work, walking to shops or walking to public transport in your neighbourhood	
**Walk** for **recreation, health or fitness** (including walking a dog) in or around your neighbourhood	
**Cycle** as a means of **transport,** such as going to or from work, cycling to the shop or cycling to public transport in your neighbourhood	
**Cycle** for **recreation, health or fitness** in or around your neighbourhood	
Travelling by **public transport**	

2. TRAFFIC SAFETY

How strongly do you agree or disagree with the following statements about your neighbourhood?
(Please tick the box that best applies to you and your neighbourhood)

	Strongly disagree	Disagree	Neither agree or disagree	Agree	Strongly agree	Does not apply to me
There is so much traffic along most nearby streets that it makes it difficult or unpleasant to walk in my neighbourhood	1☐	2☐	3☐	4☐	5☐	6☐
I live on or near a main arterial road or busy throughway for motor vehicles	1☐	2☐	3☐	4☐	5☐	6☐
The speed of traffic on most nearby streets is usually slow (50kph or less)	1☐	2☐	3☐	4☐	5☐	6☐
There are many traffic slowing devices in my neighbourhood (such as speed humps, roundabouts, traffic islands)	1☐	2☐	3☐	4☐	5☐	6☐
When walking in my neighbourhood there are a lot of exhaust fumes (such as from cars, buses)	1☐	2☐	3☐	4☐	5☐	6☐
Most drivers exceed the posted speed limits when driving in my neighbourhood	1☐	2☐	3☐	4☐	5☐	6☐
There are crosswalks and pedestrian signals to help walkers cross busy streets in my neighbourhood	1☐	2☐	3☐	4☐	5☐	6☐

3. CRIME SAFETY

How strongly do you agree or disagree with the following statements about your neighbourhood?
(Please tick the box that best applies to you and your neighbourhood)

	Strongly disagree	Disagree	Neither agree or disagree	Agree	Strongly agree	Does not apply to me
There is a lot of petty crime in my neighbourhood (such as vandalism, shoplifting)	1☐	2☐	3☐	4☐	5☐	6☐
There is a lot of major crime in my neighbourhood (such as armed robberies, break-ins, attacks)	1☐	2☐	3☐	4☐	5☐	6☐
The level of crime in my neighbourhood makes it unsafe to go on walks during the day	1☐	2☐	3☐	4☐	5☐	6☐
The level of crime in my neighbourhood makes it unsafe to go on walks at night	1☐	2☐	3☐	4☐	5☐	6☐
Streets in my neighbourhood are well lit at night	1☐	2☐	3☐	4☐	5☐	6☐

4. NEIGHBOURHOOD SURROUNDINGS

How strongly do you agree or disagree with the following statements about your neighbourhood?
(Please tick the box that best applies to you and your neighbourhood)

	Strongly disagree	Disagree	Neither agree or disagree	Agree	Strongly agree	Does not apply to me
There is lots of greenery around my neighbourhood (trees, bushes, household gardens)	1☐	2☐	3☐	4☐	5☐	6☐
There is tree cover or canopy along the footpaths in my neighbourhood	1☐	2☐	3☐	4☐	5☐	6☐
There are many interesting things to look at while walking in my neighbourhood	1☐	2☐	3☐	4☐	5☐	6☐
My neighbourhood is generally free from litter, rubbish, or graffiti	1☐	2☐	3☐	4☐	5☐	6☐
There are attractive buildings and homes in my neighbourhood	1☐	2☐	3☐	4☐	5☐	6☐
There are many natural sights in my neighbourhood (such as landscaping, views)	1☐	2☐	3☐	4☐	5☐	6☐

5. PEOPLE IN YOUR NEIGHBOURHOOD

a. **How strongly do you agree or disagree with the following statements about your neighbourhood?**
(Please tick the box that best applies to you and your neighbourhood)

	Strongly disagree	Disagree	Neither agree or disagree	Agree	Strongly agree
I often see adults walking in my neighbourhood	1☐	2☐	3☐	4☐	5☐
I often see children walking in my neighbourhood	1☐	2☐	3☐	4☐	5☐
Generally, this is a good neighbourhood to bring up young children	1☐	2☐	3☐	4☐	5☐
People around here are willing to help their neighbours	1☐	2☐	3☐	4☐	5☐
This is a close-knit neighbourhood	1☐	2☐	3☐	4☐	5☐
People in this neighbourhood can be trusted	1☐	2☐	3☐	4☐	5☐
People in this neighbourhood generally don't get along with each other	1☐	2☐	3☐	4☐	5☐
People in this neighbourhood do not share the same values	1☐	2☐	3☐	4☐	5☐
People in this neighbourhood will take advantage of you	1☐	2☐	3☐	4☐	5☐
Given the opportunity, I would like to move out of this neighbourhood	1☐	2☐	3☐	4☐	5☐
Overall, I am very attracted to living in this neighbourhood	1☐	2☐	3☐	4☐	5☐
I feel like I belong in this neighbourhood	1☐	2☐	3☐	4☐	5☐
I visit with my neighbours in their homes	1☐	2☐	3☐	4☐	5☐
The friendships and associations I have made with other people in my neighbourhood mean a lot to me	1☐	2☐	3☐	4☐	5☐

	Strongly disagree	Disagree	Neither agree or disagree	Agree	Strongly agree
If the people in my neighbourhood were planning something I'd like to think of it as something "we" were doing rather than "they" were doing	1☐	2☐	3☐	4☐	5☐
If I needed advice about something I could go to someone in my neighbourhood	1☐	2☐	3☐	4☐	5☐
I think I agree with most people in my neighbourhood about what is important in life	1☐	2☐	3☐	4☐	5☐
I believe my neighbours would help me in an emergency	1☐	2☐	3☐	4☐	5☐
I feel loyal to the people in my neighbourhood	1☐	2☐	3☐	4☐	5☐
I borrow things and exchange favours with my neighbours	1☐	2☐	3☐	4☐	5☐
I would be willing to work together with others on something to improve my neighbourhood	1☐	2☐	3☐	4☐	5☐
I plan to remain a resident of this neighbourhood for a number of years	1☐	2☐	3☐	4☐	5☐
I like to think of myself as similar to the people who live in this neighbourhood	1☐	2☐	3☐	4☐	5☐
I rarely have neighbours over to my house to visit	1☐	2☐	3☐	4☐	5☐
I feel that there is a bond between me and other people in this neighbourhood	1☐	2☐	3☐	4☐	5☐
I regularly stop and talk with people in my neighbourhood	1☐	2☐	3☐	4☐	5☐
Living in this neighbourhood gives me a sense of community	1☐	2☐	3☐	4☐	5☐
People in this neighbourhood are from similar backgrounds	1☐	2☐	3☐	4☐	5☐

b. How common are insults or attacks to do with someone's race, ethnicity, or cultural background in your suburb or local area? *(Tick only one)*

Very common 1☐ Fairly common 2☐ Not very common 3☐ Not at all common 4☐

Section D: About you

1. Are you *(Tick one only)*

Male 0☐ Female 1☐ Other 2☐

2. What is your age group? (years) *(Tick one only)*

18-24 0☐ 25-34 1☐ 35-44 2☐ 45-54 3☐ 55-64 4☐ 65 or over 5☐

3. What is your race, ethnicity or cultural background: ___________________________

4. Are you of Aboriginal or Torres Strait Islander Descent? *(Tick one only)*

Yes 1☐ No 0☐

5. Do you speak a language other than English at home? *(Tick one only)*

Yes 1☐ No 0☐

6. How would you best describe your current employment status? *(Tick one only)*

In full-time work	In part-time or casual work	Unemployed and seeking work	Unemployed and *not* seeking work	A full-time student	In full-time home duties	Unable to work or retired	Any other status (specify)
0☐	1☐	2☐	3☐	4☐	5☐	6☐	

7. What is the highest level of education you have completed? *(Tick one only)*

Less than Year 10	Year 10 or 11	Year 12	Trade / apprenticeship	Certificate/ diploma	Bachelor degree or higher
0☐	1☐	2☐	3☐	4☐	5☐

8. Before tax is taken out, which of the following ranges best describes your household's income, from all sources, over the past 12 months? *(Tick one only)*

Under $30,000	$30,001 - $60,000	$60,001 - $100,000	$100,001 - $150,000	Over $150,000
0☐	1☐	2☐	3☐	4☐

9. Are you a parent or guardian? *(Tick one only)*

Yes 1☐ No 0☐

10. How many children <u>under 18 years old</u> live in your household? *(Please write a number in the relevant box/es)*

None	0-4 years	5-9 years	10-14 years	15-18 years
0☐	1☐	2☐	3☐	4☐

11. Would you like to add any other comments about raising children in your neighbourhood?

Thank you for participating!

To win one of three $300 Coles vouchers please leave your name and contact info:

Name: ____________________

Contact e-mail: ____________________

Competition t&c's are listed here: https://www.mcri.edu.au/kics
