# Supplementary material for: Findings from the Kids in Communities Study (KiCS): A mixed methods study examining community-level influences on early childhood development
Source: PLoS One. 2021 Sep 1;16(9):e0256431. doi: 10.1371/journal.pone.0256431 (PMC8409665; doi:10.1371/journal.pone.0256431)
Supplement: S1 File — (PDF) [file pone.0256431.s004.pdf]

Foundational Community Factors (FCFs) for Early Childhood Development:

A REPORT ON THE KIDS IN COMMUNITIES STUDY

April 2018

murdoch children's research institute
UNIVERSITY OF CANBERRA AUSTRALIA'S CAPITAL UNIVERSITY
UNSW SYDNEY
KiCS kids in communities study
RMIT UNIVERSITY
UNIVERSITY OF SOUTHERN QUEENSLAND
THE UNIVERSITY OF WESTERN AUSTRALIA
THE UNIVERSITY OF MELBOURNE

Acknowledgements

The KiCS collaboration includes a number of Australian and international universities, and government and non-government partner organisations

Lead organisations

Chief Investigators

- Prof Sharon Goldfeld, Centre for Community Child Health, The University of Melbourne, Murdoch Children's Research Institute
- Prof Ilan Katz, The University of New South Wales
- Prof Robert Tanton, The University of Canberra
- A/Prof Sally Brinkman, Telethon Kids Institute, The University of Western Australia
- Prof Geoffrey Woolcock, The University of Southern Queensland
- Prof Billie Giles-Corti, RMIT University

Federal and state government partner organisations

- Australian Government Department of Social Services
- Australian Government Department of Education and Training
- Victorian Department of Education and Training
- Australian Capital Territory Community Services Directorate
- Children's Health Queensland Hospital and Health Service
- South Australian Department for Education and Child Development
- New South Wales Department of Education and Communities
- Queensland Department of Education, Training and Employment
- New South Wales Department of Family and Community Services
- Australian Bureau of Statistics

Non-government partner organisations

- The Smith Family
- Murdoch Children's Research Institute
- Wesley Mission Brisbane
- The Benevolent Society
- Uniting NSW/ ACT

Other collaborating organisations

- The University of British Columbia (Canada)
- Mount Saint Vincent University (Canada)
- University of Ohio (USA)

Funding

The initial work for data collection and analysis was funded by Kids in Communities Study (KiCS), an Australian Research Council (ARC) Linkage Grant (LP130100411) led by The University of Melbourne. From this work, the Australian Government Department of Social Services (DSS) funded the development of community-level foundational community factors (FCFs) for early childhood development (ECD) and draft manual of community FCF measures and methodologies (**KiCS FCF Manual**). The DSS and partner organisations are gratefully acknowledged for their generous in-kind and financial support, and input into data collection methods, assistance with sourcing data, engaging with the community, and ethics processes. Prof Sharon Goldfeld (Lead Chief Investigator) is supported by a National Health and Medical Research Council (NHMRC) Career Development Fellowship (1082922), Prof Billie Giles-Corti by a NHMRC Senior Principal Research Fellow Award (1107672), and A/Prof Sally Brinkman by an Early Career Research Fellow Award (1090146).

Contributors

We would like to thank those who have contributed to the project, including:

Researchers

- Dr Karen Villanueva, Murdoch Children's Research Institute, RMIT University
- Ms Ju-Lin Lee, Murdoch Children's Research Institute
- Ms Rachel Robinson, Murdoch Children's Research Institute
- Ms Anna Jones, The University of New South Wales
- Ms Genevieve Smith, Murdoch Children's Research Institute
- Dr Melanie Dare, The University of Canberra
- Mr Dominic Peel, The University of Canberra
- Ms Ashleigh Wilson, Telethon Kids Institute
- Ms Alanna Sincovich, Telethon Kids Institute
- Ms Michelle Tennant, Murdoch Children's Research Institute
- Ms Abbe Moriarty, Murdoch Children's Research Institute
- Ms Amanda Alderton, Murdoch Children's Research Institute

Australian Government Department of Social Services

- Ms Emma Snelson
- Ms Lisha Jackman
- Ms Yvonne Kwan
- Ms Kate Keane

We are thankful to our many partner investigators, students, stakeholders, and participants, for providing their time and assistance to the project.

Suggested citation

This is a project of the Kids in Communities Study (KiCS) collaboration. This report was written by members of the KiCS research team. If you have any queries about this study, please contact kics.study@mcri.edu.au

Goldfeld, S., Villanueva, K., Lee, J.L., Robinson, R., Moriarty, A., Peel, D., Tanton, R., Giles-Corti, B., Woolcock, G., Brinkman, S., Katz, I. (2017). Foundational Community Factors (FCFs) for Early Childhood Development: A report on the Kids in Communities Study

Contents

Tables iv
Figures iv
Abbreviations v

About this report 1
Executive Summary 2

PART 1 **About KiCS** 9

1.1 The KiCS collaboration 9
1.2 Selection of study sites 10
1.3 Mixed Methods for Community Investigations 12
1.3.1 Data collection 12
1.3.2 Community Investigations 17
1.3.3 Data cleaning and preparation 19
1.4 Data analysis - Developing the FCFs 19
1.4.1 Stage 1 of data analysis: Differentiating factors 19
1.4.2 Stage 2 of data analysis: Important factors 22
1.4.3 Summary of FCF development 23

PART 2 **KiCS findings** 24

2.1 Field work information 24
2.2 The differentiating FCFs 26
2.3 The important FCFs 27
2.4 Measuring the FCFs... towards better 'indicators'? 28
2.5 Moving beyond indicators... the importance of mixed methods 30

PART 3 **Differentiating FCFs** 31

3.1 Income 32
3.1.1 Relationship with ECD 32
3.1.2 What KiCS found 32
3.1.3 Limitations with measurement and use 33
3.2 Highest level of schooling 34
3.2.1 Relationship with ECD 34
3.2.2 What KiCS found 34
3.2.3 Limitations with measurement and use 35
3.3 Gentrification 36
3.3.1 Relationship with ECD 36
3.3.2 What KiCS found 36
3.3.3 Limitations with measurement and use 37
3.4 Housing affordability 38
3.4.1 Relationship with ECD 38
3.4.2 What KiCS found 38
3.4.3 Limitations with measurement and use 39
3.5 Housing tenure (stability) 40
3.5.1 Relationship with ECD 40
3.5.2 What KiCS found 40
3.5.3 Limitations with measurement and use 41
3.6 **Public housing** 42
3.6.1 Relationship with ECD 42
3.6.2 What KiCS found 42
3.6.3 Limitations for measurement and use 43
3.7 Housing density 44
3.7.1 Relationship with ECD 44
3.7.2 What KiCS found 44
3.7.3 Limitations with measurement and use 45
3.8 Stigma 46
3.8.1 Relationship with ECD 46
3.8.2 What KiCS found 46
3.8.3 Limitations with measurement and use 47
3.9 Service reputation (primary schools) 48
3.9.1 Relationship with ECD 48
3.9.2 What KiCS found 48
3.9.3 Limitations with measurement and use 49
3.10 Perceived ECEC availability 50
3.10.1 Relationship with ECD 50
3.10.2 What KiCS found 50
3.10.3 Limitations with measurement and use 51
3.11 Perceived crime 52
3.11.1 Relationship with ECD 52
3.11.2 What KiCS found 52
3.11.3 Limitations with measurement and use 52
3.12 Historical events 54
3.12.1 Relationship with ECD 54
3.12.2 What KiCS found 54
3.12.3 Limitations with measurement and use 55
3.13 Local decision-making 56
3.13.1 Relationship with ECD 56
3.13.2 What KiCS found 56
3.13.3 Limitations 56

PART 4 **Important FCFs** 57

PART 5 **Discussion** 62

5.1 Strengths 63
5.2 Limitations 63
5.2.1 Challenges with measurement 63
5.2.2 Generalisability 65

PART 6 **Conclusion** 66

6.1 The beginning of FCFs for ECD... not the end 66

PART 7 **Appendices** 68

7.1 Appendix 1. Full suite of community factors explored 68
7.2 Appendix 2. Measures used to explore the Differentiating FCFs 74
7.3 Appendix 3. Review of results with Triangulation 75

PART 8 **References** 86

Tables

Table 1.	The list of FCFs	7
Table 2.	Snapshot of local communities	11
Table 3.	Summary of KiCS community domains and sub-domains and methodologies	13
Table 4.	Assessment of 'differences' between on- and off-diagonals	20
Table 5.	Summary of field work in local communities	24
Table 6.	Differentiating Foundational Community Factors – what KiCS found	26
Table 7.	Important Foundational Community Factors – what KiCS found	27
Table 8.	OECD selection criteria for indicator development	28
Table 9.	Assessment of KiCS 'indicators' against selection criteria	29
Table 10.	Important FCFs for ECD	57

Figures

Figure 1:	The Kids in Communities Study conceptual framework	2
Figure 2:	Classification of on- and off-diagonal local communities	4
Figure 3.	Developing the foundational community factors and next stages	6
Figure 4:	Example of a heat map matrix showing results within and across matched-disadvantaged community pairs (stage 1 data analysis)	22

Abbreviations

ABS	Australian Bureau of Statistics
ACT	Australian Capital Territory
AEC	Australian Electoral Commission
AEDC	Australian Early Development Census
AIFS	Australian Institute of Family Studies
ARC	Australian Research Council
AURIN	Australian Urban Research and Infrastructure Network
DSS	Australian Government Department of Social Services
DV1	Developmentally vulnerable on at least one AEDC domain
ECD	Early Childhood Development
ECEC	Early Childhood Education and Care
EDI	Canadian Early Development Instrument
FCF	Foundational Community Factor
GIS	Geographic Information Systems
KiCS	Kids in Communities Study
MS	Microsoft
NHMRC	National Health and Medical Research Council
NSW	New South Wales
OECD	Organisation for Economic Co-operation and Development
Off-	Off-diagonal negative
Off+	Off-diagonal positive
OnAdv	On-diagonal advantaged
OnDis	On-diagonal disadvantaged
QLD	Queensland
SA	South Australia
SA2	Statistical Area Level 2
SEIFA-IRSD	Socio-economic Index for Areas Index of Relative Socio-economic Disadvantage
SES	Socio-economic Status
VIC	Victoria

About this report

This report summarises a series of promising (draft) foundational community factors (FCFs) for early childhood development (ECD), which is based on findings from the Kids in Communities Study (KiCS), an Australian investigation of community-level factors influencing ECD. FCFs are the community-level factors that lay the foundations of a good community for young children.

In this report, we describe the overall background and methodology of KiCS, however more detail can be found in the KiCS protocol publication.[3] The **KiCS FCF Manual** is a complementary document to this report, which provides further information about how communities can measure the differentiating FCFs (a subset of the FCFs) recommended for measurement.

Supplementary material

This report explains *why* we chose the list of FCFs, while the KiCS FCF Manual contains the '*how-to*' of collecting the set of differentiating FCFs only. This data icon indicates when we highly recommend reading the **KiCS FCF Manual**.

Executive Summary

ECD research has mostly focused on individual, family, and school factors, but has largely ignored community-level influences. The research into neighbourhood or community effects on children shows that disadvantage is often geographically concentrated and inter-generational.[4] The community level can impact the healthy development of children, particularly on the resources that are available to families to promote good development.[5, 6] Research shows that in disadvantaged communities, lack of resources and opportunities can result in worse child development outcomes that can persist from one generation to the next. However, there are also many factors—such as engaged parents and families, active community organisations, and neighbourhoods that are safe to walk in and have good places to play—that can promote healthy child development even in lower income communities.

Global agencies (e.g. WHO, UNICEF) also recognise that early childhood is one of the most critical development periods, with positive early childhood development (ECD) powerfully contributing to the productivity of society at large.[7] Alongside global ECD agendas, current Australian and global 'child-friendly city' agendas and place-based initiatives seek to promote and protect child wellbeing through healthy communities. Some examples of Australian place-based efforts include Communities for Children,[8] Opportunity Child,[9] and Logan Together. [10] These place-based initiatives advocate the need for healthy communities for families and children and employ local decision making models in order to tailor interventions to the local population. However, for more effective place-based interventions, evidence and data are needed to make informed recommendations required to leverage policy change for healthier ECD.

The Kids in Communities Study (KiCS)

The Kids in Communities Study (KiCS) is an Australian Research Council funded study that used a range of methods to investigate the potential influence of community-level factors on early childhood developmental outcomes measured by the Australian Early Development Census (AEDC). Community-level factors in five domains were investigated—**physical environment, social environment, socio-economic factors, access to services, and governance** (see **Figure 1**).

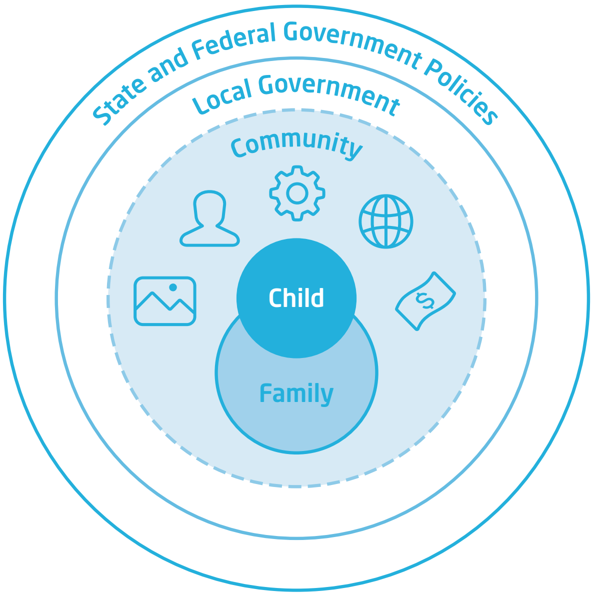

Physical domain: Parks, public transport, road safety, housing

Social domain: Social capital, neighbourhood attachment, crime, trust, safety

Service domain: Quantity, quality, access and coordination of services

Governance domain: Citizen engagement, leadership, decision making and implementation

Socio-economic domain: Community SES

Figure 1: The Kids in Communities Study conceptual framework (reproduced from Goldfeld et al. 2015)(11)

KiCS aimed to better understand local community-level factors that are consistently related to better outcomes for children. Most importantly, the study aimed to determine which of these are the most measurable and modifiable community-level factors that influence children's developmental and health outcomes across communities. This provided the basis for a series of promising (draft) **foundational community factors** (and indicators) that will be further testable in communities around Australia.

Creating foundational community factors and not just indicators

Globally there is interest in measuring the progress of societies.[12] While there are many definitions of what an indicator is, the consensus is that an indicator provides a **summary indication of the condition or problem, and permits the observation of progress or change**.[9] Indicators are one way to do this, and can help provide a summary indication of the condition or problem and permit the observation of progress or change. Evidence-informed indicators can help strengthen community engagement and development, assist with prioritising effort, and help inform policy recommendations using the best local data.

*'An **indicator** is a statistic or parameter, that, tracked over time, provides information on trends in the condition of a phenomenon and has significance extending beyond that associated with the properties of the statistic itself'*[2]

Not all the KiCS foundational community factors fit the global definition of an indicator. With further research, some of these factors can be turned into an 'indicator'

While KiCS initially set out to create robust community indicators for ECD (specific, measurable and repeatable over time),[2] we faced challenges with the complexity of different community contexts, and limitations with quantitative measurement and modelling (e.g. small number of communities in KiCS). Indicators have traditionally been quantitative (rather than qualitative) and such challenges limited the number of quantitative indicators from the study. However, a key strength of KiCS was the rich qualitative data collected. The mixed methodologies used in KiCS helped to triangulate qualitative and quantitative findings and provided an in-depth understanding of the community and the factors associated with ECD outcomes in communities. As such, we recommended developing a set of evidence-informed 'foundational community factors'. These factors are derived from findings from KiCS (i.e. community-level factors related to ECD) and can lend themselves to quantitative and/or qualitative measurement. Some factors may be 'indicators', whilst others require further research to be developed into indicators.

'Foundational community factors' for ECD

Foundational community factors (FCFs) lay the foundations of a good community for young children. They are the 'key ingredients' to create a healthy community for ECD. Foundational community factors can be measured quantitatively (e.g. surveys, existing data) or qualitatively (e.g. focus groups, interviews).

***Foundational Community Factors** are factors that lay the foundations of a good community for young children. The acronym '**FCF**' is used throughout this report*

Why are foundational community factors important?

Foundational community factors will assist in better understanding what facilitates or hinders ECD at the community level. Local information on the FCFs can help contribute to decision making and interventions that move beyond the individual-level, which has shown limited *sustained* success, to the broader community-level (e.g. place-based initiatives). This has the potential to benefit *many* children and families in the long-term.

In particular:

- The FCFs are based on evidence from KiCS, which means that critical points of intervention for creating better environments for children's health and wellbeing are informed by research. This can empower communities to better understand and recognise their resources and opportunities to improve, helping to direct community effort into areas that make the most sense. It allows communities to move beyond anecdotal information to a discussion grounded in evidence about how the community is tracking to inform place-based initiatives.(13)
- The FCFs can help communities strengthen stakeholder engagement and development, and inform policy recommendations using the best local data. For example, they can be used to inform and involve local residents and organisations, identify key issues, discuss priorities, and plan future directions for their community.(13)

How did we develop the foundational community factors?

Developing FCFs involved exploring a mix of quantitative and qualitative measures of community-level factors in a small number of local communities in New South Wales, Victoria, Queensland, South Australia, and the Australian Capital Territory. Overall analysis of these measures involved a two-staged approach to develop a list of draft FCFs for ECD.

Selecting study communities

Twenty five local communities (suburbs) in areas of advantage and disadvantage were selected in a number of local government areas (communities) across five states and territories in Australia (VIC, NSW, QLD, SA, and the ACT). Selection was based on community socio-economic status (SES) using the ABS Socio-economic Index for Areas (SEIFA) and ECD using the AEDC, a population measure of child development. A local community (suburb) "diagonality type" was created i.e. those performing better or worse ("off-diagonal"), or as expected ("on-diagonal") on the AEDC relative to their SES (see **Figure 2**).

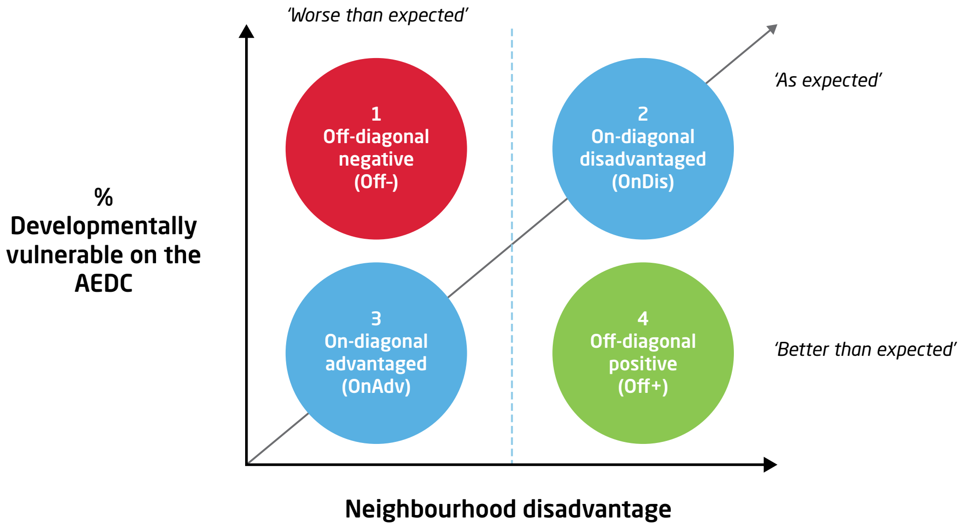

Figure 2: Classification of on- and off-diagonal local communities (adapted from Tanton et al. 2015).(14)

Blue: On-diagonal local communities; **Red or Green:** Off-diagonal local communities
Dotted line: matched dis/advantaged community pairs
AEDC: Australian Early Development Census
Developmentally vulnerable: % developmentally vulnerable on at least 1 (of 5) AEDC domains
Neighbourhood disadvantage: Australian Bureau of Statistics Socio-Economic Index for Areas – Index for Relative Socio-economic Disadvantage (IRSD)

Data collection

In 2015-2017, quantitative and qualitative data were collected from each local community. The number of focus groups and interviews in each area varied. The following data collections were proposed for each local community:

- Semi-structured interviews with 8-15 stakeholders within each local government area
- Focus groups with local parents of young children aged 0-8 years
- Focus groups and surveys with local service providers of early years services
- Community surveys distributed to 1000 general community residents in each local community
- Mapping of neighbourhood design and local amenities and services using Geographic Information Systems (GIS) software
- Park audits to capture features and amenities for each park within the local community
- Collection of approximately 120 policy documents to better understand local governance processes that may influence early childhood outcomes
- Existing socio-demographic data from the Australian Bureau of Statistics (ABS), and early childhood education and care data.

Analysing data

A two-staged approach to analysis was undertaken. Factors 'within' communities, and 'across' communities were explored:

Stage 1: Differentiating factors focused on qualitative and quantitative factors that differentiated local community diagonality status. That is, we analysed pairs of neighbouring on- and off-diagonal local communities matched on disadvantage (14 local communities matched on disadvantage i.e. seven community pairs). This provided a sense of why one local community had better ECD outcomes than its neighbouring local community despite both experiencing disadvantage. Factors considered as consistently differentiating were those that appeared in at least four of the seven matched disadvantaged community pairs.

Stage 2: Important factors explored qualitative data only (focus groups and interviews) and identified community-level factors emerging as important across all 25 local communities regardless of its diagonality status. That is, are there any community-level factors that are consistently noted as important for families and young children? For example, if public transport, walkability, traffic, park access and quality, service access and quality, did not differentiate community pairs (from Stage 1), it does not mean that these factors aren't important for the community. While there is likely to be differences between local communities, factors considered to be consistently important for ECD were those that appeared in at least 16 of the 25 local communities. A summary of data collection, data analysis and outputs is outlined in **Figure 3**.

Together these factors form the list of FCFs for ECD (see **Table 1**).

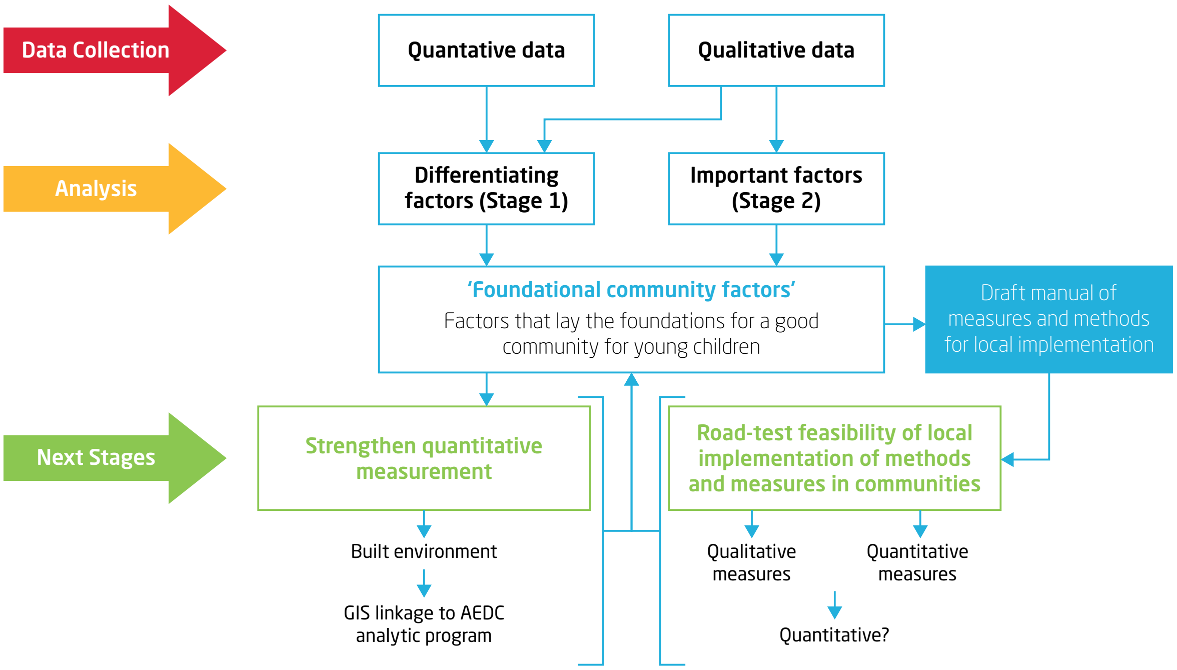

Figure 3. Developing the foundational community factors and next stages

Foundational community factors for ECD

The differentiating (Stage 1) and important FCFs (Stage 2) are presented in **Table 1**. There are a number of limitations (see **Limitations section 5.2**) that need to be considered when interpreting the FCFs (see **Part 2**).

Some of the quantitative differentiating factors (from Stage 1) lend themselves to being an indicator (e.g. Income, highest level of schooling, housing tenure (stability), public housing). Currently, we have not recommended quantitative measurement of the important factors (i.e. Stage 2) until further research is conducted. Eventually, all foundational community factors may be developed into an indicator, measured either objectively (e.g. by Geographic Information Systems) or subjectively (e.g. by surveys), however stronger quantitative indicators for ECD requires further research. Nevertheless, KiCS provides in-depth consultation with 25 local communities, which provides rich insight into how community factors act to influence ECD.

Table 1. The list of FCFs

**Differentiating Foundational Community Factor – What KiCS found differentiates disadvantaged local communities doing well or poorly on ECD**		
1	Income#	Median household income[1] and degree of economic diversity[2] is greater in disadvantaged areas doing well on ECD
2	Highest level of schooling#	There is a higher proportion of the population that have completed Year 12 or equivalent[1] in disadvantaged areas doing well on ECD
3	Gentrification	Relatively higher income (but still disadvantaged) families are moving into disadvantaged areas doing well on ECD, resulting in the displacement of more disadvantaged groups[2]
4	Housing affordability	Housing is perceived as more affordable in disadvantaged areas doing well on ECD[2]
5	Housing tenure (stability)#	There is a lower proportion of renters compared to private home owners in disadvantaged areas doing well[1]
6	Public housing#	There is a lower proportion of public renters[1] and less perceived presence of public housing[2] in disadvantaged areas doing well on ECD
7	Housing density*	There is a lower proportion of high rise (three or more storeys)[1] and perceived fewer high rise density dwellings (vs low rise housing developments)[2] in disadvantaged areas doing well on ECD
8	Stigma	Negative reputation of a local community[2] is less in disadvantaged areas doing well on ECD
9	Perceived primary school reputation	Perceptions of primary school quality were better in disadvantaged areas doing well on ECD[2]
10	Perceived Early Childhood Education and Care (ECEC) availability	There was more perceived Early Childhood Education and Care (ECEC) service availability in disadvantaged areas doing well on ECD[2]
11	Perceived crime	There was less perceived crime in disadvantaged areas doing well on ECD[2]
12	Historical events	The response of leaders to events that bring local community members together[2] is greater in disadvantaged areas doing well[2]
13	Local decision-making	As a result of local decision-making, 'novel approaches' or locally tailored initiatives or solutions (including any with a focus on social capital) have been developed in the community doing well[2]
**Important Foundational Community Factor – What KiCS found is important for communities[2]**		
14	Physical access to services	Reported instances of ability to get to services
15	Walkability	Perceived walkability to facilities and services was seen as important for physical access
16	Public transport availability	Perceived presence of/access to public transport was seen as important for easy access within the suburb
17	Traffic exposure	Being away from traffic within the suburb is an important factor for children being safe
18	Public open space – availability and quality	Having parks in the suburb was seen as important for young children and families. Having good quality parks was seen as important for use, play and social interaction
19	Facilities – availability and diversity	Having a range of family-friendly destinations and activities is important for young families and children
20	ECEC cost	Perceived affordability of ECEC is considered important and affects use
21	Leadership	The presence of local champions, leaders and boundary spanners driving local governance
**Encouraging Important FCF – Analysis incomplete[2]**		
22	Service Co-ordination	Co-ordination of services in a local community
23	Sense of community	Reported strong neighbourhood attachment or sense of belonging and pride in being connected to a local community
24	Natural environments	Natural spaces are seen as important for young families

[1]Quantitative; [2]Qualitative; *related to Public housing; #indicator; ECD: Early childhood development

One-size fits all? Not always...

While efforts were made to explore consistent factors that differ between on- and off-diagonal local communities, our findings highlight that neighbourhood effects on ECD cannot always be generalised to all communities or all groups. Qualitative methods can be used to better understand if and why a FCF is an important priority area for the community, and what can be done to improve it. Therefore, the quantitative data provides the 'what' and the scale of the problem; and the qualitative data provides the 'why' and what steps can be taken to try to improve outcomes. This emphasises the richness and value of having localised information to better understand the local context and how a range of factors might be operating. Such information will inform more specific place-based interventions at the local level, in particular, those most likely to be responsive and "work" in that community.

Quantitative data provides the 'what' and scale of the problem. Qualitative data provides the 'why' and what steps can be taken to try to improve outcomes.

Having both qualitative and quantitative methods can provide a more in-depth understanding of the FCFs associated with ECD outcomes in communities

What next?

The KiCS FCFs are the result of a "deep dive" into 25 local communities. The level of depth has resulted in exceptional qualitative data but with some limitation on quantitative data given the relatively small sample size. Similarly, the study has produced a limited set of indicators, and there are challenges we face in terms of robustness and replicability. This substantive work has led to two recommendations for further work (see **Figure 3**) to: 1) test the utility of the foundational community factors in communities; and: 2) strengthen the quantitative indicators through further analyses. This report presents a set of promising (draft) FCFs for ECD and highlights measurement of a subset of FCFs (i.e. the most promising FCFs identified from Stage 1 differentiating factors analysis) in the supplementary **KiCS FCF Manual** that can be tested by interested communities. The intention was to describe the measures and methods for the most promising subset of draft community measures that communities can measure and use based on the data so far, rather than select a large set that may not be feasible for users to measure.

PART 1 About KiCS

Healthy child development is the foundation for human capital and the basis for future community and economic development.[15] A large and growing body of research emphasises the importance of the prenatal and early years for health and developmental outcomes throughout the life course.[11] For a growing number of children, sub-optimal developmental trajectories are well established by the time they start school, and become increasingly difficult and costly to modify with the passage of time.[16]

Alongside this research, previous research demonstrates the important influence the local environment has on the capacity of families to raise their children in ways that promote good developmental outcomes.[11] ECD research has mostly focused on individual, family, and school factors, but has largely ignored community-level influences. Yet socio-ecological frameworks of ECD consistently recognise the community context as an important level of influence.[17, 18] The research into neighbourhood or community effects on children shows that disadvantage is often geographically concentrated and inter-generational.[4] Other research suggests that neighbourhood poverty and demography can be influenced by community-level factors that affect the functioning of families and children,[5, 6] particularly on the resources that are available to families to promote good development.[5, 6] Despite this growing body of research, there is still limited understanding of the modifiable community-level factors likely to benefit outcomes for young children.

What is it about your community that makes a difference to ECD?

To address this gap, KiCS sought to investigate community-level influences on ECD. KiCS is based on an ecological view of child development,[17] which is the understanding that children's development is impacted by many factors working at different levels of society, including the child's family, the community, and local, state and federal government policies. This theory shows how children's development is influenced by everyday interactions between children and their economic, social, physical and political environments.

1.1 The KiCS collaboration

The KiCS collaboration was established in 2007 to investigate community-level socio-economic, demographic and social processes using a multi-disciplinary approach. The conceptual framework that guides KiCS was derived from the social and health sciences, and includes **five community domains** of influence: services, social, socio-economic, physical environment and governance (see **Figure 1**). The study capitalises on the unique availability of the Australian Early Development Census (AEDC) based on the Canadian Early Development Instrument (EDI). These instruments are population measures of early childhood development completed by teachers about students in the first year of school. In 2009 the Australian data were available as a census for 98.5% of the estimated five year old population. Data were collected again in 2012 with an estimated 96.7% of the target population.[16] This data provides a measure of early childhood development at the community (suburb or town) level.

The KiCS collaboration received competitive funding in 2010 to develop and pilot a set of pragmatic community-level measures and methods.[19] These measures were tested in two suburbs (local communities) within one local government area in Melbourne. The pilot resulted in: 1) a proposed methodological approach for determining communities that have AEDC outcomes that differ significantly from that predicted by their socio-economic status (off-diagonal communities); and: 2) methods for measuring community-level domains by testing practical approaches, together with an analysis of secondary data.

With further funding from the Australian Research Council (ARC) and a range of government and non-government organisations, including the Australian Government Department of Education and Training, KiCS Phase 2 extended the methods and findings of the pilot study into a multi-method investigation of community-level factors potentially influencing young children's health and development in a number of communities across five Australian states and territories (Victoria (VIC); New South Wales (NSW); Queensland (QLD), South Australia (SA), and the Australian Capital Territory (ACT)). KiCS is a cross-sectional mixed methods study aimed at better understanding local community-level factors that are consistently related to better outcomes for children.(3) Most importantly, we set out to determine which of these are the most measurable and modifiable community-level factors that influence children's developmental and health outcomes across communities. The findings of KiCS provided the basis for a series of draft foundational community factors that will be further testable in communities around Australia.

1.2 Selection of study sites

In KiCS, there are 25 AEDC local communities (suburbs), clustered within 11 AEDC communities (local government areas). The selection and analysis of study sites (local communities or suburbs) was crucial to the development of the draft FCFs. The methodology for local community eligibility and selection is described in detail elsewhere,(14) but briefly outlined here. Local communities (suburbs) were selected based on their SES and AEDC scores. The 2009 and 2012 national AEDC data and the Australian Bureau of Statistics (ABS) Socio-economic Index for Areas Index of Relative Socio-economic Disadvantage (SEIFA-IRSD) have been paired to identify off-diagonal local communities and adjacent on-diagonal counterparts. Using a matrix of the AEDC and SEIFA-IRSD scores (quintile to quintile cross-tabulation) on- and off-diagonal local communities were identified (see **Figure 2** in Executive Summary)

What do we mean by 'local community'?

Our definition of 'local community' aligns with the AEDC nomenclature and geographic boundaries. The size of a local community varies, but in metropolitan and large regional areas, it equates to a 'suburb' (approximately 10,000 persons per area on average) as defined by the Australian Bureau of Statistics geographic boundaries. Local communities are clustered within larger 'communities', or local government areas.

While the term 'community' may refer to a place or group of people with something in common, and 'neighbourhood' concerns the geographic construct or boundaries, for KiCS, our definition of 'local community' aligns with the AEDC nomenclature and geographic boundaries. The AEDC results are publicly reported as an area-level aggregate termed 'local community'; the size of which varies, but in metropolitan and large regional areas, equates to a 'suburb' (approximately 10,000 persons per area on average).(20) AEDC local communities (suburbs) are the unit of investigation because these are the smallest areas for which AEDC data are publicly available and pockets of disadvantage can be hidden when larger areas are used. AEDC local communities are located within larger AEDC 'communities' or local government areas.

a. On-diagonals perform 'as expected'

What is an on-diagonal advantaged local community?
Advantaged doing well: A local community with good AEDC scores and high SES

What is an on-diagonal disadvantaged local community?
Disadvantaged doing poorly: A local community with poor AEDC scores and low SES

b. Off-diagonals perform 'better (or worse) than expected'

What is an off-diagonal negative local community?
Advantaged doing poorly: A local community with poor AEDC scores despite having high SES

What is an off-diagonal positive local community?
Disadvantaged doing well: A local community with good AEDC scores despite having low SES

Local communities with 'better or worse' ECD outcomes than expected (off-diagonal) were mapped using Geographic Information Systems (GIS) software to identify on-diagonal local communities (those doing 'as expected' on ECD) that were geographically close (i.e. within the same or proximate local government areas). It was assumed that local communities in close proximity to each other are likely to share services (e.g. childcare, early childhood programs) and governance arrangements – that is they are subject to the same policies and decision making processes in local, state and federal government contexts. Of the 25 local communities, 13 were considered off-diagonal, and 12 on-diagonal (see **Table 2**).

For this report, local communities of focus are those *matched on SES*. For example, a matched-disadvantaged community pair has a disadvantaged local community with poor AEDC outcomes (Disadvantaged doing poorly) and a disadvantaged local community with good AEDC outcomes (Disadvantaged doing well). This is important because a 'true' comparison is an off-diagonal matched with an on-diagonal, both holding the same SES. Although there were 25 local communities, there were seven matched-disadvantaged community pairs (therefore, 14 AEDC local communities), and one matched-advantaged community pair (therefore, two AEDC local communities).

Table 2. Snapshot of local communities

			Geographic region		Off-diagonal		On-diagonal	
	State/ Territory	Local communities	Urban	Regional	Positive	Negative	Advantaged	Disadvantaged
		n=25	n=18	n=7	n=8	n=5	n=3	n=9
1	VIC	6	3	3	1	2	2	1
2	NSW	6	6	0	3	0	0	3
3	SA	4	4	0	1	1	0	2
4	QLD	6	2	4	2	1	0	3
5	ACT	3	3	0	1	1	0	1

1.3 Mixed Methods for Community Investigations

KiCS explored community factors that consistently influenced ECD outcomes. Between 2015 and 2017 a mix of qualitative and quantitative methods was used to explore community factors conceptualised within five community domains of influence: physical, service, social, and socio-economic and governance domains; the conceptual framework (see **Figure 1** in Executive Summary). The study design is described fully elsewhere,[(3)] but briefly described here. The Melbourne Royal Children's Hospital Human Research Ethics Committee (30016) provided ethics approval, and further ethics approvals were received from other states and territories if required.

1.3.1 Data collection

KiCS was a large, mixed methods exploratory study involving the measurement of many community factors that were hypothesised to influence ECD. Each of the five community domains had a number of community factors clustered within sub-domains (21 in total). These sub-domains are not mutually exclusive, rather they complement each other, and may overlap (i.e. cross-domains). Both qualitative and quantitative data were collected to provide a better understanding of the complex and dynamic nature of the community context.

The qualitative research in this study explored factors that have previously been difficult to capture due to limited availability of existing data. Qualitative findings provide a deeper understanding to provide context and depth to the quantitative findings and allow us to generate and test theories with quantitative data.

The methodological approach was selected on the basis that it is suitable to the subject being studied and is most likely to achieve the five intellectual goals outlined by Maxwell (2013), with emphasis provided by Bazeley [(Cited in 21)].

- Understanding the *meaning*, for participants in the study, of the events, situations, experiences, and actions they are involved with or engage in.
- Understanding the particular *contexts* within which the participants act, and the influences that this context has on their actions.
- Understanding the *process* by which events and actions take place.
- Identifying *unanticipated* phenomena and influences, and generating new, grounded theories about the latter.
- Developing *causal* explanations.

A summary of the community domains and sub-domains are available in **Table 3**. The following data collections were undertaken to measure community factors and described in more detail in **Section 1.3.2**:

a. Qualitative

- Semi-structured interviews with 8-15 stakeholders within each local government area
- Focus groups with local parents of young children aged 0-8 years
- Focus groups and surveys with local service providers of early years services
- Community surveys distributed to 1000 general community residents in each local community.

b. Quantitative

- Mapping of neighbourhood design and local amenities and services using Geographic Information Systems (GIS) software
- Park audits to capture features and amenities for each park within the local community

- Collection of approximately 120 policy documents to better understand local governance processes that may influence early childhood outcomes
- Existing socio-demographic data from the Australian Bureau of Statistics (ABS), and Early Childhood Education and Care (ECEC) data.

Table 3. Summary of KiCS community domains and sub-domains and methodologies

	Community domain and sub-domain/s	Description	Policy document[2]	Stakeholder interview[2]	Parent focus group[2]	Practitioner focus group[2]	Service survey[2]	Community survey[2]	GIS & park audit[1]	Service information[1]	Community demographics[1]
**PHYSICAL DOMAIN**											
1	**Public open space**	Objective counts, size, type, quality, and proximity to green space (e.g. parks), blue space (e.g. water bodies such as beaches).[1] Perceptions of public open space.[2]		●	●	●		●	●		
2	**Public transport**	Objective counts and proximity to bus, tram, rail/train, and ferry stops.[1] Perceptions of public transport.[2]		●	●	●		●	●		
3	**Traffic exposure**	Objective exposure to traffic volume (high vs. low). Perceptions of traffic exposure.[2]		●	●	●		●	●		
4	**Housing**	Objective residential density (number of dwellings/residential land area) and proportion of high-rise (three or more storeys) vs. low-rise.[1] Perceptions of housing.[2]		●	●	●		●	●		●
5	**Destinations and Services**	Objective counts of and proximity to places/ facilities/ destinations such as services, child care, libraries, community centres, and recreation venues.1 Perceptions of destinations and services.[2]		●	●	●	●	●	●	●	
6	**Walkability**	Objective walkability (density, mixed use, connectivity).[1] Perceived ease or difficulty of getting to and from destinations and services- i.e. how 'pedestrian friendly' or 'walkable' the community is.[2]		●	●	●		●	●		
7	**Crime/ incivilities**	Objective crimes against the person in public or property, total crime rate.[1] Perceptions of crime.[2]		●	●	●		●	●		●

	Community domain and sub-domain/s	Description	Policy document[2]	Stakeholder interview[2]	Parent focus group[2]	Practitioner focus group[2]	Service survey[2]	Community survey[2]	GIS & park audit[1]	Service information[1]	Community demographics[1]
**SOCIAL DOMAIN**											
**8**	**Social capital/ties**										
	Networks	Bonding, bridging and linking capital- i.e. relationships, interactions, and connections with people.[2]			●	●		●			
	Participation	Whether people participate in events and activities.[2]		●	●	●		●			
	Trust	Personalized trust (feeling able to trust other people within the community) and generalized trust (feeling able to trust/have confidence in institutions).[2]			●	●		●			
	Perceptions of community diversity	Perceptions of whether the community is homogeneous or diverse.[2]		●	●	●		●			
**9**	**Crime**										
	Community response to crime	Community response to crime: how people work together within the community in response to crime or perceived crime risk.[2]		●	●	●		●			
	Parental response to crime	Parent perception of crime and safety: the impact of parental views on neighbourhood safety on their parenting behaviour.[2]			●						
	Perceptions of neighbourhood safety	Perceptions of how safe the community feels.[2]		●	●	●		●			
	Domestic violence and child protection	Perceptions and rates of domestic violence and number of children notified in child protection reports		●	●	●					●
**10**	**Neighbourhood attachment**										
	Mobility	Individual and community mobility and stability.[1, 2]		●	●	●		●			●
	Perceptions of neighbourhood attachment	Perceptions of neighbourhood attachment or how connected they feel to the community.[2]		●	●	●		●			
**11**	**Child friendliness**										
	Perceptions of child friendliness	Perceptions of whether people in the community are perceived as being well disposed to children in public places, and whether the community is perceived as a "good" place to raise children.[2]		●	●	●		●			

	Community domain and sub-domain/s	Description	Policy document[2]	Stakeholder interview[2]	Parent focus group[2]	Practitioner focus group[2]	Service survey[2]	Community survey[2]	GIS & park audit[1]	Service information[1]	Community demographics[1]
**SOCIODEMOGRAPHIC DOMAIN**											
12	**Community Sociodemographic Status**	As defined by the Australian Bureau of Statistics (ABS) Socio-economic Index for Areas (SEIFA) Index of Relative Advantage and Disadvantage (IRSD)[1, 2]		●	●	●					●
13	**Community demographics**	Includes: Age profile, education, employment, ethnic and cultural diversity, household types, housing affordability, income[1, 2]		●	●	●		●			●
**SERVICE DOMAIN**											
14	**Quality**										
	Accreditation	Accreditation and licensing.[1]							●	●	
	Perceptions of quality	Perceptions about the quality of service, quality of care, welcoming staff, and the physical condition of service. This refers to how "good" the service is perceived for children and families.[2]		●	●	●					
15	**Quantity**										
	Number of services	Objective counts of the number of services in the area.[1] Perceptions of number of services.[2]		●	●	●		●	●	●	
	Number per capita	Objective number of services per population.[1]							●	●	●
	Utilisation	Client use of the service.[2]		●	●	●		●		●	
16	**Access to services**										
	Opening hours	Opening hours of the service.[1, 2]		●	●	●				●	
	Cost	Cost of what clients/patients pay to use the service.[1, 2]		●	●	●				●	
	Capacity	Open to new clients/patients, number of vacancies.[1, 2]		●	●	●				●	
	Waiting lists	Whether people have to wait to access a service.[1, 2]		●	●	●				●	
17	**Coordination**										
	Co-locations	Whether the service is co-located with other services.[2]		●		●	●		●		
	Collaborations/ networks/ partnerships	Partnerships and collaborations at the service implementation level.[2]		●		●	●				

	Community domain and sub-domain/s	Description	Policy document[2]	Stakeholder interview[2]	Parent focus group[2]	Practitioner focus group[2]	Service survey[2]	Community survey[2]	GIS & park audit[1]	Service information[1]	Community demographics[1]
**GOVERNANCE DOMAIN**											
**18**	**Context and characteristics**										
	History	Historical factors and events including environmental events that impact on the current arrangements including agenda and priorities, partnerships and collaborations.[2]	●	●							
	Multi-level governance	Characteristics of governance groups and/or community governance practices, including practices for decision making. This refers to 'vertical' governance- i.e. between levels of organisations.[2]	●	●							
	Priorities, policies, and programs	Key policies or programs relating to children. Agenda and priorities that are currently (or recently) being pursued by policy makers, partnerships, and collaborations in the community. Includes mention of priorities not specifically about children.[2]	●	●							
**19**	**Macro/Meso policy environment (context)**										
	Role of federal and state government locally, involvement of portfolio staff locally	The involvement and incidence of Federal and State programs and requirements in the area.[2]	●	●							
	Policies supporting/ requiring governance coordination	Federal and State requirements for coordination of governance.[2]	●	●							
**20**	**Representation and demographic effects - local**										
	Citizen involvement in decision-making	Transparent/ accountable/ responsive structures that have the ability to reflect community-level interests and ensure everyone has the right to have a say. The way that involvement is facilitated by the community, including membership of organisations and decision making bodies. Also references to inclusion or exclusion of groups and/or individuals.[2]		●	●	●		●			

	Community domain and sub-domain/s	Description	Policy document[2]	Stakeholder interview[2]	Parent focus group[2]	Practitioner focus group[2]	Service survey[2]	Community survey[2]	GIS & park audit[1]	Service information[1]	Community demographics[1]
**21**	**Decision making and leadership - local**										
	Common agenda	How is the local agenda agreed upon and is there general agreement or high levels of conflict- specifically referring to partnerships and coordination.[2]	●	●							
	Data for decision making	Any reference to data or evidence used for the purpose of decision making for policy.[2]	●	●							
	Key leaders	Involves individuals and organisations that are making a particular contribution, have a role in decision-making.[2]	●	●	●	●					
	Resources, rules, roles, structures	Local arrangements for the coordination of decision making, policies and programs and their implementation.[2]	●	●							

[1]Objective measure; [2]Perception; Table copied from Goldfeld et al., 2017[(3)]

1.3.2 Community Investigations

Further reading: http://bmjopen.bmj.com/content/7/3/e014047

Goldfeld, S., Villanueva, K., Tanton, R., Katz, I., Brinkman, S., Woolcock, G., Giles-Corti, B. (2017). *Kids in Communities Study (KiCS) study protocol: a cross-sectional mixed-methods approach to measuring community-level factors influencing early child development in Australia.* BMJ Open, Vol. 7

a. Interviews

Semi-structured interviews were conducted with a range of key stakeholders relating to early childhood in the local community or municipality (e.g. managers of early years' services, local government and non-government staff involved in the early years, and school principals). Stakeholders were recruited through purposive and snowball sampling.[(22)] No further interviews were conducted when data saturation (i.e. no 'new' information obtained) was achieved. While the interview questions were primarily focused on the governance and service domains, open-ended questions about stakeholder perspectives on what they considered as positive and negative (challenges or difficulties) community factors for young children and families were asked. Interviews ranged from 35 mins-1.5 hours. We aimed to interview approximately 8-15 participants per cluster of local communities (i.e. AEDC 'community' or local government area). Participants provided written and verbal consent to participate and have the interview recorded, transcribed and analysed.

b. Focus groups

For each local community, two focus groups were undertaken, one with local service providers and one with parents of children aged 0-8 years living in the local community. We aimed to have at least 4 participants in each focus group. Service providers and parents were recruited through stakeholder engagement. Parents were also recruited through distributing flyers through local organisations, and/or snowball sampling. Parents were reimbursed with a $AUD 25 Coles/Myer gift card for their time and participation. Interviews using focus group questions were completed where a focus group could not be organised. Focus groups were held for 45-90 minutes with open-ended questions about each domain.

For the focus groups and interviews, best efforts to 'control' for perceptions of the pre-defined local community geographic boundary were implemented (a map of the boundary was shown to participants).

c. Policy documents

Policy documents relating to the early years were sourced for analysis to provide contextualization for each local community. Examples of relevant policy documents are local government documents such as municipal early years' plans, annual reports focused on early childhood and infrastructure reports. As governance structures are likely to exist across a municipality, approximately 10-12 policy documents were collected per municipality or cluster of local communities. Only those that had a particular reference to local communities of interest were explored.

d. Community surveys

Between March and June 2016, a survey about perceptions and attitudes about the community was distributed to 1000 residents per local community (i.e. 25,000 in total), through random sampling of residents (aged 18 years or older) registered in the Australian Electoral Commission (AEC) database. This sampling method provides coverage because in Australia, it is compulsory to vote if you are over the age of 18 years old. The survey was distributed online, phone and/or hardcopy using a three-staged approach, which involves multiple waves of survey distribution (pre-notification, survey, and reminder). Survey questions included a combination of validated items from existing surveys and derived items where existing items were non-existent.(3) Prior to the main data collection, the survey underwent test-retest reliability (two weeks apart) with a small convenience sample, to ensure face validity (content and structure).

e. ABS Census data

The SES measures had to be available for the AEDC local communities (suburbs, SA2 level), so needed to be available on the Australian Census. The data used in this analysis were taken from the 2011 Australian Census of Population and Housing (the Census). Data were extracted for each community using the *TableBuilder* application provided by the ABS.(23) It can also be extracted using the ABS General/Basic Community Profile.(24) For all our Census data, the data is for the whole suburb, and not only for people with young children. This is consistent with our theory that it is the whole suburb that has an impact on child development, rather than just the socio-economic characteristics of families with children. Public housing and retirement villages in a suburb all contribute to the overall feeling in the suburb, so are important contributors to include in our measures of SES. The exception, however, was for the proportion of residents working as managers or professionals and with a university degree, where data were limited for residents aged 25 to 54 years only. This was done to minimise the effect of demographic variation within communities.(25)

f. GIS data

GIS software (ESRI ArcGIS v10.3.1)(26) was used to create built environment measures (e.g. presence of, and distance to selected destinations, and neighbourhood walkability) using existing spatial datasets where possible. There are many types of built environment measures, and each type requires different data and ways to calculate it. Examples of existing information sources used include destination data from the Raising Children Network,(27) the Australian Urban Research and Infrastructure Network (AURIN)(28) and local government websites. As there were a number of regional local communities within the study, most of the datasets had to be manually supplemented by 'cross-checking' or validating with data from local government websites to ensure more accurate and comprehensive data for that region. Built environment measures for the 'local community' (ABS Suburb, SA2 level) were computed.

1.3.3 Data cleaning and preparation

All focus groups and interviews were recorded and transcribed for analysis. Transcripts were imported into QSR International's NVivo v11, a software program designed to assist with organising and coding qualitative data.[29] Content analysis through a deductive approach was used according to a predetermined coding framework relating to all five domains that were developed by the research team. Seven researchers coded the data. Information that did not 'fit' within the existing codes but seemed to be important to the study was coded as 'other useful information'. To ensure analytical rigour and consensus, issues were consolidated through regular team coding discussions and updating shared documentation to ensure consistency; such approaches have been used in previous studies.[30, 31] Quantitative data were cleaned (e.g. identify missing data) and data reduction strategies (e.g. composite variables and scales) were used to identify a set of quantitative variables for further exploration.

1.4 Data analysis - Developing the FCFs

The KiCS data analysis approach involved two stages (see **Figure 3** in Executive Summary). **Stage 1** involved exploring the factors that differentiated why some local communities had better AEDC outcomes than their neighbouring local community, despite both being socioeconomically disadvantaged. **Stage 2** involved exploring the qualitative factors only, and tried to understand which factors were consistently important for the majority of the 25 local communities in the study. This section describes how KiCS identified and developed the draft list of FCFs for ECD based on our data.

1.4.1 Stage 1 of data analysis: Differentiating factors

Stage 1 analysis focused on data for 14 local communities (suburbs) in seven local government areas (AEDC communities) as matched-disadvantaged pairs. That is, seven pairs of neighbouring on- and off-diagonal local communities with the same disadvantaged SES. The preliminary findings focus on comparing on- and off-diagonal local communities to elicit consistent community-level factors that differentiate diagonality and promote better ECD outcomes.

Matched disadvantaged (low SES) pair:

Two disadvantaged local communities; one doing well (Off-diagonal positive) and one doing poorly (On-diagonal disadvantaged)

a. Qualitative data analysis

Differences were explored between disadvantaged local communities doing better than expected on ECD (off-diagonal) and disadvantaged local communities performing as expected on ECD (on-diagonal). The purpose of the qualitative analysis was to identify emerging themes or factors related to ECD. While quantitative and qualitative data were analysed concurrently, the qualitative data were used to help inform which factors should be further explored with quantitative data. Analysis of common themes differentiating on- and off-diagonal local communities outlined the significant or important factors in the community and how participants explained the difference between the on- and off-diagonal communities. Strong themes or factors were identified both within and across community pairs. Participants indicated in their own words what they felt

were the factors that support children's development in their community. A particular theme/factor was considered 'strong' if: 1) participants mentioned a particular factor without prompting or probing, or they indicated that a factor or theme as important; 2) different groups of participants (parents, professionals, policy makers) identify a common theme as being important; and/or 3) several participants number of participants indicated that a particular factor was important.

b. Quantitative data analysis

Similar to the qualitative analysis, strong factors differentiating an on- and off-diagonal local community were identified. For quantitative data, descriptives were calculated. Descriptives were calculated using Microsoft (MS) Excel and Stata v14,[(32)] for each local community and any differences within matched community pairs were identified. There was no further scope to explore associations between quantitative data and the AEDC (for example, using regression models) due to the small number of communities in our sample (i.e. 25 local communities, and only 14 local communities clustered within seven matched-disadvantaged pairs).

c. What is a 'difference' between on- and off-diagonals?

Comparing on- and off-diagonal local communities involved assessing 'differences' between these communities (see **Table 4**). What we considered a 'difference' depended on the type of data collected.

Table 4. Assessment of 'differences' between on- and off-diagonals

Data type		Assessment of 'difference' between on- and off-diagonals
1	Australian Bureau of Statistics Census data (2011)	As the data used were from a Census, the data are accurate for small areas and there are no confidence intervals; we considered a 'large' change as a 5% difference between local communities.
2	Geographic Information Systems (GIS) and park audit data used to measure the built environment of the community	An absolute value for each built environment feature was reported, thus it was not possible to conduct any meaningful statistical analyses to compare values within each matched pair. The magnitude of the 'difference' between Off+ and OnDis local communities within each matched pair was assessed by calculating the mean and standard deviation (SD) for each built environment measure across the 25 local communities in the overall KiCS study, and assessing whether the absolute value was less or more than one SD from the mean.
3	Qualitative data (e.g. focus groups and interviews)	Emerging themes from the data were identified based on:<br>Participant's views: participants spontaneously mention a particular factor (i.e. without prompting or probing) or they indicate that a factor or theme is important.<br>Triangulation: different groups of participants (parents, professionals, policy makers) identify a common theme as being important.<br>Numbers: large numbers of participants indicate that a particular factor is important then this is an indication.<br>Qualitative analysis is NOT about identifying numbers of people who said "x" vs "y" about a particular issue. Quantifying the responses may be interesting but is doubly problematic from an analysis point of view.
4	Survey data (e.g. community surveys)	Statistical differences (p-values ≤0.05) and confidence intervals (e.g. t-tests and chi-squared tests of significant differences between local communities).

d. Comparing on- and off-diagonals

Comparing on- and off- diagonal local communities begins to answer the question about *why* some local communities were doing better for children's ECD despite still being disadvantaged (or vice versa). A directional hypothesis or theory for each theme or factor was identified based on previous literature.

Example of directional hypothesis:

Despite both being economically disadvantaged, there are more parks in the local community doing well, compared with the local community doing poorly

In brief, the purpose of the analysis was to explore:

- Does the qualitative finding/s support or deny our hypothesis?
- Does the quantitative finding/s support or deny our hypothesis?
- Do the qualitative and quantitative finding/s match?
- Are there any new hypotheses being generated from the data?

In doing so, a three-staged approach to both qualitative and quantitative analysis was undertaken (see **Figure 4**):

- **Phase 1:** *Within* community pairs: Does the theme/factor differentiate between on- and off-diagonal local communities?
- **Phase 2:** *Across* community pairs: Is there a consistent pattern emerging across community pairs? For both qualitative and quantitative measures, a 'consistent pattern' was whether the same finding appeared in at least four or more community pairs.
- **Phase 3:** *Overall triangulation* of qualitative and quantitative data: Do the qualitative and quantitative findings match? Where possible, a qualitative and (equivalent or proxy) quantitative measure were aligned and linked to the KiCS' domains and sub-domains. Both qualitative and corresponding quantitative measures were assessed for conceptual alignment (i.e. are they measuring the same construct?).

Example of conceptual alignment for triangulation:

A qualitative measure of park availability would be ***more perceived parks****. The best matched quantitative measure would be:* ***a greater number of parks***

The aim of the triangulation was to provide more support for any consistent community-level factors associated with on-and off-diagonal local communities. The triangulation process (or convergent validation) enabled a broader and deeper exploration of domain-specific community factors in on- and off-diagonal local communities.[(33)] Fielding[(33)] emphasises that both types of data are essential and the benefit of mixed-methods research is "rather than mixing because there is something intrinsic or distinctive about quantitative data or qualitative data, we mix so as to integrate two fundamental ways of thinking about social phenomena" (pp 125-126).[(33)] As such, integrating qualitative and quantitative data facilitates the conceptualisation of potential mechanisms with rich contextual understanding to explain complex interactions relating to community-level factors that may influence ECD. To make it easier to make sense of the findings, results were visualised in a 'heat map' matrix. To illustrate the 'heat map' matrix, an example is in **Figure 4**.

Triangulation *of qualitative and quantitative findings provides stronger support for the community-level factor associated with ECD*

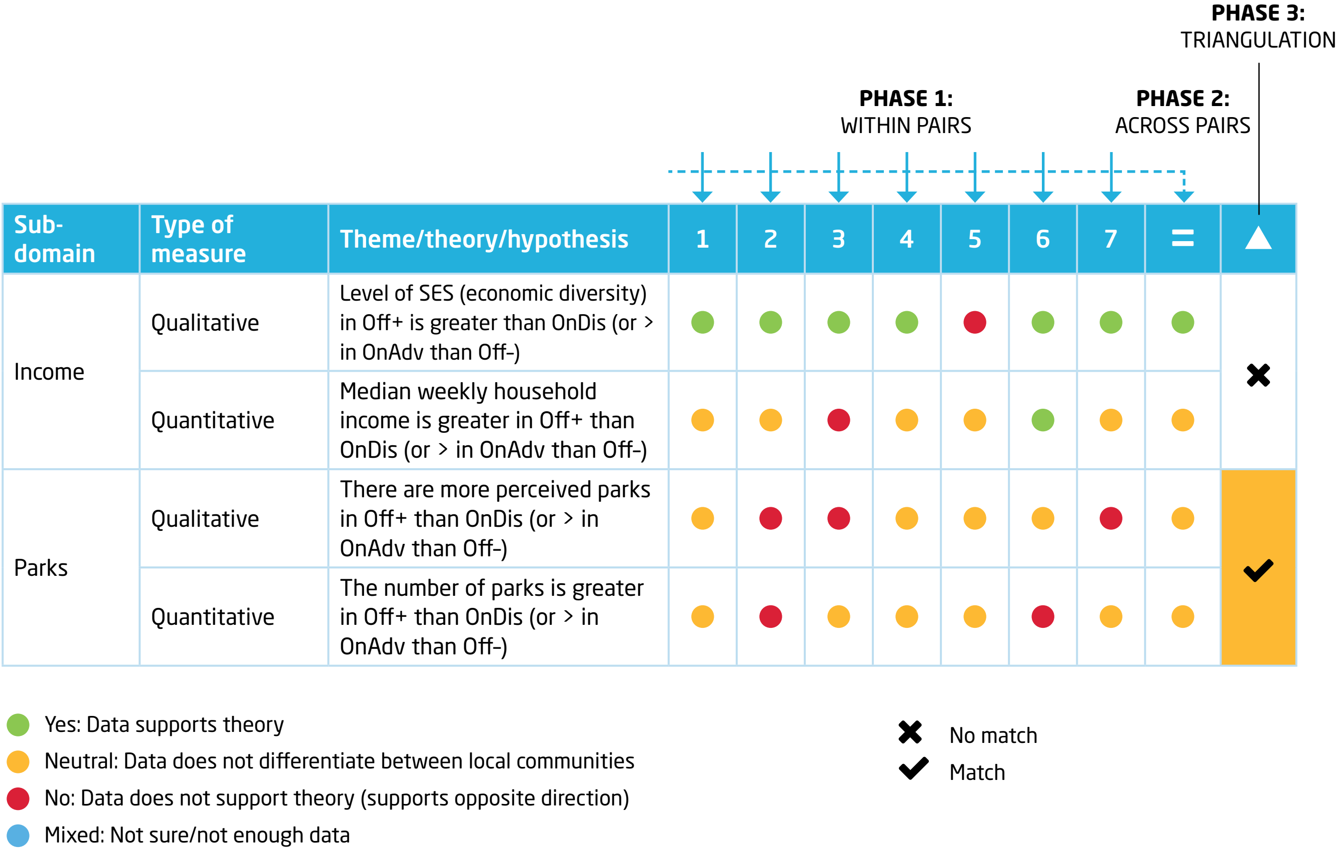

Figure 4: Example of a heat map matrix showing results within and across matched-disadvantaged community pairs (stage 1 data analysis)

1.4.2 Stage 2 of data analysis: Important factors

The second stage of analysis prioritised the *qualitative data only* and explored factors that communities perceived as important for ECD, regardless of whether their community was doing better, worse or as expected on the AEDC relative to their SES. We analysed common themes that appeared important in the community for early childhood. Strong themes or factors were identified both within and across all 25 local communities in KiCS. Participants indicated in their own words what they felt were the factors that support children's development in their community. A particular theme/factor was considered 'strong' if:

1. Participants mentioned a particular factor without prompting or probing, or they indicated that a factor or theme as important;
2. Different groups of participants (parents, professionals, policy makers) identify a common theme as being important; and/or
3. Several participants indicated that a particular factor was important.

1.4.3 Summary of FCF development

To recap, the results of the KiCS community investigations and data analysis have informed the development of a promising (draft) set of FCFs.

a. Differentiating FCFs from Stage 1

Community factors measured qualitatively and/or quantitatively that have *consistently differentiated* neighbouring on- and off-diagonal local communities in *at least four* of the seven matched-disadvantaged pairs. **Further considerations:**

- Ideally, a differentiating FCF can be measured both qualitatively (e.g. people's perceptions) and quantitatively (e.g. numeric measurement). This triangulation allows perceptions to be considered alongside objective measures. In KiCS, not all measures were identified as having a 'matching' qualitative or quantitative measure thus these measures were not eligible for triangulation (**Section 1.4.1 Comparing on and off-diagonals**) but still included as a FCF (if they met the Stage 1 eligibility).
- Some of the quantitative differentiating FCFs were suitable for indicator development. Given the limitations of our data and approach to developing indicators, the set of indicators we proposed are described further in Part 2.

b. Important FCFs from Stage 2:

Community factors that were *consistently important* in at least 16 of the 25 local communities were included in the suite of FCFs. However, they were not eligible for further indicator development at this stage due to the qualitative nature of the analysis (**Section 2.3** has details on indicator development eligibility). Only the 'differentiating' FCFs (Stage 1) are presented in the supplementary **KiCS FCF Manual** as the *most promising subset* of draft FCFs for local measurement because qualitative and/or quantitative testing has occurred (rather than only qualitative in Stage 2). Community factors that are consistently important for communities are not currently presented in the **KiCS FCF Manual**, but are summarised in this report.

The thresholds chosen for 'consistently differentiating' (i.e. four or more of the matched-disadvantaged community pairs); or 'consistently important' (i.e. 16 of the 25 local communities), were selected based on qualitative research expert opinion, as there is no 'gold standard' or evidence base as precedence. While a pattern of a majority (i.e. four or more pairs out of seven) was deemed an appropriate signal of a pattern of differentiating factors, a larger proportion (16 or more out of 25 local communities) for the important factors was required to be considered a finding. We used this definition consistently throughout the study.

PART 2 **KiCS findings**

2.1 **Field work information**

While KiCS aimed to conduct at least one parent and practitioner focus group per local community, at least 8 interviews per local government area (community), and obtain a 30% response rate for each local community, these 'targets' varied for each state and community. Despite best efforts, recruitment challenges varied by context, and ranged from ethics rejections (e.g. primary schools) to difficulties in accessing local parents and service providers. In cases where no parent focus groups were obtained, interviews using parent focus group questions with parents were held where possible. A summary of data collection (field work) is in **Table 5** is below:

Table 5. Summary of field work in local communities

Summary of KiCS fieldwork	Key stakeholder interviews	Focus groups with local service providers	Focus groups with parents of children aged 0-8 years	Community survey responses	Service survey responses with local service providers
**VIC 1**					
VIC-1-1 'Better than expected'		1 (14x participants)	2# (14x participants)	184	15
VIC-1-2 'Disadvantaged as expected'		1 (9x participants)	1 (6x participants)	170	13
VIC-1-3 'Advantaged, As expected'		1 (7x participants)	1 (4x participants)	212	11
**Total VIC-1**	**21^**	**3**	**4**	**566**	**20**
**VIC 2**					
VIC-2-1 'Worse than expected'		1 (10x participants)	1 (6x participants)	129	3
VIC-2-2 'Disadvantaged as expected'		1 (14x participants)	2 (22x participants)	172	12
VIC-2-3 'Advantaged, As expected'		1 (6x participants)	2 (17x participants)	195	8
**Total VIC-2**	**15**	**3**	**5**	**496**	**18**
**NSW 1**					
NSW-1-1 'Better than expected'		3 (17x participants)	2 (7x participants)	123	3
NSW-1-2 'Disadvantaged as expected'		1 (7x participants)	1 (3x participants)	120	8
**Total NSW-1**	**10**	**4**	**3**	**243**	**9**
**NSW 2**					
NSW-2-1 'Better than expected'		3 (19x participants)	1 (9x participants)	128	5
NSW-2-2 'Disadvantaged as expected'		1 (11x participants)	1 (6x participants)	136	4
**Total NSW-2**	**10**	**4**	**1**	**264**	**6**

Summary of KiCS fieldwork	Key stakeholder interviews	Focus groups with local service providers	Focus groups with parents of children aged 0-8 years	Community survey responses	Service survey responses with local service providers
**NSW 3**					
NSW-3-1 'Better than expected'		1 (5x participants)	2@ (11x participants)	141	6
NSW-3-2 'Disadvantaged as expected'		1 (7x participants)	2@ (9x participants)	140	5
**Total NSW-3**	**15**	**2**	**3@**	**281**	**9**
**SA 1**					
SA-1-1 'Worse than expected'		1* (5x participants)	1 (7x participants)	180	3
SA1-2 'Advantaged as expected'			1 (3x participants)	202	5
**Total SA-1**	**14**	**1***	**2**	**382**	**5**
**SA 2**					
SA-2-1 'Better than expected'		2 (6x participants)	1 (4x participants)	185	5
SA-2-2 'Advantaged as expected'		1 (7x participants)	1 (5x participants)	229	4
**Total SA-2**	**14^**	**3**	**2**	**414**	**8**
**QLD 1**					
QLD-1-1 'Better than expected'		1 (5x participants)	0	140	3
QLD-1-2 'Disadvantaged as expected'		1(4x participants)	1 (5x participants)	120	3
**Total QLD-1**	**12^**	**2**	**1**	**260**	**5**
**QLD 2**					
QLD-2-1 'Better than expected'		1* (7x participants)	1 (4x participants)	172	3
QLD-2-2 'Disadvantaged as expected'			1 (3x participants)	172	3
**Total QLD-2**	**10**	**1***	**2**	**344**	**3**
**QLD 3**					
QLD-3-1 'Worse than expected'		1* (8x participants)	1 (3x participants)	116	4
QLD-3-2 'Disadvantaged as expected'			1 (6x participants)	104	4
**Total QLD-3**	**10**	**1***	**2**	**220**	**4**
**ACT 1**					
ACT-1-1 'Better than expected'		See below	0	241	0
ACT-1-2 Disadvantaged as expected'		See below	0	203	0
ACT-1-3 'Worse than expected'		See below	0	230	0
**Total ACT-1**	**9^**	**1 (4x participants)**	**2 (interviews with parents instead of focus groups)**	**674**	**0**

Summary of KiCS fieldwork	Key stakeholder interviews	Focus groups with local service providers	Focus groups with parents of children aged 0-8 years	Community survey responses	Service survey responses with local service providers
	Key stakeholder interviews	Focus groups with local service providers	Focus groups with parents of children aged 0-8 years	Community survey responses	Service survey responses with local service providers
**TOTAL**	**146**	**25**	**26**	**4144**	**87**

e.g. VIC-1-1 = [State/territory] - Community ID - Local community ID (communities are not named in this report)

Note: 2x parent focus groups are pilot focus groups, 1 in VIC and 1 in NSW; 2x interviews for NSW-3 are also for NSW-1 and NSW-2; ^More than one interviewee in some interviews; #One focus group is a pilot focus group; @One focus group is a pilot focus group that covered both local communities; *Focus group covered both local communities

2.2 The differentiating FCFs

What are the factors that consistently point to disadvantaged communities doing well on ECD?

- **Table 6** describes the list of differentiating FCFs and what KiCS found.
- The results were visualised in a 'heat map' matrix provided in **Appendix 7.3**.
- A detailed discussion for each differentiating FCF is in **Part 3. The Differentiating FCFs**.

Table 6. Differentiating Foundational Community Factors – what KiCS found

Differentiating Foundational Community Factor – What KiCS found differentiates disadvantaged communities doing well or poorly on ECD		
1	Income	Median household income[1] and degree of economic diversity[2] is greater in disadvantaged areas doing well on ECD
2	Highest level of schooling	There is a higher proportion of the population that have completed Year 12 or equivalent[1] in disadvantaged areas doing well on ECD
3	Gentrification	Relatively higher income (but still disadvantaged) families are moving into disadvantaged areas doing well on ECD, resulting in the displacement of more disadvantaged groups[2]
4	Housing affordability	Housing is perceived as more affordable in disadvantaged areas doing well on ECD[2]
5	Housing tenure (stability)	There is a lower proportion of renters compared to private home owners in disadvantaged areas doing well[1]
6	Public housing	There is a lower proportion of public renters[1] and less perceived presence of public housing[2] in disadvantaged areas doing well on ECD
7	Housing density	There is a lower proportion of high rise (three or more storeys)[1] and perceived fewer high rise density dwellings (vs low rise housing developments)[2] in disadvantaged areas doing well on ECD
8	Stigma	Negative reputation of a local community[2] is less in disadvantaged areas doing well on ECD[2]
9	Primary school reputation	Primary school reputation was more positive in disadvantaged areas doing well on ECD[2]
10	Perceived ECEC availability	There was more perceived ECEC availability in disadvantaged areas doing well on ECD[2]
11	Perceived crime	There was less perceived crime in disadvantaged areas doing well on ECD[2]
12	Historical events	Leaders respond to events in ways that bring local community members together to create a shared storyline and/or engage in activities of citizenship[2] is greater in disadvantaged areas doing well
13	Local decision-making	As a result of local decision-making, 'novel approaches' or locally tailored initiatives or solutions (including any with a focus on social capital) have been developed in the community doing well[2]

[1]Quantitative; [2]Qualitative; area=local community

2.3 The important FCFs

What are the factors that are consistently important for ECD across local communities (regardless of diagonality)?

- **Table 7** describes the list of important FCFs. Three encouraging important FCFs have appeared, but the analysis is incomplete.
- **Table 10** in **Part 4** provides examples from KiCS data and how this relates to ECD.
- A summary of the important FCFs is outlined in **Part 4. Important FCFs**.

Table 7. *Important* **Foundational Community Factors – what KiCS found**

Important Foundational Community Factor – What KiCS found is important for communities		
1	Physical access to services	Reported instances of ability to get to services
2	Walkability	Perceived walkability to facilities and services was seen as important for physical access
3	Public transport availability	Perceived presence of/access to public transport was seen as important for easy access within the suburb
4	Traffic exposure	Being away from traffic within the suburb is an important factor for children being safe
5	Public open space – availability and quality	Having parks in the suburb was seen as important for young children and families. Having good quality parks was seen as important for use, play, and social interaction
6	Facilities – availability and diversity	Having a range of family-friendly destinations and activities is important for young families and children
7	ECEC cost	Perceived affordability of ECEC is considered important and affects use
8	Leadership	The presence of local champions, leaders and boundary spanners driving local governance
**Encouraging Important FCF – Analysis incomplete**		
9	Service Co-ordination	Co-ordination of services in a local community
10	Sense of community	Reported strong neighbourhood attachment or sense of belonging and pride in being connected to a local community
11	Natural environments	Natural spaces are seen as important for young families

All Important FCFs are qualitative

While the focus of KiCS was to identify modifiable (open to change) factors, there are also general context FCFs that appeared to be important for ECD. General context FCFs can potentially be modified through complex policy and system changes that go beyond the local community. These general factors are not usually direct policy targets but are important information for understanding the social landscape,[(1)] and can also help with local planning. An example is the proportion of families within the suburb.

2.4 Measuring the FCFs…towards better 'indicators'?

While KiCS collected rich localised information on the FCFs, there is much interest in measuring the *progress* of societies worldwide through the use of indicators. In this way, indicators are measureable over time. While there are many definitions of what an indicator is, the consensus is that an indicator provides a **summary indication of the condition or problem, and permits the observation of progress or change**.(2) Indicators are distinct from statistics and primary data in that they represent more than the data on which they are based.(9) For example, an unemployment rate is an indicator; but it is made up of labour force statistics on the number of people unemployed, and the number of people in the labour force. As such, indicators are usually quantitative, rather than qualitative. There were few measures eligible for indicator development in KiCS as many of the differentiating factors were qualitative measures, and there were limitations associated with the quantitative measures used in the study (see **Limitations section 5.2**). Some of the qualitative FCFs may eventually be developed into 'indicators', but further investigation is needed in KiCS before we can reach this stage.

For the time being, the quantitative FCFs that *consistently differentiated* on- and off-diagonality ('as expected' vs. 'better than expected') across at least four of the seven matched-disadvantaged pairs were assessed against a set of indicator selection criteria (see **Table 8**) derived from the OECD.(2) The criteria were used as a tool to evaluate the proposed indicators to ensure they are relevant and measurable. While the qualitative differentiating FCFs were not eligible for indicator development, we were still interested in exploring them against some of the criteria.

Table 8. OECD selection criteria for indicator development

OECD Selection Criteria		Description
1	Valid and meaningful to the community	An indicator should adequately reflect the phenomenon it is intended to measure and should be appropriate to the needs of the user
2	Sensitive and specific to the underlying phenomenon	Sensitivity relates to how significantly an indicator varies according to changes in the underlying phenomenon
3	Grounded in research	Awareness of the key influences and factors affecting outcomes
4	Statistically sound	Indicator measurement needs to be methodologically sound and fit for the purpose to which it is being applied
5	Intelligible and easily interpreted	Indicators should be sufficiently simple to be interpreted in practice and intuitive in the sense that it is obvious what the indicator is measuring
6	Relate where appropriate to other indicators	A single indicator often tends to show part of a phenomenon and is best interpreted alongside other similar indicators
7	Allow international comparison	Indicators need to reflect Australian-specific goals, but where possible should also be consistent with those used in international indicator programmes so that comparisons can be made
8	Ability to be disaggregated over time	Indicators should be able to be broken down into population sub-groups or areas of particular interest, such as ethnic groups or regional areas
9	Consistency over time	The usefulness of the indicators is directly related to the ability to track trends over time, so as far as possible indicators should be consistent
10	Timeliness	There should be minimal time lag between the collection and reporting of data to ensure that indicators are reporting current rather than historical information
11	Linked to policy or emerging issues	Indicators should be selected to reflect important issues as closely as possible. Where there is an emerging issue, indicators should be developed to monitor it
12	Compel interest and excite	The indicator should resonate with the intended audience

An individual indicator may not meet all of the selection criteria.(2) The process of selecting the indicators involved judgement about which criteria were the most important to meet. At the very least, the indicator should be valid and meaningful (relevant), linked to the outcome, and measurable. The KiCS research team thoroughly assessed each differentiating FCF against each criteria (see **Table 9**), with input from key stakeholders (see **Acknowledgements**).

Due to the limitations of the quantitative measures and methods of KiCS, future research should explore stronger quantitative models of community factors and ECD, and subsequently develop more robust evidence-based quantitative indicators for ECD. Until further testing is carried out, the indicators of Income, Highest Level of Schooling, Housing tenure (stability), and Public housing are more 'signals' of areas of focus, rather than robust community indicators of ECD. The description for each of these indicators are discussed in more detail in this report, and its measurement and interpretation should be read in conjunction with the **KiCS FCF Manual**.

Table 9. Assessment of KiCS 'indicators' against selection criteria

		OECD Selection criteria for indicators											
Draft indicators		Valid and meaningful to the community	Sensitive and specific to the underlying phenomenon	Grounded in research /theory (expert endorsement)	Statistically sound	Intelligible and easily interpreted	Relate where appropriate to other indicators	Allow international comparison	Ability to be disaggregated over time	Consistency over time (3 years)^	Timeliness	Linked to policy or emerging issues	Compel interest and excite
1	Income*[1,2]	✓	?	✓	✓	✓[2]	✓	✓	✓	✓	✓	✓	✓
2	Highest level of schooling*[2]	✓	?	✓	✓	✓	✓	✓	✓	✓	✓	✓	✓
3	Gentrification[1]	✓	?	✓	N/A	✗	✓	✗	✗	N/A	✗	✓	✓
4	Housing affordability[1]	✓	?	✓	N/A	✗	✓	✗	✓	✗	✗	✓	✓
5	Housing tenure (stability) (renters vs. private owners)*[2]	✓	?	✓	✓	✓	✓	✗	✓	✓	✓	✓	✓
6	Public housing*[1, 2]	✓	?	✓	✓	✓[2]	✓	✓	✓	✓	✓	✓	✓
7	Housing density[1,2]	✓	?	✓	✓	✗[2]	✓	✓	✓	✓	✓	✓	✓
8	Stigma[1]	✓	?	✓	N/A	✗	✓	✗	✗	✗	✓	✓	✓
9	Perceived primary school reputation[1]	✓	✓	✓	N/A	✗	✓	✗	✗	✗	✓	✓	✓
10	Perceived ECEC availability[1]	✓	✓	✓	N/A	✗	✓	✓	✗	✗	✓	✓	✓
11	Perceived crime[1]	✓	✓	✓	N/A	✗	✓	✗	✗	✗	✗	✓	✓
12	Historical events[1]	✓	?	✓	N/A	✗	✓	✗	✗	✓	✓	✓	✓
13	Local decision-making[1]	✓	?	✓	N/A	✗	✓	✗	✗	✓	✓	✓	✓

[1]Qualitative; [2]Quantitative; N/A: Not applicable; ^3 years given Australian Early Development Census data collections every three years

? Don't know/cannot assess how this varies according to changes in ECD

*possible draft indicator for ECD; The above indicators may be available and used elsewhere (e.g. ABS Census). Housing density not considered a draft indicator for ECD because it is not specific to public housing

2.5 Moving beyond indicators…the importance of mixed methods

The quantitative data provides the 'what' and the scale of the problem; and the qualitative data provides the 'why' and how to improve it.

A summary of the FCFs for ECD and methods KiCS used to measure them are listed in **Appendix 7.2**. Some were associated with ECD outcomes based on our analysis approach (e.g. Stage 1 and 2); these were recommended for qualitative and/or quantitative measurement, and further described the **KiCS FCF Manual**. While not all FCFs are currently recommended for quantitative or indicator measurement, it does not mean that these more 'qualitative' factors should not be better understood by communities. What has emerged from KiCS is that the qualitative findings have provided insight into the factors and pathways that might influence ECD. Given there are few robust large-scale studies that explore community-level effects on ECD, the qualitative findings shed light on what FCFs may be explored further, and why. With further research, quantitative measures and indicators could be developed based on our qualitative findings.

While quantitative indicators may be useful for measuring the progress of communities towards better ECD outcomes, having a complementary qualitative measurement of the factor is equally important. In this way, quantitative and qualitative measures can be used alongside each other to capture a better understanding of what elements are important for young children in the community. To illustrate, FCFs can be developed into quantitative indicators to capture a 'barometer' reading of a community. However, equally important to understand is the 'what lies beneath' an indicator (e.g. underlying factors to help better understand 'why' the indicator is important); such factors can be qualitatively captured (e.g. through interviews or focus groups).

As an example, walkability (i.e. walkable environments that facilitate ease of pedestrian travel) is a FCF. It can lend itself to being an indicator measured either objectively (e.g. by Geographic Information Systems) or subjectively (e.g. by surveys). Qualitative methods can then be used to better understand if and why this indicator is an important priority area for the community, and what can be done locally to improve it. So the quantitative data provides the 'what' and the scale of the problem; and the qualitative data provides the 'why' and how to improve it. Capturing this information may allow communities to: 1) identify their short, medium, and long term priorities; and 2) provide insight into how they can address the indicator at a local level.

PART 3 Differentiating FCFs

The differentiating FCFs are the community-level factors that consistently point to why local communities had better ECD outcomes than their neighbouring local community, despite both being socioeconomically disadvantaged. We have suggested these FCFs as the *most* promising subset of the FCFs (for now), and suggested ways to measure and use them are described further in the **KiCS FCF Manual**. The interpretation and limitations of the differentiating FCFs should be read in conjunction with the manual.

The 13 differentiating FCFs provide some key focus areas for communities to consider for possible local place-based initiatives, despite the caveats and interdependencies with other FCFs. The differentiating FCFs are largely related to the KiCS SES, social and service domains, and there are fewer physical and governance factors. Possible reasons are highlighted in the overall **Limitations**, which include the measurement of the physical domain factors, limited ability to develop robust quantitative models with ECD, and issues around the geographic scope of the measurement of these particular domains. There are four potential draft indicators for ECD; 1) income; 2) highest level of schooling; 3) housing tenure (stability); and 4) public housing.

3.1 Income

3.1.1 Relationship with ECD

***Income** provides an indication of the SES of residents living in the suburb. It captures the level of income within the community and may indicate more affluent neighbours. Income also points to economic diversity, the mix of higher and lower income residents in the suburb.*

Markers of neighbourhood socio-economic status or composition is the strongest evidence available for 'neighbourhood effects' on ECD.[34] For children, socio-economic status (SES) impacts wellbeing at multiple levels, including the family and neighbourhood.[35] Its effects are moderated by individual and family characteristics, and external support systems (e.g. programs for low-income families). SES can be measured in many ways, including some quantification of income, and parental education.

It is important to consider community-level SES because there is evidence showing associations with children's health, achievement, and behavioural outcomes even when individual-level income and education are controlled.[5, 36] When poverty and other forms of disadvantage is geographically concentrated, negative impacts on child health and wellbeing are exacerbated.[5] Disadvantaged neighbourhoods may place children at increased risk for a variety of stressful events, including exposures to violence, crime, hazards as well as less access to recreational and institutional resources.

3.1.2 What KiCS found

In KiCS, local communities where children were doing better than expected had more income diversity than local communities where children were not doing well, despite being socio-economically similar. Income provides an indication of the SES of residents living in the suburb in terms of material quality of life. KiCS measured income qualitatively and quantitatively in a number of ways but found that the qualitative and quantitative measures below differentiated a disadvantaged local community doing well, from one that was doing poorly in ECD. The quantitative measure may be an indicator.

INCOME	What are we measuring?	How we measured it
Qualitative	Level of higher income, more affluent residents. Economic diversity, mix of higher and lower income residents	Interviews and focus groups
Quantitative	Median weekly household income	Australian Bureau of Statistics data

The qualitative data provides a more nuanced understanding of income within the suburb, while the quantitative measure provides an overall snapshot of the suburb's context.

- It focuses on the amount or proportion of higher income earners within the suburb, a proxy for the presence or absence of affluent neighbours.
- It also captures economic diversity, or a mix of higher and lower incomes in the area.

> ***"It's a wide mix of families. As I said, we have some very affluent families and then some very low socioeconomic families there as well. It's very extreme in some respects. Probably on the whole, the majority would be on that lower end of the wealth side of things, on the poverty side"*** (INT131)

It is unsurprising that income was consistently related to why some disadvantaged communities had better ECD outcomes than their neighbouring disadvantaged communities. The presence or absence of affluent or middle-class areas in a neighbourhood is often found to influence children's development outcomes.[4] For example, previous research has found that young children's IQs were higher in neighbourhoods with greater concentrations of affluent neighbours, while having low-income neighbours appears to increase the incidence of externalizing behaviour problems.[37] However, the deconcentration of poverty involves mixing households with different incomes, and this appears to have unintended consequences for different community groups.

3.1.3 Limitations with measurement and use

Income is difficult to change at the community-level or beyond (e.g. national or state policies). It also appears to be closely related to many of the other FCFs in the list (e.g. stigma, public housing, gentrification). While 'income-mixing' presents opportunities for affluent neighbourhoods to be involved in collective socialisation, previous studies recommend these processes need to be facilitated with care. For example, gentrification appears to be the most prevalent means of deconcentrating poverty in recent years,[38] (see **Gentrification section 3.3**) but we are not necessarily advising that communities gentrify. Rather, for disadvantaged communities that may have 'higher' income compared with other disadvantaged communities, it may be that local efforts be directed at limiting potential isolation of more disadvantaged members and groups of the community. Studies of gentrified communities have found that social interactions appear to be infrequent between say, renters vs. owners, middle vs. lower-income groups.[39]

Local efforts can be directed at limiting potential isolation of more disadvantaged members and groups of the community

In KiCS communities, a number of barriers were identified for low income families that may not only hinder access to basic necessities but also social processes and access to important services:

- Inability to pay for sports leading to social exclusion
- Lack of money for food which affects school attendance (parents being embarrassed about it and would rather their child stay at home until they have food to bring to school)
- Lack of disposable income to get to places and activities

Potential opportunities for using the FCF of income at the community-level include:

- Creating supportive settings for social interaction may be one way to build relationships between different groups.
- Free events in different settings (e.g. child-care, parks, schools) may be coordinated around specific goals of 'getting to know your neighbours' and encouragement and support of harder-to-reach groups to attend. While a sense of community did not appear to consistently differentiate diagonality, it may still be important to a community, and for different sub-groups within a community.

3.2 Highest level of schooling

3.2.1 Relationship with ECD

Families exist in relation to other people, resources, and opportunities within the community.[40] It is widely known that parental education is strongly associated with child health and development outcomes,[41-43] including positive school outcomes for children.[44] Higher parent education is often linked with better childhood outcomes.[45-47] How community factors influence ECD may be through providing opportunities and resources to help support parents and carers raising their children.

Highest level of schooling *captures the highest level of education (Year 12 or equivalent) achieved by residents living in the suburb.*

This could be due to a number of reasons. Beyond creating an important economic resource, human capital such as higher education is important for neighbourhoods because adult residents who are educated and/or employed can shape the type of role models children are exposed to outside the home;[45, 46, 48] they can encourage behaviours and attitudes that help foster success in school.[48] As such, children are more likely to value education, adhere to school norms, and work hard because of the adult role models provided in the neighbourhood.[49]

Neighbourhoods can also influence educational outcomes through its scope and quality of social capital (social networks and social support); children's exposure to social networks that offer positive resources, helpful information, and educationally beneficial opportunities may be linked with better outcomes for children.[49, 50] Institutional resources allocated to schools, libraries, education facilities, as well as community-based initiatives to support educational programs, can influence indicators of school achievement such as reduced attrition rates and high school graduation and attainment.[44] Access to and use of these resources can depend on parental education.[43]

3.2.2 What KiCS found

In KiCS, local communities where children were doing better than expected had a higher proportion of residents who achieved Year 12 education than the local communities where children were not doing well, despite being socio-economically similar.

Highest level of schooling provides an objective indication of the education levels of residents within the suburb. While it was measured both qualitatively and quantitatively in KiCS, level of education only appeared to be a differentiating FCF when measured quantitatively. The quantitative measure could potentially be an indicator.

HIGHEST LEVEL OF SCHOOLING	What are we measuring?	How we measured it
Quantitative	Highest level of schooling – Year 12 or equivalent	Australian Bureau of Statistics data

Quantitatively, the ABS Census data has reliable information on Highest Year of Schooling - completion of Year 12 or equivalent. While university, postgraduate and extra qualifications may be obtained, this indicator measures universal or standard education requirements available to the Australian population. It is an important indicator of highest level of education attainment, and signals educational need and disadvantage, and is used for planning and profiling at the small area level.[51]

3.2.3 Limitations with measurement and use

Highest level of schooling is a measure of education levels and may not necessarily be linked with income as occupations in various industries and markets differ considerably. Highest level of schooling is linked to other FCFs in the list (e.g. see **Gentrification section 3.3**).

Neighbourhood-level differences might account for 5-10% of the variability in school achievement.(5, 44) At the community level, public services and facilities available to residents may affect the personal development and opportunities of residents.(52) Highest level of schooling can be used at a local level to perhaps plan for programs and initiatives that may help support members of the community receive qualifications of Year 12 equivalent. In turn, more aspirational communities may advocate for better living conditions and outcomes for children.

3.3 Gentrification

3.3.1 Relationship with ECD

Gentrification *is a process of neighbourhood change that implies a changing residential class-income profile.[1] It captures the influx of relatively higher income neighbours.*

Gentrification is a process of socio-economically selective migration that sees higher income and higher consuming households move into lesser valued, 'more affordable' urban areas where their investment sees more "bang for the buck".[53] As such, gentrification is not just a characteristic of the urban growth, it includes changes to the community's SES profile.

Gentrification changes the neighbourhood and family contexts in which children live – for better or worse – depending on whether you stay or leave the gentrified neighbourhood,[54] and can be perceived positively and negatively.[1] Political debates surrounding gentrification and mixed-income development are examples of social hierarchies created at a level upstream of the neighbourhood, which may consequently have a role at the neighbourhood level in promoting or mitigating developmental inequalities.[40]

Gentrifying areas tend to experience noticeable upward movements in social status, as revealed in increases in households characterised by high incomes, university-level qualifications, and employment in professional positions. In gentrifying areas, demand for housing increases and property values rise; poorer residents may be displaced as wealthier people move in. This may also result in new shops appearing, and the public image of the neighbourhood changing. Along with services and amenities, neighbourhood safety may improve with gentrification.[55]

Gentrification also implies the displacement (outflow) of lower-income residents. Levels of displacement has been a contentious issue because it may further concentrate poverty cycle for the more disadvantaged. However, a study found that poorer residents were less likely to move out of gentrifying neighbourhoods than non-gentrifying neighbourhoods.[56] Nevertheless, while the end-state of gentrification may be desirable (e.g. more income-diverse suburbs, more resources in disinvested communities),[54] it may result in problems for very low income families who disproportionately receive fewer benefits that gentrification has for others.

3.3.2 What KiCS found

In KiCS, disadvantaged local communities where children were doing better than expected were gentrifying more than the disadvantaged local community where children were not doing well. Gentrification emerged as a qualitative theme in interviews and focus groups, which focuses on the influx of affluent neighbours, and the outflow of lower-income residents.

GENTRIFICATION	What are we measuring?	How we measured it
Qualitative	Influx of relatively higher income (affluent) neighbours, and outflow of lower-income residents	Interviews and focus groups

In KiCS, participants mentioned relatively higher income families (although potentially still disadvantaged) moving into the disadvantaged area resulting in the displacement of more disadvantaged groups. 'Wealthier' relatively less disadvantaged (higher SES) families are reportedly moving into the disadvantaged local community doing well on ECD, primarily due to more affordable housing stock. They were more likely to be new families with very young children, thus potentially having reduced income due to the primary care giver not being in paid employment. Public renters are usually not included in gentrification measures because of the security of tenure provided in public housing (once in the public housing system). The KiCS findings show that the *most* disadvantaged in *already disadvantaged* areas may receive fewer benefits of gentrification compared with others in the community.

The KiCS' findings show that the most disadvantaged in already disadvantaged areas may receive fewer benefits of gentrification compared with others in the community

Some KiCS local communities mentioned potential positive benefits of gentrification for children from more disadvantaged families. For children from very low income families, perceived generational poverty can be a barrier to their aspirations if they lack positive role models among their family and neighbourhood environment. However, seeing how 'different' people live and being exposed to more 'affluent' neighbours may make a difference to children aspiring to something better for themselves:

> ***"[Referring to disadvantaged community doing poorly on ECD] It's also how your neighbours live. They expect the handouts. Whereas in [disadvantaged community doing well on ECD] it's not so condensed and confined. Your neighbours drive a nice car, if they work. They can see how the other half live and they want to aspire to that'*** (FG45, service provider)

3.3.3 Limitations with measurement and use

Gentrification is often a process *uncontrolled* by governments but driven by housing markets, for example. Thus it is closely linked to another FCF in the list, **Housing affordability (section 3.4)**, and to others such as stigma and income. Gentrification may be a potential positive change for disadvantaged children who remain in the neighbourhood because it can impact the socio-economic profile of the community as a whole, and potentially attract more resources into the community. However, there are potential unintended negative consequences, and these could be areas of action to consider at the community-level:

- The influx of higher income families may drive the outflow of lower-income families.
- Disruption to community harmony and social processes may occur because of high levels of residential mobility. [4]
- Gentrification could signal housing (un)affordability issues in the area; programs may be considered to ensure lower-income families have more secure housing tenure or have a sense of being 'included' (i.e. foster positive social processes) within the community.

Measurement:

- Our measure of gentrification is qualitative, but quantitative gentrification measures have been used in previous studies. For example, gentrification can be explored through individual indicators of neighbourhood composition e.g. tenure change or a composite measure of multiple measures (e.g. tenure change, occupation status and income).[1] Repeated cross-sectional measures of neighbourhood composition can track trends of communities over time; these can be used as quantitative indicators of gentrification activity alongside our recommended qualitative measure of gentrification for further context.
- Separating gentrification and displacement from wider processes of social change, voluntary movement, and welfare and labour market changes add complexity to measurement.[1] Households may be displaced as rental rates increase through lack of available housing supply. Measuring the likely out-movers from gentrification is recommended, while considering that processes of voluntary movement continue to impact many households' decisions. The most effective measures of gentrification and displacement processes require data that track people moving in and out of the local area so that any confusion arising from changes in personal or household status is accounted for. Detailed data of this sort were not collected in KiCS.

3.4 Housing affordability

3.4.1 Relationship with ECD

Housing affordability *refers to the relationships between household income and spending on housing.*

Housing affordability and rising house prices may negatively influence ECD. It is closely linked to the adverse effects of income and poverty.[57] Housing affordability has been postulated to affect ECD in two ways:

1. Rising housing costs may place *material hardship* on families. Income enables families to purchase material goods and services that benefit children's development and wellbeing.[58] Yet if housing costs represent a significant proportion of a family's income, the amount of disposable income left to spend on other living costs such as clothing, school activities, food, and health care is limited. [59] Housing affordability issues may influence families having to re-locate neighbourhoods which can also reduce access to health, education and social services.[60] Housing affordability stress is usually considered when housing cost burden is near or above 30% of income – the long-standing rule-of-thumb definition of affordable housing.[58] This means indirect effects on children's ECD; families with high housing costs might be forced into lower quality housing or reduce consumption of basic necessities such as food and health care, which may lead to poorer outcomes for children.[57] Others have found that unaffordable housing affects children *most during early childhood* via its adverse impact on the family's ability to access basic necessities.[61]

2. Rising housing costs may induce family stress caused by *financial hardship*. Unaffordable housing indirectly affects children's outcomes by inducing parental stress and anxiety or depression due to financial hardship, which in turn can lead to an increased likelihood of inconsistent and punitive parenting behaviours towards children.[62] Studies have shown that stressful life events in the family such as economic hardship and employment difficulties can be problematic for children's development and mental health.[57]

3.4.2 What KiCS found

In KiCS, housing affordability was less of an issue in local communities where children were doing better than expected compared with local communities where children were not doing well, despite being socio-economically similar. Lack of affordable housing (ownership and rental) was also consistently identified as an important factor across KiCS local communities.

HOUSING AFFORDABILITY	What are we measuring?	How we measured it
Qualitative	Housing affordability being an issue for disadvantaged groups	Interviews and focus groups

Housing affordability was measured qualitatively and quantitatively in KiCS. The quantitative measure explored if the proportion of households in the bottom 40% of income distribution paid more than 30% of their household income on housing costs.[63] While the '30/40' rule is a commonly used quantitative measure, it did not appear to predict why children were doing better or worse in the KiCS' communities at the suburb-level.

Housing affordability was a qualitative factor consistently associated with disadvantaged local communities doing well on ECD, compared with disadvantaged local communities doing poorly. While housing affordability could be seen as potentially *modifiable* at the policy level through state and federal governments, KiCS found that this FCF focuses on housing affordability being an issue for disadvantaged groups which can be an unintended flow-on effect of the gentrification process (see **Gentrification section 3.3**). There are relatively 'wealthier' (less disadvantaged) families moving into more disadvantaged areas due to perceived housing

affordability (relative to other suburbs), which can have a negative influence on the community (e.g. high residential mobility). An example from KiCS data is that more disadvantaged groups suffering even more disadvantage as private rentals become more expensive due to changing SES profile (gentrification) and inner-city urban development becoming more unaffordable.

3.4.3 Limitations with measurement and use

Housing is one of the most basic needs for families, yet rising housing costs are increasingly placing pressure on many Australians.(63) The impact of higher housing costs is most strongly felt by lower-income groups, particularly low-income renters for whom home ownership is increasingly out of reach.(60) Monthly rental repayments may also increase in gentrifying areas, which means that lower-income renters may need to search for more affordable housing in other suburbs.

A substantial part of responses to housing affordability issues for families' needs to occur at a policy-level. Locally, better monitoring of housing affordability may signal ways in which communities can help at the community level. For example, with housing stress posited as a material and financial hardship for families,(57) family problems are often located in external events, such as poor housing or unemployment. Yet these stressful conditions are insufficiently explored despite its potential impact on the quality of interpersonal relationships and parenting style.(64) A report by the Australian Institute of Family Studies (AIFS)(63) highlights that local practitioners should include housing tenure and quality in routine assessments of their clients, and help clients explore ways to improve their living conditions (e.g. referrals to appropriate services, advocate to real estate agents for repairs to property). It seems important that service providers recognise housing issues as being possible underlying problems that place considerable stress on families, including their health and wellbeing. General themes regarding clients' struggles with housing may also be useful to consider and promote in ways that may impact on the macro-level drivers of housing stress.

Some caveats associated with housing affordability:

- Housing affordability may be linked with other FCFs mentioned here e.g. income, stigma, gentrification, and housing tenure (stability). In gentrifying areas, insecure tenure in private rental accommodation may occur as rental prices increase; insecure renters face eviction or market dislocation.(53) Such dislocation means lower-income families may need to move neighbourhoods; insecure tenure, high residential mobility, and less neighbourhood stability are stressful situations for parents with young children (see **Housing tenure section 3.5**). Being 'priced-out' of locations may have flow-on side effects such as increased commuting times to work, increased barriers to accessing essential services (e.g. schools) and changes in the quality of the neighbourhood they move to.(54)
- Housing affordability was measured qualitatively, but there are ways to measure this quantitatively. The most common way to measure housing affordability is the '30/40' rule. That is, households in the bottom 40% of income distribution are paying more than 30% of their household income on housing costs.(63) While this is a commonly used measure, it did not appear to predict why children were doing better or worse in the KiCS' communities at the suburb-level. Communities may want to collect this information from the ABS Census data for context alongside people's perceptions of housing affordability in the area.

3.5 Housing tenure (stability)

3.5.1 Relationship with ECD

Housing tenure relates to renting and private home ownership within the suburb. Home ownership has been associated with less behavioural problems,[65, 66] and better educational outcomes in children.[65] Previous studies suggest that it is not home ownership *per se* that produces benefits for children, but home ownership indirectly affects child development through other mechanisms.[57] For example:

__Housing tenure__ relates to renting and private home ownership within the suburb and reflects housing stability or security.

- Home ownership provides people a sense of independence, certainty, and control that may lead to lower stress levels for families; effects on parent mental health is associated with parenting styles and family stability, which in turn can influence children's outcomes.[67]
- Home ownership may be beneficial for child development because it provides families with greater opportunity to maintain more consistency in daily routines, social interactions, and life experiences.[65] For children, it may mean that children have greater consistency and stability in their lives, fewer school transitions, and more stable environments.[57, 65, 67]

Thus, home ownership is more likely to reflect *secure housing*, and longer tenures; families who own homes may be less likely to move compared with families who rent. This increases residential *stability* (and decreases mobility or transience), and generally minimises the number of school transitions for children.[57] At the community-level, less residential mobility or transience in the neighbourhood may influence stronger and more stable connections between families and other community members, and facilitate feelings of neighbourhood attachment.

3.5.2 What KiCS found

In KiCS, there were fewer renters (and more home ownership) in local communities where children were doing better than expected compared with local communities where children were not doing well, despite being socio-economically similar. Housing tenure (stability) could potentially be an indicator.

HOUSING TENURE	What are we measuring?	How we measured it
Quantitative	Proportion of privately owned dwellings vs. renters in the suburb	Australian Bureau of Statistics data

Housing tenure relates to the proportion of people renting compared with private home ownership within the suburb. In particular, private renters may be a proxy for the degree of transience or residential mobility within a community. It can signal insecure renting tenures, which may have flow on effects for frequent residential moves; this has been shown to have a negative impact on educational outcomes for children.[68] It excludes public renters because although they have less control of the housing they receive, they may have more secure tenure than private renters (once processed in the system).

3.5.3 Limitations with measurement and use

Housing tenure may not necessarily reflect home ownership *per se*, rather it may signal *secure* housing tenure. Housing policies may be able to respond to this through extending leases for private rental accommodation (in Australia, a one-year lease is common) so that longer-term opportunities and inclusion for those who cannot afford a home can be fostered. Moreover, housing policies could limit rental price increases for the most disadvantaged groups. In this way, a broader range of income groups can be accommodated, and in doing so, may also impact on the levels of residential stability in the community.

While living in a suburb with fewer renters is good for child development in and of itself, home ownership likely reflects wealth and higher incomes e.g. compared with suburbs with lower rates of home ownership, suburbs with higher home ownership rates are richer, which may drive differences between why some communities are doing better than others in terms of ECD (see **Income section 3.1**).

While it is difficult to use housing tenure data to inform ECD policy, it may be collecting information at the local level may allow communities to respond to providing support for families in unstable accommodation to lobby for longer leases, find new homes within the same or surrounding areas, and assist with moving.

3.6 Public housing

3.6.1 Relationship with ECD

***Public housing** relates to subsidised long-term, low-cost housing options for people on low incomes.*

This FCF does not focus on private housing. Public housing refers to social housing and community housing. Public housing is subsidised by the government, while community housing is managed by non-for-profit agencies but are regulated by state governments. For low-income earners, public housing represents a low-cost housing option that may promote housing affordability through rental subsidies and greater security of tenure (than private renters).[60, 69] People who are eligible for public housing include people on low incomes who are unable to rent privately and people that are most in need (such as those who have recently experienced homelessness, family violence, or have other special needs). Public housing makes housing more affordable by providing subsidised rentals and greater security of tenure (than private rentals).

Those living in public housing developments are more likely to be exposed to more crime, high unemployment, or other social issues within a concentrated area.[70] Aside from social issues that may occur in public housing areas, there are other reasons why public housing may influence ECD. For example, although not always the case,[71] substandard housing may be more likely for those living in public housing developments. Both structural quality and maintenance of the home affects neighbours' perceived quality of their environment and often influences how others view residents living there.[72] Residents may feel stigmatised by the larger community and may internalise other's negative perceptions of them.[73] The stigma attached to public housing and 'bad' neighbourhoods can influence self-esteem. The house is a symbol of self, reflecting who we are, what we have accomplished and what we stand for.[73] Moreover, without provision of semi-public space and facilities around public housing, families may be more likely to stay indoors and do not have opportunities for facilitating informal social networks, the social support, protection and informal social control found in other disadvantaged neighbourhoods.[74]

3.6.2 What KiCS found

In KiCS, there was less public housing in local communities where children were doing better than expected compared with local communities where children were not doing well, despite being socio-economically similar. If public housing was present, the type and location of public housing seemed to make a difference. For example, disadvantaged local communities that had better ECD scores than other disadvantaged local communities had public housing that was 'scattered' across the local community, and 'lower-density' housing types (e.g. detached single housing rather than higher-rise townhouses and units). In contrast, disadvantaged communities doing poorly on ECD scores were often characterised by the notable presence of public housing or distinct areas of the suburb of public housing estates.

The KiCS findings show that it is not the mere presence (or absence) of public housing that might differentiate communities… it refers to how public housing is distributed across the community and the housing type that may help ameliorate stigma associated with living in public housing.

The presence (and amount) of public housing was measured both qualitatively and quantitatively, and both seemed to be consistently associated with disadvantaged local communities doing well on ECD compared with other disadvantaged local communities. It provides a snapshot of the social housing tenure within the suburb. In particular, social housing (public housing or community housing) represents an indication of lower income

people within the suburb that need housing assistance. Public housing location and type were measured qualitatively only. The quantitative measure could potentially be an indicator.

PUBLIC HOUSING	What are we measuring?	How we measured it
Qualitative	Perceived amount of public housing within the suburb	Interviews and focus groups
Qualitative	Perceived public housing location is scattered (rather than concentrated) across the suburb	Interviews and focus groups
Qualitative	Perceived public housing type (e.g. townhouses, units vs. detached single homes)	Interviews and focus groups
Quantitative	Proportion of residents who are public renters within the suburb	Australian Bureau of Statistics data

The KiCS findings show that it is not the mere presence (or absence) of public housing that might differentiate why some disadvantaged local communities are doing better than others on ECD. Rather, it refers to how public housing is *distributed* across the community (e.g. located in concentrated pockets or otherwise 'scattered'), and the housing type that may help ameliorate stigma associated with living in public housing. For example, participants referred to public housing as 'not being so obvious' or it 'looked like any other house' if it wasn't higher-rise density housing types located together in the same area. Indeed, policy responses have included deconcentrating poverty and integrating residents into communities in which their residents are not different from their neighbours. Such efforts might reduce the stigma associated with residency in traditional public housing.

> ***"One of the issues with public housing is, that it can be grouped together in say, small sizes in terms of inside their home and then only in the one spot. It's a bit different if you're in a house, with just one neighbour there, whereas [if] you're in a group of a dozen or something and then a couple of people are a bit volatile or whatever. That affects everybody in a close area"*** (INT070)

3.6.3 Limitations for measurement and use

Many disadvantaged suburbs with higher than average numbers of public housing may not only experience material hardship, but also suffer from poor community reputations reinforced through stigmatising stereotypes associated with public housing residents. Some caveats with the public housing FCF for consideration include:

- Need to understand the context of public housing in the community
- Public housing is linked to other FCFs in this report, such as stigma, 'higher-rise' density living and income, and possibility historical events (e.g. if the community was traditionally a housing commission suburb)
- Communities may want to consider holding focus groups with public housing residents to better understand their concerns about the community (e.g. stigma) and what communities can do to respond. A quote from one of the KiCS communities shows that facilitating a sense of community may help:

> ***"There's a ready supply of public housing for people in significant need so that's a good thing There's still a sense of community within that area and certainly something that the schools and pre-schools reinforce either in their space or geographically nearby. I think that those within that space view it in a very positive way"*** (INT082)

3.7 Housing density

3.7.1 Relationship with ECD

Higher density living may negatively affect children's development outcomes,[75] but the findings are indicative rather than conclusive.[76, 77] The potential mechanisms between density and ECD are complex, and likely to be affected by SES, and mediated by parent mental health, neighbourhood satisfaction and perceptions of the environment.[78]

__Housing density__ relates to higher-rise living (townhouses, units rather than single detached dwellings). It does not necessarily reflect very high-rise living such as apartments. This FCF must be read with the __Public housing FCF__.

Neighbourhood satisfaction is often linked with perceptions of the neighbourhood,[79] including safety.[80] Neighbourhood safety concerns may influence family practices and parental restrictions thereby impacting children's opportunities to play outside,[81] and interact with others locally.[82] For example, Evans[83] identified mothers of young children living in high-rise developments expressed difficulties in monitoring children's outdoor play because of crime and safety concerns.[83] Similarly, Whitzman and Mizrachi (2012) found children living in high-rise housing were similarly concerned about traffic volumes and lack of safe crossing points in their neighbourhood.[84] This is unsurprising given higher density housing is frequently located along major arterial roads, which often attract more vehicular traffic and strangers into the local area [85]. Indeed, parent concerns about traffic and strangers are among the most highly cited barriers to children's engagement with the outdoors.[82, 86]

3.7.2 What KiCS found

In KiCS, there was less high(er) rise density housing in local communities where children were doing better than expected compared with local communities where children were not doing well, despite being socio-economically similar.

HOUSING DENSITY	What are we measuring?	How we measured it
Qualitative	Perceived higher-density residential living in the suburb (*but associated with public housing*)	Interviews and focus groups
Quantitative	Proportion of dwellings three or more storeys in the suburb	Australian Bureau of Statistics data

Housing density refers to both perceived higher density residential living (e.g. higher-rise density accommodation such as apartments, townhouses, units) and actual higher density residential living as measured by ABS Census data (three or more storeys). However, it appears to be closely linked to another housing FCF, '**Public Housing**'. Thus, it should be read as a complementary FCF to Public Housing.

Qualitative findings from KiCS indicated that higher-rise density living was linked to public housing.

3.7.3 Limitations with measurement and use

High-rise density living (regardless of SES), has been linked to neighbourhood dissatisfaction, which is often linked to perceptions of neighbourhood safety concerns.(80) Families with young children often prefer to live in detached houses because of the provision of more 'space' and back/front yards.(80, 87) However, if designed properly, high-density housing may be a favourable housing option for families. (78)

Qualitative findings from KiCS indicated that *higher*-rise density living was linked to public housing. As such, the qualitative measure is not distinct to high-rise density living *per se* unless it is specifically raised as an issue. Nevertheless, high-rise density in outer suburban areas and larger regional towns is more likely to be public housing, and communities themselves would know whether high-rise density housing is designated public housing and may want to take this into account. Some other caveats to consider:

- The quantitative measure of public housing from the ABS is distinct to high-rise density living for private dwellings. It is not linked public housing.
- In KiCS, a *mix* of public housing types (e.g. higher vs. lower density) seemed to make a difference, and the location of public housing (concentrated vs. scattered) within the suburb are possible ways governments may respond (see **Public Housing section 3.6**). Qualitative findings around these suggestions were mostly around the subtle (rather than 'obvious') distribution of public housing across the community, and this is perhaps related to improving feelings of stigma attached to these areas.
- Accommodation considered below three storeys is considered 'low-rise' in KiCS' findings even though previous studies have considered greater than four storeys as high-rise.(80) This is because many of our KiCS' suburbs were located in outer suburban or large regional areas, which typically precludes very high density living.

If high-rise living plays a direct or indirect role on ECD outcomes, then how should we design high-rise density public housing and its surrounds to increase its acceptability to families and the community? From a built environment perspective, this places increasing pressure on considering interior and exterior building design (e.g., natural surveillance opportunities such as windows facing the street), identifying optimal housing density levels, identifying elements that encourage neighbourhood satisfaction (and perceptions of safety), recognising neighbourhood features that promote and hinder children's healthy development and wellbeing, and thus co-locating high-rise density housing in neighbourhoods with these features.(34) For example, Coley et al. (1997) found that high-rise public housing with spaces with trees attracted larger groups of people, and a mix of youth and adults compared with those spaces devoid of nature. Such spaces increase opportunities for social interaction and passive supervision of children in lower-income neighbourhoods.(88) Communities may want to discuss how best to design spaces around high-density environments that may help facilitate social interaction and feelings of neighbourhood safety.

3.8 Stigma

3.8.1 Relationship with ECD

***Stigma** refers to the negative reputation of a community.*

Communities with more stigma were consistently characterised by high crime and/or drug use, high levels of public housing and high levels of unemployment.

Stigma may reduce the opportunities and affect the perceptions of those living in stigmatised areas in a variety of ways, such as job opportunities and self-esteem.(52) Such negative associations of living in neighbourhoods with stigma can have a negative impact on children.(73) Community stigma can be perpetuated through media and through children's indirect exposure to negative judgements of parents and other family members. It results in the risk of being judged, stereotyped, and consequently, children may experience bullying. Such negative assumptions seek to devalue or discredit these marginal people from full social acceptance.(89, 90) Growing up in areas with a negative reputation may likely affect children's self-esteem and aspirations for the future.

3.8.2 What KiCS found

Community reputation emerged as the most consistent (strongest) factor for differentiating all matched-disadvantaged pairs in KiCS; the local communities where children were doing better than expected had a better reputation (less stigma) than local communities where children were not doing well, despite being socio-economically similar.

STIGMA	What are we measuring?	How we measured it
Qualitative	Perceived negative reputation	Interviews and focus groups

Stigma emerged as a qualitative factor that helped signal a difference between disadvantaged local communities doing better than other disadvantaged local communities. This factor focuses on perceived negative reputation, and how people perceive others to 'judge' or 'stereotype' them from other members of society, based on where they live. Stigma can be attached to particular pockets or areas within the community e.g. schools, housing estates, or the negative reputation can be attached to the whole community. Both these aspects are encompassed in this factor. This factor was not measured quantitatively in KiCS.

> ***"Generally, I believe we're just like a village. People have such a … unfortunately, it's a stigma that goes with our area, from a history perspective. "There's always trouble in [community]," or, "You don't go over the bridge because of the people from [community]," but if they only lived here, they'd understand, just open the door a little bit to who we are as a community. Extremely supportive, very supportive, and very friendly and welcoming community. Every community's got its troubles, but it's a very friendly township, really, of people"*** (INT027)

3.8.3 Limitations with measurement and use

Negative reputations can manifest from particular reasons, which can be determined through qualitative measures. Having a clear understanding of the reasons stigma has come about can assist in the development of interventions to address stigma. In KiCS, community stigma appears to be closely linked with the concentration of public housing within a community, (i.e. the community is characterised by its public housing areas) as well as other KiCS FCFs such as income (i.e. economic diversity) and gentrification. As these FCFs appear to be related, their influence in the community context must be further considered. It may be that high-rise density developments in these areas are mostly designated public housing, or that there are distinct pockets with high concentration of public housing which have stigma attached to it.

Community stigma can be longstanding and entrenched in the characterisation of the community, thus it may be difficult to modify in the short term. However, initiatives that address stigma (e.g. neighbourhood renewal projects) can be a starting point for building community skills, voice, and connection, which subsequently can work towards addressing stigma. Some studies suggest that there are ways in which residents living in stigmatised areas and housing actively resist and challenge negative reputations ascribed to them, which has implications for policy.[89] For example, those who are actively involved and committed to their community, have a sense of pride in their suburb and have coping strategies to resist popular portrayals that continue to stigmatise them.[89] This also happened in one of the KiCS local communities; a long-standing sense of pride may be protective against stigma. It may be that residents' perceptions of their housing and location are different to those living outside of their neighbourhood e.g. 'externalised' stigma through persistent negative reputations.

3.9 Service reputation (primary schools)

3.9.1 Relationship with ECD

Service reputation of primary school *refers to whether the primary school is considered 'good' or not. In other words, it refers to the positive or negative perception of the school.*

Service reputation is the positive or negative perceptions of services. For primary schools, service reputation appears to strongly influence its use, creating high demand of well-reputed schools and conversely low demand for poorly-reputed schools. Indeed, a main factor for parents' choice in where to live is school reputation; parents (who have the resources to move) will consider moving to suburbs that have 'good' schools.

There is increasing recognition of the importance of school reputation within the academic and education sectors.(91, 92) International studies highlight the difficulty, for parents as well as school management, to empirically evaluate a school's reputation due to the intangibility of the service. International studies suggest that an antecedent of school reputation is parents' satisfaction with a school, which consequently builds their loyalty and commitment to a school, thus further reinforce a schools' reputation.(92) School reputation may be a signal for perception of school quality; previous studies have explored measures of school and preschool 'quality' such as principals' and teachers' years of experience and development, collaborating with other schools in a 'school family,' quality of teacher-child relationships, school policies, and meetings between parents and teachers. (93) Such factors may have positive impacts on school readiness, and students' test scores.(94, 95)

3.9.2 What KiCS found

In KiCS, the reputation of primary schools, in particular, was found to differentiate ECD outcomes in disadvantaged local communities, but also considered an important factor for ECD for the majority of local communities. The reputation of schools was rarely characterised by its literacy or numeracy results. Schools that were perceived as 'good' or well-reputed were often characterised by the workforce within the school, such as the principal, teachers, and staff having an acute awareness of, and interest in the needs of the children that attend the school.

> ***"We've had a change in the last couple of years in headmaster, well the principal, and he's a lot more community-focused, so we're getting a lot of people who are out of region coming, traveling, choosing to come. I think it's also retaining numbers also."*** (FG034)

Conversely, parents described poorly-reputed schools with principals and staff that did not listen, nor try to assist their child when help was needed. Negative school reputations were also reported for schools that were stigmatised based on the negative perception of the community or its proximity to public housing. Studies have highlighted that without any physical goods to evaluate a school's service, there is greater reliance on anecdotal evidence making a school's reputation important.(92)

> ***"I'm very impressed actually. I had one of your teachers actually come in and go above and beyond one of our children coming in here, which I've not seen in the past. We have lots of families in early childhood who come to us and ask us, "What do you think of this school? What do you think of that school?" That's definitely made an impression on me."*** (FG050)

For KiCS local communities, there were instances where poorly reputed schools resulted in families selecting alternative schools for their children to attend where possible. Remaining at the school were families who did not have the resources or ability to choose (e.g. due to barriers such as transport or cost), which may have further perpetuated the school's negative reputation.

> ***"There's still a stigma attached even though there's some fantastic teachers in there."*** (INT028)

The negative reputation of a school can influence families' choice to enrol their children in the school, feelings of loyalty and ongoing involvement with school activities, as well affect children who attend the school. Parents may select schools with a positive reputation, however, communities that rely on school zoning can lead to a reduced choice of schools for families and result in attending a school that is ill-suited to their child's needs, thus causing a negative experience and poor perception of its quality. Families that have the resources to move to more well-reputed schools can perpetuate negative perceived quality as the school has students who do not have the choice but to stay enrolled. Addressing the potential negative implications of school zoning (e.g. reduced choice for families) can be considered through local government and state ECD policy.

Perceived service quality	What are we measuring?	How we measured it
Qualitative	Positive or negative reputation of schools, whether the school is considered 'good'	Interviews and focus groups

Service reputation (primary schools) is an FCF that is measured qualitatively, through the perceptions of service quality. While KiCS attempted to quantitatively measure service quality (for which a global measure is lacking), quantitative measures used neither differentiated diagonality, nor was directly linked with perceived service quality of primary schools.

3.9.3 Limitations with measurement and use

Primary school reputation is linked closely with stigma, either attached to the community or the localised place (e.g. the primary school itself). While stigma attached to a community and school can be entrenched, the reputation of primary schools perhaps has more potential to be modified at the local level, through better engagement and stronger ties with its school members and wider community:

- Ensuring the school's workforce has the capacity (e.g. through programs or professional development activities) to support children and their families' needs, principals and leaders can provide support and actively ensure their awareness of the needs of the children. For example, understanding and appropriately responding to home situations that can be a barrier to school attendance.
- Addressing the potential negative implications of school zoning (e.g. reduced choice for families) can be considered through local government and state ECD policy.
- Engagement through events with local parents and the wider community – school bonding has been associated with healthy development.[96] This can assist in building school loyalty.
- Actions to respond may vary between communities depending on how the perceived reputation of the school came about.
- The reputation of other education settings, i.e. ECEC services, was also found to be an important factor in communities, however, did not emerge as a factor that differentiated ECD outcomes.

3.10 Perceived ECEC availability

3.10.1 Relationship with ECD

***ECEC availability** refers to the perceived number of local childcare centres within the community.*

Availability of Early Childhood and Education Care (ECEC) refers to the reported quantity of childcare centres within the community. Attending childcare, preschool, and kindergarten programs have been associated with children's learning, socio-emotional development, and other ECD outcomes.[97] Successful developmental outcomes, however, depend on availability and quality of early childhood programs.[98] International evidence indicates quality early childhood programs that impact positively on children's social and cognitive outcomes are cost-effective, and yield improved educational performance for all children, especially for those from disadvantaged backgrounds (Lynch, 2005 in[98]).

For the most disadvantaged groups, more months spent in child care centres has been associated with better maths scores among children with less educated mothers and a poor literacy environment at home.[99] Moreover, in areas where child care supply is limited, children's language scores and mothers' employment improve with every percentage increase of ECEC coverage.[100] Implications include the need for strong parental involvement in children's development and subsidised child care for children in need.[99]

3.10.2 What KiCS found

A recent Australian study showed that there were fewer ECEC in disadvantaged areas, and these programs provided a lower average quality of care compared with more advantaged areas.[101] Further studies highlight participation in ECEC is greater in more advantaged areas.[102] In KiCS, we found perceived availability of local ECEC was one factor that consistently pointed to why local communities had better ECD outcomes than local communities with poorer ECD outcomes. ECEC availability, however was not considered important or a concern in the majority of local communities.

KiCS local communities discussed the availability of local kindergarten/preschool sessions differently to long day care centres, often in relation to parental work hours and employment situation. Disadvantaged local communities doing poorly in ECD outcomes were not as frequently discussing ECEC availability nor location, costs or transport as barriers to attendance. It may be that in these communities there was less discussion of parental employment which is a related factor of perceived ECEC availability (see **Limitations section 5.2**).

> ***"They [ECEC centres] are only in [community] that I can walk… Otherwise, I have to take the car. People that don't feel it['s] annoying because they grow up with cars…. For me it's very annoying. You have to take the car for almost everything you want."*** (FG047)

ECEC availability	What are we measuring?	How we measured it
Qualitative	Reported/perceived number of childcare centres within local area	Interviews and focus groups

This is a qualitative measure that does not necessarily relate to the absolute number of ECEC centres in a community, but rather the perceived availability of ECEC services. This has implications on ECD policy in the planning and establishment of ECEC services within a community as well as addressing financial and physical accessibility to ECEC services for families. Lack of local ECEC service provision may have inevitable negative consequences for children, especially for the most vulnerable and disadvantaged communities.

3.10.3 Limitations with measurement and use

- Quantitative measures (through GIS) for ECEC availability did not show significant differences between communities; Quantitative measures (community survey) found a difference in the perceived proportion of childcare/occasional care services. However, the community survey had significant response bias, in terms of representative sampling (the majority of survey responders were community members who were older adults, and may not use ECEC services).
- Cost of ECEC was found to be important and may play a role in this FCF – KiCS local communities often highlighted the tension of balancing government subsidies for ECEC places and the requirements for staff to child ratios, whilst ensuring it is a financially viable operation. For parents, ECEC costs were not affordable even with government subsidies especially for disadvantaged communities and families that were not working, or had limited hours of work. A study suggests that when parents are asked to respond to questions surrounding the overall difficulties with obtaining childcare, they do not separate out the quality from the cost, from the availability; raising the concern that cost is always influencing their response regarding availability rather than taking into account the location.[103] This could suggest that this factor is linked to other FCFs e.g. Income.
- Closely related to ECEC availability is labour and workforce conditions of parents. It could be that families that are seeking childcare as a means to return to work, whilst very disadvantaged local communities who are not seeking or unable to attain employment are not seeking childcare options.

3.11 Perceived crime

3.11.1 Relationship with ECD

***Perceived crime** refers to the negative attributes of safety concerns, vandalism and antisocial behaviour within a neighbourhood.*

Neighbourhoods characterised by safety concerns, garbage/litter in the streets, and vandalism have been associated with a number of health behaviours and outcomes, including children's overweight and obesity,[104] behavioural problems,[105] and other child development outcomes.[106, 107] Communities characterised by high rates of crime and delinquency tend to have high rates of infant mortality, low birthweight, tuberculosis, child abuse, and other detrimental aspects of child development.[108] Perceived crime in the neighbourhood may contribute to parental perceptions of their surroundings, and in turn limit interaction and use of public space.[83] Neighbourhood safety concerns such as 'stranger danger' are among the highest cited barriers to children's access to the outdoor environment.[82, 109]

3.11.2 What KiCS found

In KiCS, perceptions of crime were found to differentiate ECD outcomes in disadvantaged local communities, but also considered an important factor for the majority of communities. Perceived crime and fear of crime does not necessarily relate to actual crime rates, and there are many studies that have found that fear of crime does not necessarily reflect the actual crime.[110, 111] Often negative attributes of crime within a local community were reported by people who did not live in the area, which may indicate this FCF is also linked with external stigma (see **Stigma section 3.8**).

Perceived Crime	What are we measuring?	How we measured it
Qualitative	Perceived crime safety in the local community	Interviews and focus groups

Some examples of the effects of crime and safety concerns from communities include:

- Parents limiting play opportunities because they perceived it was 'unsafe' to play
- Pockets of areas associated with high rates of crime, drugs and social disorder issues
- Perceived crime limits access to the neighbourhood, and affects trust in neighbours

3.11.3 Limitations with measurement and use

Perceptions of crime are often not correlated with actual crime. Nevertheless, perceived crime can have a considerable influence on feelings of safety, neighbourhood attachment, and a sense of belonging, which can subsequently affect parent and child behaviours.

There may be ways in which communities can respond. High levels of neighborhood social capital and collective efficacy (i.e., shared expectations, residential 'monitoring' of children's behaviour and intervention if necessary) [18] have been linked to healthy child development and behavioural outcomes, even in disadvantaged communities with fewer financial and educational resources available.[112, 113] Developing high levels of social capital and collective efficacy is complex, however, helping to facilitate these social processes could be encouraged through better 'Eyes on the street' (passive or natural surveillance), which has been associated with perceived neighbourhood safety, and behaviours such as walking and cycling. This may include having local programs such as Neighbourhood Watch, or ongoing events and programs that facilitate social interaction between local residents, and walking and cycling.

These social aspects are further supported by some built environment and health evidence connecting the built environment with social interactions which, in turn, facilitate social capital (114-116). For instance, more walkable neighbourhoods that are characterized by more connected streets, higher number of residential dwellings, and a greater mix of local destinations, have been found to increase social interactions as people are more likely to walk, cycle, and linger locally (115).

3.12 Historical events

3.12.1 Relationship with ECD

Historical events *Leaders respond to events in ways that bring local community members together to create a shared storyline or engage in activities of citizenship*

Historical events or factors relate to the KiCS governance domain, in that it can shape local governance processes.

The capability approach maintains political wisdom and capacity to contribute are built through the process of participation.[117] When disadvantaged communities harness participation through popular movements, local people can be agents of change.[118, 119] Responses to events and/or social and economic shocks may reveal local leaders and contribute to development of a "broad and inclusive storyline for the community".[120] Events may produce the motivation for participation[121] as well as prompt opportunity for informal connection and discussion between community members about their shared interest in the event. In this way, historical events signal the response of leaders to particular events that can bring community members together to engage in activities of citizenship and foster collective identity. This, in turn, can create supportive local social and governance processes that influence ECD outcomes.

3.12.2 What KiCS found

In KiCS communities, historical factors were associated with a stronger sense of place (or sense of community). A stronger response from leaders to bring community members together was found in disadvantaged communities where children were doing better than expected, compared with disadvantaged communities where children were not doing as well. Events themselves may be positive or negative, the influence is in the potential for leaders to respond to events in a way that formulates a shared local narrative and contributes to citizenship activity. Some examples of historical events which were associated with particular responses by the community included the amalgamation of different local government areas, natural disasters, or a tragedy within a community. There was some evidence that responses take time to mature into a shared narrative and citizen activity and thus have an impact on the community. In some examples, the response to an event or shock appeared to stall rather than mobilise action. For example, one KiCS suburb had been included on a prominent list of most disadvantaged suburbs and seemed unable to find the leadership and coherent storyline to translate the resulting investment into meaningful improvements in ECD outcomes.

To illustrate, one KiCS community that was doing better than expected in ECD scores had been forced to amalgamate with a neighbouring local government area. In response, the community campaigned against the amalgamation, giving rise to local leadership and fostering a shared identity. Decades later, the collective identity of the community is the basis of a strong sense of pride within the community, despite external perceptions of stigma. Such phenomena has been described as "oppositional consciousness" where groups treated as subordinate recast their situation with a "positive identification".[122]

> ***"It's because about 20 years ago, all these different areas had their own local government. And 20 years ago, there was an amalgamation. So that all these different townships were then brought [together and] lost that sense of identity in, in terms of their government or structure... But kept that sense of identity in terms of within the community."*** (INT005)

HISTORICAL EVENTS	What are we measuring?	How we measured it
Qualitative	Response of leaders to events that bring local community members together	Interviews and focus groups

The FCF of historical events was measured qualitatively and acts as an opportunity for leaders to respond in a way that brings local community members together. As such this factor explores the response of local leaders rather than the historical events themselves.

Examples:

- Responses to events and/or social and economic shocks may reveal local leaders and contribute to development of a "broad and inclusive storyline for the community".[120] Events may produce the motivation for participation [121] as well as prompt opportunity for informal connection and discussion between community members about their shared interest in the event.
- Being included in a list of top most disadvantaged postcodes and subject to a wide range of uncoordinated investment in programs creating confusion and overlap rather than a coherent storyline. Joined up work may need to develop over time and focus on both "purpose" and "context" – i.e. needs to be specific to the situation.[123] This factor may account for the failure of investment in response to events or communities.

3.12.3 Limitations with measurement and use

While historical identities cannot be replicated, contemporary efforts can act to develop a collective identity and local participation based on issues of interest to the community, as well as promote positive, rather than negative, responses within the community.

Some other caveats to the FCF of historical events to consider:

- Informal local community groups and/or cross-cutting initiatives can actively work to improve social cohesion, foster social capital and community engagement, strengthen accountability and support universal services. This is closely related to findings for the KiCS social domain. It may also be linked with other FCFs listed (e.g. Stigma)
- Joined up work needs to focus on both "purpose" and "context" – that is, needs to specialise according to the situation and time may be needed for a local event to mature into a coherent community narrative.[123]

3.13 Local decision-making

3.13.1 Relationship with ECD

***Local decision-making** is associated with 'novel approaches' or locally tailored initiatives or solutions (including any with a focus on social capital) that have been developed and implemented in the community*

A rationale for local governance is that solutions to "wicked problems" – problems that defy simple solutions[124] – are best derived from the lived experiences of those affected by the problems. Thus, collaborative and networked place-based partnerships between government, private and community sectors have become conventional in Australian ECD policy.[125-128] The fundamental factor driving participatory governance is the idea that the contribution of those that experience problems and challenges is necessary to generate appropriate solutions. Cornwall and Coehelo differentiate between community participation that is 'invited', arising within governments and institutions; and 'popular', arising from movements within the communities themselves.[129] However there is a concern, particularly for disadvantaged communities, that those with most to benefit may be least equipped to participate in shared governance approaches which have been criticised for reproducing the social structures they are intended to tackle.[119, 121, 130-132]

Evidence points to the significant influence that local leaders, champions or convenors can have on public policy and neighbourhood outcomes and neighbourhood outcomes in initiatives that arise as a result of popular movements.[133, 134] When individuals play influential roles, they may be referred to as connectors or "boundary spanners",[135] and they may further "animate" or "enable" local participation and decision-making and thus influence ECD outcomes.[136] Local decision-making that is specific and sensitive to the local context and addresses priorities identified by local people can produce tailored initiatives and meaningful outcomes for local people, resources, and the environment.[137]

3.13.2 What KiCS found

Local decision-making	What to measure	How to measure it
Qualitative	Development of novel approaches or locally-tailored initiatives or solutions as a result of local decision-making	Interviews and focus groups

This FCF explores the development of locally-tailored initiatives or solutions as a result of local decision-making. This FCF is measured qualitatively through interviews and focus groups. Locally-tailored approaches spur the development of bridging capital, where citizens are able to convert social capital into meaningful ways to influence local decision-making.[138]

Examples:

- Perceived crime and youth 'gang' activity tackled in a sensitive, inclusive and integrated way.
- Locally-based groups actively working on social cohesion or engaging the community in tailored initiatives or solutions (e.g. lobbying for community infrastructure).

3.13.3 Limitations

- This FCF is linked closely with other governance domain FCFs such as leadership, which has been found to be an important factor in communities
- This FCF may also be related to the **Historical events** FCF.

More research on 'bottom-up' institutions and if and how neighbourhood movements interact with 'government sponsored instruments' needs to be encouraged.[136]

PART 4 **Important FCFs**

In Stage 2 of data analysis (see **Section 1.4.2**), we embarked on exploring factors that appeared important for ECD, regardless of whether the community was 'doing well' or 'doing poorly' on ECD despite its socioeconomic status. The important FCFs are the community-level factors that are consistently important across the majority (at least 16) of the 25 local communities in KiCS. This analysis focused on exploring qualitative data within and across communities (with a view to explore any consistent trends). An important FCF may also be a differentiating FCF. Based on qualitative data only, eight community factors for ECD have emerged as important, and three have emerged as encouraging but the analysis is still yet to be completed. **Table 10** summarises the Important FCFs, examples from KiCS local communities, and the links to ECD literature.

This section does not attempt to discuss each important FCF individually (unlike the differentiating factors). Also, this section is not covered in the KiCS FCF Manual as further research is required to identify the best ways communities can effectively implement the important FCFs locally. Rather, this section discusses our key learnings, and how the factors are interrelated to either indirectly or directly influencing young children's outcomes.

Table 10. Important FCFs for ECD

	Important Foundational Community Factor	Description	Example/s in KICS	Potential links to ECD
1	Physical access to services	Reported instances of ability to get to services	Walkability (physical domain) was important for some communities, while for other communities walkability was not a facilitator to service access (e.g. too hot to walk, too hilly, high proportion commuting by car).<br>Other examples include: access to public transport (physical domain), co-location of services to shopping centres, childcare services includes bus pick-up and drop-off	Physical accessibility is important for service provision and use, particularly for young families who need access to essential services. In communities where children's services are lacking and not available, detrimental ECD outcomes are common.[(139)] Yet while services may be available, a significant barrier to their use is if families do not have the ability to physically get there, due to need to travel (distance), reliance on public or private transport, traffic, poor walkability, weather conditions or geographical location. As such, this important factor relates to other important FCFs described here including *walkability, public transport availability and traffic exposure.*<br>Neighbourhood effects research suggests that services located with other amenities (co-location) of daily routine activities can support children's well-being.[18] For example, the presence of services with shops, stores, public transport can influence how families use the services whilst attending to other daily errands with their children.

	Important Foundational Community Factor	Description	Example/s in KICS	Potential links to ECD
2	Walkability	Walkability to facilities and services was seen as important to get to places	• 17 of 25 local communities<br>• Walkability was important particularly when families who did not have a car and public transport was limited<br>• Walkability also was discussed relating to the ability to walk safely from traffic, with a pram and young children, lack of footpaths	Walkable environments (ease of walking locally) is beneficial to health and wellbeing.(140) Having destinations and services within a walkable distance (e.g. 800m for older children, 1600m for adults) increases the likelihood of walking and use of the destination.(141-144) For young families, this may affect access to essential services (given amenities and services are available locally). If places are not available locally, there may be negative effects on families with young children, if *walking* is the primary transport mode e.g. 'Forced' transport walking may affect young mothers with children through physical fatigue, pressures of managing children who are tired from walking long distances, and lack of motorised transport confining mothers and children to local areas which may or may not provide all the amenities and services they need.(145)
3	Public transport availability	Presence of/ access to public transport was seen as important for easy access within the suburb	• 20 of 25 local communities<br>• People wanted to take public transport<br>• Public transport stops not within walking distance<br>• Lack of available public transport options (train, bus)<br>• Poor synchronisation of bus timetables and school/kinder drop off making it an all-day round trip for parents, which is not feasible.<br>• Bus routes changing so they do not provide easy accessibility to schools, ECEC, work.	Public transport (PT) within a community may have important benefits for community residents and families, which may indirectly affect children's outcomes. Access to PT also provides families increased access to numerous health enhancing services and resources that may not be walking distance, or located outside their local community (e.g. health services, education facilities, food shops etc.).(146)<br>Lack of PT is a large part of transport disadvantage.(145) Availability of PT provides individuals with increased access to employment opportunities, increasing family wealth/income.(146) For young mothers who rely on PT services, difficulties in access may result in significant barriers to other places and services, limiting social networks and local community participation.(145) Fritz (2007) found that physical inaccessibility proved to be the most significant barrier, with non-family friendly policies such as having to fold prams before boarding PT, and the lack of assistance contributing to this.(145)
4	Traffic exposure	Being away from traffic within the suburb is an important factor for children being safe	• 16 of 25 local communities<br>• Limits young children's play opportunities<br>• Limits access to the outdoors (e.g. busy roads deterring access to places)<br>• If driving, being stuck in traffic is frustrating and stressful for parents and children can see that (role modelling)	Low traffic exposure is a key factor in neighbourhood safety.(34) Parents view increased traffic, speed and poor availability and design of crossings as high risks to child safety. This influences parent's decisions to allow their children to use parks, and play in their surrounding environments.(34, 147) A review of the literature found there is increased injury in children when exposed to increase traffic with limited traffic calming measures, e.g. sidewalks, street crossing, side street parking, traffic lights.(148)

	Important Foundational Community Factor	Description	Example/s in KICS	Potential links to ECD
5	Public open space – availability and quality	Having parks in the suburb was seen as important for young children and families<br>Having good quality parks was seen as important for use, play/social interaction	• 19 of 25 local communities<br>• Important destination for children's play<br>• Parks with high quality playgrounds, seating, toilets available etc. are important<br>• Poorly maintained parks (graffiti, perceived crime, and incivilities, rubbish) can deter use	Having quality public open spaces available, such as parks, can strengthen family relationships and provide children safe green environments to play.[149] This increases their participation in different types of physical activity, improving motor skills, and social development. [150-152] Exposure to green space and physical activity have also been associated with enhanced emotional wellbeing in children.[150, 151]<br>Perceived high quality public open space may facilitate social interaction and create a sense of community.[153] A study found a significant association between accessibility and use of public open space suggesting that if there were increased availability to green open space within communities, it would increase residents' use.[154]
6	Facilities - availability and diversity	Having a range of family-friendly destinations and activities is important for young families and children	• 22 of 25 local communities<br>• Free events<br>• Range of activities (e.g. sporting grounds, swimming pools, shopping centres) being available locally	Having a range of places and destinations available locally can influence use and sense of community. Designing neighbourhoods that encourage children's health and wellbeing includes access to local destinations- i.e. green space and nature, and local infrastructure and services.[85, 151] We know that destinations such as schools, recreation venues, and child and health care services all have inherent functional roles, but they can also serve as physical places for social interaction and developing networks of support.[155] These places may influence children's development through providing opportunities to learn, explore, recreate, socialise, and interact.[156, 157] Close proximity to local destinations is an important component of destination accessibility.
7	ECEC Cost	The affordability of ECEC is considered important and affects use	ECEC was consistently considered an important factor across KiCS local communities and seen as unaffordable (a concern) for most 16 of 25 local communities.<br>A key barrier cited was related to government funding changes and class ratio requirements. Often government subsidies for childcare had the primary focus of increasing participation in parents' employment and study, however many families without the human capital (e.g. confidence, exposure, skills) to successfully complete study/work missed out on subsidies. Conversely, there were positive reports of state/federal government policy in improving universal access to kinder.	It is well documented that ECEC can benefit and promote positive ECD outcomes.[97] Since 2009, there has been increasing focus on Early Childhood Education and Care policy, which led to the introduction of the National Quality Framework for ECEC in 2012.[158] Federal commitment to universal access of 15 hours a week (600 hours a year) of kinder/preschool for children the year before their first year of primary school has been supplemented with initiatives for vulnerable populations, as well as initiatives to increase parents' participation in employment.<br>The balance is in providing an operationally viable service whilst adhering to laws and regulations to ensure the provision of quality ECEC. As such the costs of ECEC can be a barrier for families to use services.[158, 159]

	Important Foundational Community Factor	Description	Example/s in KICS	Potential links to ECD
8	Leadership	The presence of local champions, leaders and boundary spanners driving local governance	• People hold multiple positions in voluntary, community and local government roles.<br>• Several community activists hold specialised knowledge and traverse several fields in their involvement.<br>• Local member plays a key role in driving or interpreting local policy.<br>• Leaders who have been part of the community for a long time may have cultural capital and networks that can be used to advance local agendas.	Governance literature emphasises the influence of local leaders, champions or convenors on public policy and neighbourhood outcomes.[133, 135, 136, 160-162] A challenge for qualitative network governance research is to understand who is in charge and how leadership is practiced in local contexts.[163] In these contexts, power is exercised at both macro, meso and micro levels and research needs to explore the mix of local or devolved leadership, the bridging between local institutions and higher order structures (i.e. state and federal agencies) and state leadership and its institutional requirements for participatory governance.[128]<br>Similarly, with the FCF of *local decision-making*, this FCF can work distally through local governance activities to support ECD as well as provide direct opportunities for children to have positive experiences.
**ENCOURAGING IMPORTANT FACTOR - Analysis incomplete**				
9	Service Co-ordination	Co-ordination of services in a local community	Service coordination is evidenced in KiCS to varying degrees from a general understanding of services to formal partnerships that support coordination. Examples in KiCS:<br>• Co-location of ECEC and schools (facilitates linkage) was found to work in some communities, and less effective in other communities.<br>• Having a specific referral service for Child and Family services across a region (linkage)<br>• Interagency networks or partnerships of early childhood services (coordination)<br>• Co-location with 'routine activities' or other services	Service coordination refers to the existence of structures and mechanisms to assist in the transition of services throughout the life course (e.g. from kindergarten to school) such as partnerships or processes of linkage to other services.[11, 164, 165]<br>Service coordination refers to:[166]<br>1. Linkage of services<br>2. Coordination<br>3. Full integration<br>And also Co-location (with other services or facilities)<br>For KiCS the main area of service coordination that appeared important was the ability to facilitate 'warm/soft referrals' for families into relevant services. This encompasses building the trust of service providers to support their needs enabling intervention.
10	Sense of community	Reported strong neighbourhood attachment or sense of belonging and pride in being connected to a local community	Some local communities demonstrated a strong sense of belonging, and collective identity, particularly when external perceptions of the community were negative.	Sense of community can be referred to under the umbrella term 'social capital'. Strong social capital within a community fosters trust, increased social interaction amongst neighbours, which can lead to positive support for young children.[18] Positive social capital is associated with higher behavioural scores in children, reduces school dropout rates, improves mental health outcomes and improves overall wellbeing in children.[167]<br>One study found a significant association between community belonging and childhood outcomes.[168] Parents who had high sense of belonging in their community, were linked to decreases in hyperactivity, emotional symptoms and social problems in children.[168]

	Important Foundational Community Factor	Description	Example/s in KICS	Potential links to ECD
11	Natural environments	Natural spaces are seen as important for young families	Natural spaces seen as 'attractive' and more likely to facilitate use, leading to play and social interaction for young children	See *family-friendly destinations and parks and green spaces.*<br>Natural play environments (with elements such as trees, wood, and flowers) appear better for children's cognitive and physical development than physical 'man-made' play areas.[169] Children who play in natural areas engage in more physically demanding play, demonstrate better gross-motor skills (e.g., climbing, balance, coordination), have increased attention spans, and fewer sick days at day care centres compared with children who play in purpose-built playgrounds.[170]

While there are a number of important FCFs, our findings (in the qualitative data at least), highlight that:

- Not all FCFs are important for every community. The FCFs may influence ECD differently depending on community context;
- The FCFs do not necessarily work in isolation. They may be related to each other (e.g. common themes or 'groups');
- The FCFs may influence each other along the pathway to influence ECD;
- The pathways and mechanisms by which communities influence ECD are complex.

The FCFs provide a draft set of community-level factors that may signal areas of influence on ECD (i.e. 'the what'). They also provide some ideas on 'the how' and 'why' it may influence ECD, however, further research on 'how much' of an influence on ECD is needed.

Example:

The physical domain FCFs are related to each other, and they also relate to other domains (e.g. service and social). The main themes that have emerged reflect the importance of having local services and destinations nearby (family-friendly destinations such as parks, and essential services), but more importantly, having transport options to 'get there' (e.g. walkable environments and public transport). Physical access to key services (e.g. child care, primary schools) and destinations (e.g. shopping centres, parks) are a main barrier to access to use as illustrated by our local communities:

> ***"Funnily enough again, if you spoke to someone in Council, they'd say, "Aw. There's everything there. There's banks, there's gyms, there's this, there's that"... but only if you can get there"*** (FG09)

> ***"It can be very isolating and as we actually see the women arriving very exhausted by the time they get here, they've walked for 45 minutes to attend a two hour women's group....It's a lot"*** (INT072)

> ***"If there's no bus and we've got no transport, families won't use the service"***

PART 5 **Discussion**

We set out to find community-level factors associated with ECD. In doing so, we explored community factors that seemed to explain differences between disadvantaged local communities with better ECD outcomes than their neighbouring local community, despite both being socioeconomically disadvantaged. The suite of *differentiating* FCFs that appeared to be protective of ECD in disadvantaged communities are mostly related to socio-demographic, local governance, and social domains. Currently, there are very few physical and service differentiating factors. However, further analysis of the qualitative data shows that there are more physical, service and governance factors perceived to be important for early childhood by many communities.

Relationships between the community factors, families and children are complex, with no ‘one-size fits all’ or ‘magic bullet’ approach to improving outcomes for young children. We know that there is not one community factor that will make the difference to ECD. From the KiCS findings and previous literature, it is unlikely that all the FCFs will have a *direct* influence on ECD. While socio-ecological frameworks include the neighbourhood or community as an important environment in which children grow and develop, it is the most distal environment of influence in these frameworks,(17) meaning that we would not expect overly large effects on ECD, and it is less likely to directly influence ECD compared with more proximate environments such as the family and home environments.(171) But we know that families exist in relation to other resources and opportunities within the community.(40) Neighbourhood conditions may contribute to development at young ages, because of *indirect* effects on parent behaviour and perceptions, and because it affects the whole population.(171) That is, the community or neighbourhood provides the conditions that help facilitate or hinder family lifestyle choices and behaviours, which in turn, impact on children’s health and development. As we have shown through KiCS findings, communities provide the structures, conditions and resources that are conducive for families with young children to thrive. This could be through:

- Facilitating or hindering families’ use of, and access to local resources
- Influencing parent and service provider perceptions of different community groups, and of themselves as a community
- Influencing how communities work together towards better collective outcomes

The FCFs presented here appear to be related to each other (explained throughout **Part 3**); socio-demographic FCFs still play an important role despite attempts to control for SES through the selection of on- and off-diagonal communities e.g. both neighbouring local communities experience disadvantage with one having better ECD outcomes than the another. In KiCS, it has been difficult to completely ‘tease apart’ the mechanisms in which communities make a difference to ECD, and which factors may be ‘more important’ than others. Others have tried to quantify and model the relationships between community-level effects and ECD with quantitative data, and isolate which community factors exert a larger ‘difference’ on ECD.(40) Such work has a long way to go. With further research, more robust evidence on the pathways (how and why) in which community factors (what) influence ECD, and at what scale (how much) will make a difference to ECD.

While there are interdependencies between factors and caveats with the data, we have attempted to explain the FCFs associated with ECD, rather than explore whether there was a causal link between community-level factors and ECD (e.g. if you have higher income, you have better ECD scores). The FCFs were developed to help communities understand the community-level factors that might be associated (rather than lead to) relatively better early childhood development. These FCFs may signal:

- potential points of intervention communities may want to consider
- understand potential reasons why ECD outcomes are poorer; or better in their community

Example:

Using housing tenure as an example, we cannot conclude that having lots of renters in your suburb causes poorer ECD. Living in a suburb with fewer renters may be associated with better child development in and of itself because it limits residential mobility (transience), but it may also be that suburbs with higher rates of home ownership are richer than suburbs with lower rates of home ownership, and it is that rather than rental status that is driving development. We can say that this is an area worth investigating further in a community.

5.1 Strengths

Exploring factors that may be modified 'at scale' (i.e. has the potential to impact a large population, including young families and children) is warranted. The combination of family and community, working in synergy, powerfully determines children's outcomes. While quality early life experiences are important for all children, they have been shown to be particularly vital to overcoming the effects of disadvantage.[98] The differentiating FCFs provide potential focus areas that disadvantaged communities can consider to improve ECD outcomes.

KiCS faced the challenge of collecting and integrating a mix of qualitative and quantitative methods to explore community-level influences. Investigating community-level factors associated with ECD is a relatively unexplored area of research compared with family, school and individual factors. Beyond ECD research, there is a wealth of research exploring community influences on other child health behaviours and outcomes. Guided by previous literature and our conceptual framework, we had both quantitative and qualitative data to provide a better understanding of the local context for different types of local communities, and the community factors that are important for early childhood within these communities. It is this complexity that is a major strength of the study. From the qualitative information, in particular, we reiterate that every community is different, and the FCFs can be used as a starting point for communities to discuss and prioritise what may be important for their local context. For example, a particular FCF may not be important or of high priority for a community. However, other FCFs may take first priority. Applying what makes sense for the community context, and identifying their priorities and resources, are important conversations to have locally.

5.2 Limitations

5.2.1 Challenges with measurement

a. Quantitative FCFs and indicators

Quantitative data (e.g. ABS Census, service information, community survey, and GIS data) were sourced for a relatively small number of local communities. This presents challenges in the representativeness of results and ability to conduct further statistical modelling. As we have so little data on the individual children in the AEDC, we were unable to move beyond simple descriptives to further explore any relationships between community factors measured quantitatively, and the AEDC scores of children. This means we cannot definitively conclude that each factor causes AEDC outcomes, or that one community factor, in particular, affects the AEDC.

While there is limited capacity to conduct further quantitative analyses beyond descriptives, best efforts were undertaken to source and triangulate quantitative data that may help explain any emergent qualitative themes. Given this limitation, our FCFs and indicators recommended quantitative measurement should be used carefully for context purposes only. While the ABS Census information is reliable for use, our analysis is based on the selection of on- and off-diagonal local communities, which takes into account the local community's SES and children's vulnerability on one or more domains on the AEDC i.e. AEDC DV1 scores. The AEDC is collected every three years and different children are included each time. This means the AEDC DV1 outcome (hence 'diagonality' of local communities) may vary over time. This means we cannot truly say for example, that changing housing tenure influences ECD. This limits our ability to draw more robust conclusions making it difficult to have confidence in the quantitative results.

Community survey: The community survey did not yield a representative sample (e.g. high proportion of older adults) or a high response rate (i.e. 16.2%), and thus was *not* recommended for further consideration, nor as a method in the KiCS FCF Manual. Future research should explore better ways to collect surveys from local residents.

Service information: Similar to the community survey, service surveys were unsuccessful ways of data collection. Parent surveys were initially trialled but dropped as a method early in data collection due to difficulties in recruitment. While service providers participated in the focus groups, low response rates to the service surveys may mean that a more targeted approach (e.g. tailored to service type) to collecting information from parents and service providers about perceived service availability, access, quality, coordination, and use, is needed.

GIS data - Geographic boundary and scale: Built environment GIS measures were calculated at the 'local community' (suburb) level for descriptive purposes; it is the smallest spatial unit that the AEDC data are publicly available. However, finer resolution data (i.e. smaller spatial unit data) are generally considered as more appropriate for studying neighbourhood effects.[172] Not only can they be aggregated to larger spatial units,[173] but they also capture more spatial heterogeneity, including, for example, the flexibility to identify areas with poorer access to parks. Indeed, it has been shown that in many instances the 'local' more proximate neighbourhood appears a better predictor of an individual's behaviour, rather than regional- or city- level attributes.[174] Previous studies suggest that the choice of spatial unit is likely to generate different results depending what the GIS measure is, potentially leading to measurement (aggregation) errors, and attenuate associations with health outcomes.[175] Thus, the choice of the spatial unit to represent the 'neighbourhood' is important and has implications on the magnitude of associations.[176]

GIS data - urban measures applied to regional areas: GIS data, while a useful analytic tool in examining community-level effects are currently mainly available only for urban environments, not regional areas. For example, 'walkability' is conceptualised from an urban perspective, it is not expected that regional towns have the infrastructure or population to support walkable attributes such as street connectivity and residential density. Broader utility for use of GIS in regional communities is yet to be assessed.

b. Qualitative data

Reporting bias associated with qualitative data may have occurred. For example:

- Selection of local communities was based on 2012 AEDC data, but community investigations commenced in 2015. This is a limitation because communities may have changed during that time gap, and residents, service providers or stakeholders may not have recalled what their communities were like in 2012.
- In focus groups, participants may be more likely to discuss what they are exposed to in the community (e.g. physical environment, services they use). Nevertheless, the semi-structured nature of the focus groups did allow for prompts for other domains.

5.2.2 Generalisability

These FCFs are derived from *disadvantaged* neighbourhoods only, and may not be generalisable to more advantaged neighbourhoods. The issues of scope and generalisability are a critical consideration for studies of this nature. While we tried to identify consistent community-level factors associated with ECD, the intricacies of different community contexts also limit generalisability.

Not all neighbourhoods are the same, and there are often more differences within rather than between neighbourhoods.(17, 18) Disadvantaged neighbourhoods vary in terms of risk factors (e.g. crime rates, neighbourhood safety) and protective factors (e.g. social capital, collective efficacy). These FCFs are measured at the suburb-level. We know that there are 'pockets of dis/advantage' within each community. For example, if you live close to the main town centre, you may have better public transport access than people living further away. People may talk about particular areas or places of concern, yet factors measured at the suburb-level may not necessarily capture these details (e.g. limiting the specificity of GIS data). Also, there are differences between sub-groups (e.g. cultural groups) within the community and the FCFs may likely perform differently across different groups.(177) Further research is needed to capture differences within neighbourhoods to identify opportunities to flatten inequities.

PART 6 **Conclusion**

The Kids in Communities Study (KiCS) explored community factors that make a difference for young children in 25 local communities of advantage and disadvantage across Australia (ECD). We looked at a range of factors (measured qualitatively and quantitatively) in local communities where children were doing well or better on ECD (using the Australian Early Development Census (AEDC)) relative to their socio-economic status (SES) (i.e. **differentiating FCFs**). But we also investigated what communities perceived were important for families with young children (i.e. **important FCFs**). Together these factors make up a list of *Foundational Community Factors* (FCFs): the factors that lay the foundations for a good community for young children (**see Figure 3**). The list of FCFs signal areas of focus for ECD, rather than providing robust community indicators of ECD. Having local information on the FCFs can assist communities in better understanding what facilitates or hinders ECD at the community level, recognise their resources and opportunities to improve, thus helping to direct community effort into areas that make the most sense.

6.1 The beginning of FCFs for ECD… not the end

The list of FCFs (and its measurement) may be added to in the future, pending further testing and research. In the interim, the FCFs contributes to the evidence base on community-level influences on ECD. The mixed methodologies in this study help to converge and validate qualitative and quantitative findings to provide a more in-depth understanding of the community factors associated with ECD outcomes in communities. In a world of practicalities where the rhetoric for community change has outpaced the evidence; it is important to insert some level of measurement rigour that both facilitates the exploration of community understanding and suggests (rather than proves) some level of causal link. With increasing interest in "place-based" strategies from governments and philanthropic agencies globally, the availability of rigorous approaches to data collection and FCFs, underpinned by theory and tested for associations with ECD outcomes, is limited.

The draft FCFs can be used to:

- Identify community areas of concern or interest, strengths and weaknesses, thus directing local efforts into place-based initiatives that make the most sense.
- Advocate for areas of most concern for different community groups and identify ways in which communities can respond locally.
- Move beyond anecdotal information to a discussion grounded in evidence about how the community is tracking to inform place-based initiatives.[13]
- If the FCF is recommended as an 'indicator', benchmarking or monitoring community progress over time can work towards providing communities that are better for children and families and likely to promote ECD.

To help with local measurement, the supplementary **KiCS FCF Manual** suggests measures and methods communities can use to collect the differentiating FCFs themselves. It makes an important contribution to help communities and governments measure and drive local efforts toward better and more equitable communities in which young children live and grow.

The important FCFs are currently *not* recommended for measurement in the KiCS FCF Manual. These FCFs are still considered important for ECD, however further testing is needed to explore the appetite of communities for collecting a large amount of qualitative data. Further research on the important FCFs can help to test stronger and more specific quantitative measures and develop more targeted interview or focus group questions (rather than semi-structured) for communities to consider when measuring each FCF.

a. Next steps

The mix of qualitative and quantitative data provided a 'deep-dive' into 25 local communities. The findings resulted in a list of promising FCFs that can be measured quantitatively and/or qualitatively. While we have developed a list of FCFs, there are two ways to upscale the FCFs to achieve both depth and breadth of impact. Future research should involve taking innovation to scale through progressing a set of measures that will have ultimate utility in place-based efforts:

1. We found that there is no 'one-size fits' all and no matter what, understanding the needs and context of the community is necessary. Local measurement of the FCFs requires 'road testing' the Foundational Community Factors (FCFs). This means: 1) testing the utility of the draft **KiCS FCF Manual** of methods and measures in different local communities; and: 2) co-designing an approach to measurement that works at the local level. Co-designing and piloting a feasible approach for local implementation will ensure better community 'buy-in' and accelerate impact, helping to direct effort into areas that make the most sense. Taking measurement to scale can be time consuming and complex, and requires community 'buy-in' in the future. Thus the ideas, measures, and methods recommended here and in the **KiCS FCF Manual** need to be 'road tested' with a sample of communities to explore what's possible.
2. While localised community data can help inform in-depth local place-based efforts, moving the research to scale provides breadth of impact. This could mean: 1) testing stronger and more context-specific quantitative measures; 2) developing more targeted interview or focus group questions (rather than semi-structured questions) for communities to consider when measuring each FCF; and 3) further research to develop KiCS qualitative FCFs into quantitative measures, and eventually indicators.

Example:

Having both quantitative and qualitative data provided a better understanding of the local context, but there were limitations with the quantitative data. The quantitative data (e.g. GIS built environment data) was sourced for a relatively small number of local communities. This presents considerable challenges in the representativeness of results and ability to conduct further statistical modelling. As such, this limited our ability to develop robust quantitative indicators.

Exploring the influence of the neighbourhood built environment on ECD is possible through data linkage opportunities. GIS measures of the built environment can be linked and explored with ECD data from Australian Early Development Census (AEDC), which captures approximately 98.5% of Australian five-year olds. Such work will provide the first large-scale evidence base on built environment 'neighbourhood effects' on ECD.

PART 7 Appendices

7.1 Appendix 1. Full suite of community factors explored

Potential FCF		Factor/Theme/Hypothesis	Data type
**Socio-economic domain**			
Cultural diversity	1	**Cultural diversity**<br>Level of cultural diversity is greater in Off+ (or OnAdv) More in OnDis	Qual
	2	**Aboriginal and Torres Strait Islander**<br>Proportion of residents identifying as Aboriginal, Torres Strait Islander or both is less in Off+ (or OnAdv)	ABS Census
	3	**English Proficiency**<br>Off+ (or OnAdv) has higher proportion speaking English only	ABS Census
	4	**Language diversity**<br>Proportion of adults aged 25-54 who speak a language other than English at home is greater in Off+ (or OnDis)	ABS Census
	5	**Arrived in last 10 years**<br>Proportion of residents arrived in last 10 years (2006-2016) is less in Off+ than OnDis	ABS Census
	6	**People from similar backgrounds**<br>There is a higher proportion of people in Off+ who perceive others in the neighbourhood to be from similar backgrounds	Community survey
Income	7	**Higher income**<br>Level of economic diversity is greater in Off+ (or OnAdv)	Qual
	8	**Median weekly household income**<br>Median household income is higher in Off+ (or OnAdv)	ABS Census
	9	**Household Income**<br>Off+ (or OnAdv) has a higher proportion of residents aged 25-54 with equivalised household weekly incomes greater than $2000/week	ABS Census
Age diversity	10	**Mix of younger and older people**<br>Level of age diversity is greater in Off+ (or OnAdv)	Qual
	11	**Residents aged 65-84 years**<br>Higher proportion of residents aged 65-84 years in Off+ (or OnAdv)	ABS Census
Housing affordability	12	**Housing affordability**<br>Housing affordability is becoming more of an issue in the Off+ than OnDis: Higher SES families are moving into the area and displacing more disadvantaged groups	Qual
	13	**Housing affordability**<br>Housing in Off+ is more desirable leading to less affordable housing in OnDis	Qual
	14	**Housing affordability**<br>In OnDis, A higher proportion of lower income households (lower 40%) are paying more than 30% of their income on housing costs	ABS Census
Housing Tenure	15	**Home ownership**<br>Lower proportion of renters in Off+ (or OnAdv) than on-diagonal	ABS Census

Potential FCF		Factor/Theme/Hypothesis	Data type
Public housing	16	**Public housing**<br>Perceived less public housing in Off+ (or OnAdv) than on-diagonal	Qual
	17	**Public housing**<br>Lower proportion of public renters in Off+ (or OnAdv) than on-diagonal	ABS Census
Highest level of schooling	18	**Highest level of schooling**<br>Off+ (or OnAdv) had higher levels of Year 12 than on-diagonal	ABS Census
University degree (aged 25 to 54)	19	**University degree**<br>Off+ (or OnAdv) had higher proportion of residents aged 25-54 with a university degree	ABS Census
Working as managers or professionals (aged 25 to 54)	20	**Managers and professionals**<br>Off+ (or OnAdv) had high proportion of residents aged 25-54 years working as managers or professionals	ABS Census
**Physical domain**			
Walkability	21	**Perceived walkability**<br>Perceived walkability to facilities is better in Off+ is better than OnDis	Qual
	22	**Perceived walkability**<br>Perceived walkability to services is better in Off+ LCs than OnDis	Qual
	23	**Walkability index**<br>Walkability Index Score of local community is higher in Off+ than OnDis	GIS
Access to facilities and services	24	**Perceived service availability**<br>There are more perceived services available in Off+ than OnDis	Qual
	25	**Family destinations**<br>More family destinations in Off+ than OnDis	GIS
Quality of facilities	26	**Perceived quality of facilities**<br>Quality of facilities and places is better in Off+ than OnDis	Qual
	27	**Perceived quality of services**<br>A higher proportion of residents perceive there are better quality services in Off+ than OnDis	Community survey
Park availability and access	28	**Perceived number of parks**<br>Perceived number of parks is higher in Off+ than OnDis	Qual
	29	**Number of parks**<br>More parks in Off+ than OnDis	GIS
	30	**Distance to closest park**<br>On average, parks in Off+ are closer than OnDis (shortest distance (m) to park)	GIS
Park quality	31	**Perceived quality of public open space and parks**<br>Quality of POS and parks is perceived to be better in Off+ (or OnAdv)	Qual
	32	**Perceived quality of parks**<br>A higher proportion of residents perceive there are better quality local parks in Off+ (or OnAdv)	Community survey
	33	**Average park attractiveness**<br>Average public open space attractiveness score is greater in Off+ (or OnAdv)	GIS
	34	**Most attractive park**<br>Off+ (or OnAdv) has a higher Most attractive park score	GIS

Potential FCF		Factor/Theme/Hypothesis	Data type
Public transport	35	**Perceived public transport access and availability**<br>PT access and availability is better in Off+ (or OnAdv)	Qual
	36	**Distance to closest public transport stop**<br>Distance (access) to PT is shorter in Off+ (or OnAdv)	GIS
	37	**Proportion living within public transport**<br>A higher proportion of Off+ (or OnAdv) is within a PT stop (400m bus stop/600m tram/800m train or ferry)	GIS
Housing	38	**Perceived higher-rise density housing**<br>Perceived higher housing density in OnDis than Off+ (this refers to number of storeys)	Qual
	39	**High-rise density housing**<br>There is a lower proportion of high density housing (3 or more storeys) in Off+ (or OnAdv)	GIS
Housing type	40	**Public housing type**<br>Compared with OnDis, there are more public housing classified as separate houses in Off+ compared with public housing classified as town houses/ apartments	Qual
Traffic	41	**Perceived traffic exposure**<br>Lower perceived exposure to traffic in Off+ (or OnAdv)	Qual
	42	**Perceived traffic exposure**<br>Perceived lower traffic (TrafficSafety score) in Off+ than OnDis	Community survey
	43	**Traffic exposure**<br>Lower traffic exposure in Off+ (or OnAdv)	GIS
**Social domain**			
Stigma	44	**Stigma**<br>Stigma is greater in OnDis (or Off-)	Qual
Sense of community	45	**Perceived sense of community**<br>Sense of community (a different issue to stigma) is more evident in Off+ (or OnAdv)	Qual
	46	**Sense of community scale**<br>There is perceived better sense of community in Off+ (or OnAdv)	Community survey
Crime	47	**Perceived crime**<br>Perceived crime is lower in Off+ (or OnAdv)	Qual
	48	**Crime safety scale**<br>Perceived crime safety is better in Off+ (or OnAdv)	Community survey
	49	**Crime rates**<br>Lower rates of crime against person in Off+ (or OnAdv)	Crime rates
Social capital - bonding	50	**Perceived bonding social capital**<br>Bonding capital is more evidence in Off+ (or OnAdv)	Qual
	51	**Social cohesion scale**<br>Perceived better social cohesion in Off+ (or OnAdv)	Community survey
Social capital - bridging	52	**Bridging capital**<br>Bonding capital is more evidence in Off+ (or OnAdv)	Qual

Potential FCF		Factor/Theme/Hypothesis	Data type
**Service domain**			
Quantity	53	**Service availability**<br>There are more services available in Off+ than OnDis	Qual
	54	**Service availability**<br>Higher average density of services (count/LC km^2) in Off+ than OnDis	GIS
	55	**Service (location)**<br>Services are located near civil activity in Off+ more than OnDis LCs	Qual
	56	**Medical Services availability**<br>More medical services in Off+ than OnDis	Qual
	57	**Medical Services availability**<br>A higher proportion of perceived availability of Doctors/Medical Clinics in Off+ than OnDis	Community survey
	58	**ECEC Availability**<br>There are more ECEC services located in Off+ than OnDis	Qual
	59	**ECEC Availability**<br>Higher proportion of perceived Childcare/occasional care services in my suburb or local area in Off+ than OnDis	Community survey
	60	**ECEC Availability**<br>A higher proportion of preschools or kindergartens in my suburb or local area in Off+ than OnDis	Community survey
	61	**ECEC Availability**<br>Higher average density of ECEC services (count/LC km2) in Off+ (or OnAdv)	GIS
	62	**Primary School Availability**<br>There are more Primary Schools located in Off+ than OnDis	Qual
	63	**Primary School Availability**<br>Higher average density of Primary schools (count/LC km2) in Off+ (or OnAdv)	GIS
	64	**Whole-of-community service Availability**<br>Whole-of-community services are located in Off+ than OnDis	Qual

Potential FCF		Factor/Theme/Hypothesis	Data type
Access	65	**Service Utilisation**<br>Engagement with services is greater in Off+ than OnDis	Qual
	66	**Service Utilisation**<br>A higher proportion perceived use of services within Off+ (or OnAdv)	Community survey
	67	**ECEC Utilisation**<br>Engagement with ECEC services is greater in Off+ than OnDis	Qual
	68	**ECEC Utilisation**<br>A higher proportion perceived use of local preschool and kindergarten within Off+ (or OnAdv)	Community survey
	69	**ECEC Utilisation**<br>A higher proportion perceived use of local childcare and occasional care services in Off+ (or OnAdv)	Community survey
	70	**Primary School Utilisation**<br>Engagement with Primary Schools is greater in Off+ than OnDis	Qual
	71	**Primary School Utilisation**<br>A higher proportion of residents perceived use of Primary schools and kindergartens in Off+ (or OnAdv)	Community survey
	72	**ECEC Access**<br>Perceived access (cost, opening hours) to ECEC services is better in Off+ than OnDis	Qual
	73	**ECEC Access**<br>A higher proportion of ECEC services in Off+ (or OnAdv) are open to new families	Service Information
	74	**ECEC Access**<br>Average mean cost of ECEC services is less in Off+ (or OnAdv)	Service Information
	75	**Primary School Access**<br>Access (costs, opening hours) to primary school is perceived to be better in Off+ than OnDis	Qual
	76	**Primary School Access**<br>A higher proportion of residents perceived use of Primary schools and kindergartens in Off+ (or OnAdv)	Community survey
Quality	77	**Primary School Quality**<br>Quality of primary schools is perceived to be better in Off+ than OnDis	Qual
	78	**Primary School Quality**<br>A higher proportion of residents in Off+ perceive better quality primary schools and kindergartens than OnDis	Community survey
	79	**ECEC Quality**<br>Quality of ECEC is perceived to be better in Off+ than OnDis	Qual
	80	**ECEC Quality**<br>A higher proportion of ECEC services in Off+ have an ACECQA quality rating of exceeding or meeting recommended guidelines compared with OnDis	ACECQA
Coordination	81	**Service Coordination (co-location)**<br>Co-location of services is more evident in Off+ than OnDis	Qual
	82	**Service Coordination (integration)**<br>There is a stronger focus on service integration/coordination in Off+ than OnDis	Qual
	83	**ECEC Service Coordination (co-location)**<br>Co-location of services at ECEC sites is more evident in Off+ than OnDis	Qual
	84	**Primary School Service Coordination (co-location)**<br>Co-location of services at primary school sites is more evident in Off+ than OnDis	Qual

Potential FCF		Factor/Theme/Hypothesis	Data type
**Governance domain**			
Characteristics	85	**Historical factors**: Historical events that are associated with a stronger sense of citizenship and/or participation in Off+ (or OnAdv)	Qual
	86	**Priorities, policies and approaches**<br>Presence of local community groups/initiatives are more evident in Off+ (or OnAdv)	Qual
	87	**Multi-level governance**<br>More positive perceptions of networks and partnerships in Off+ (or OnAdv)	Qual
Coordination and Vision	88	**Leaders** and boundary spanners<br>Local champions or key leaders are more evident in Off+ (or OnAdv)	Qual
	89	**Shared vision**<br>There is a more clearly articulated vision for children in Off+ (or OnAdv)	Qual
	90	**Data and evidence**<br>Local data and consultation is more likely to guide decision making in Off+ (or OnAdv)	Qual
	91	**Resources, rules**<br>'novel approaches' or locally tailored initiatives or solutions have been developed in Off+ (or OnAdv)	Qual
Representation	92	**Community involvement**<br>A more positive perception of community involvement, and ability to have a say in Off+ (or OnAdv)	Qual
	93	**Community involvement**<br>There is greater engagement in community groups or activities in off diagonal Off+ (or OnAdv)	Community survey
	94	**Ability to have a say**<br>More likely to agree there are opportunities to have a real say on local issues in Off+ (or OnAdv)	Community survey
Cross-domain	95	**Locally based group**<br>A locally based group actively working on improving social cohesion and fostering social capital in Off+ (or OnAdv)	Qual
	96	**Partnership**<br>There is a stronger partnership between early years decision making and the universal service system in Off+ (or OnAdv)	Qual

7.2 Appendix 2. Measures used to explore the Differentiating FCFs

Community domain and sub-domain/s		Description ([1]quantitative; [2]qualitative)	Methodologies					
			Interview	Focus group	Community survey	GIS and park audit	Service information	Community demographics
1	Income*	Median household income[1]; Degree of economic diversity[2]	✓	✓	✓			✓
2	Highest level of schooling*	Percentage of population that have completed Year 12 or equivalent[1]			✓			✓
3	Gentrification	Higher income families are moving into the area resulting in displacement of more disadvantaged groups[2]	✓	✓				
4	Housing affordability	The price of housing is increasing in disadvantaged areas[2]	✓	✓	✓			✓
5	Housing tenure (stability)*	Proportion of renters compared to private owners[1]			✓			✓
6	Public housing*	Proportion of public renters[1]; Perceived presence of public housing[2]	✓	✓				✓
7	Housing density	Proportion of high rise (three or more storeys) vs Low rise[1]; Perceived density of dwellings[2]	✓	✓	✓	✓		✓
8	Stigma	Negative reputation of a local community[2]	✓	✓				
9	Historical events	Response of leaders to events that bring local community members together[2]	✓	✓				
10	Perceived service reputation (primary schools)	Perceptions of the service quality of local primary schools[2]	✓	✓	✓			
11	Perceived ECEC availability	Perceived availability of Early Childhood Education and Care in local community[2]	✓	✓	✓			
12	Perceived crime safety	Perceptions of crime in local community[2]	✓	✓	✓			
13	Local decision-making	As a result of local decision-making, 'novel approaches' or locally tailored initiatives or solutions (including any with a focus on social capital) have been developed in Off+[2]	✓	✓				

[1]Quantitatve; [2]Qualitative; *indicator

7.3 Appendix 3. Review of results with Triangulation

Physical Domain

Matched-disadvantaged community pair				VIC	NSW	NSW	NSW	QLD	QLD	ACT		
				1	2	3	4	5	6	7		
Sub-domain		Hypothesis for theme/factor	Data	Off-diagonal positive (Off+) vs. On-diagonal disadvantaged (OnDis) *within* each matched pair							Overall[a]	Triangulation[b]
**Walkability**												
Walkability	1	Perceived walkability to facilities is better in Off+	Qual	green	orange	red	green	orange	red	orange	Mixed	No
	2	Walkability Index Score of local community is higher in Off+	Quant1	orange	red	orange	orange	red	red	orange	Neutral	
Walkability	3	Perceived walkability to services is better in Off+	Qual	green	orange	red	orange	blue	red	blue	Mixed	No
	4	Walkability Index Score of local community is higher in Off+	Quant1	orange	red	orange	orange	red	red	orange	Neutral	
**Destinations and Facilities**												
Access to facilities and services	5	There are more perceived services available in Off+	Qual	green	red	red	green	orange	red	blue	Mixed	Yes
	6	More family destinations in Off+	Quant1	green	orange	red	orange	green	red	orange	Mixed	
Quality of facilities	7	Quality of facilities and places is better in Off+	Qual	green	orange	orange	green	green	orange	red	Mixed	No
	8	A higher proportion of residents perceive there are better quality services in Off+	Quant2	orange	orange	orange	orange	orange	orange	orange	Neutral	
**Public Open Space**												
Park availability and access	9	Perceived number of parks is higher in Off+	Qual	orange	orange	orange	orange	orange	orange	orange	Neutral	Yes
	10	More parks in Off+	Quant1	orange	orange	orange	orange	orange	orange	orange	Neutral	
	11	On average, parks in Off+ are closer (shortest distance (m) to park)	Quant1	orange	orange	green	orange	green	green	orange	Neutral	N/A

Matched-disadvantaged community pair				VIC	NSW	NSW	NSW	QLD	QLD	ACT		
				1	2	3	4	5	6	7		
Sub-domain		Hypothesis for theme/factor	Data	Off-diagonal positive (Off+) vs. On-diagonal disadvantaged (OnDis) *within* each matched pair							Overall[a]	Triangulation[b]
Park quality	12	Quality of POS and parks is perceived to be better in Off+	Qual	🟢	🟠	🟠	🟢	🟠	🟠	🟠	Neutral	Yes
Park quality	13	A higher proportion of residents perceive there are better quality local parks in Off+	Quant2	🟠	🟠	🟠	🟠	🟠	🟠	🟠	Neutral	
Park quality	14	Average public open space attractiveness score is greater in Off+	Quant1	🟠	🔴	🟠	🟢	🟠	🟠	🟠	Neutral	Yes
Park quality	15	Off+ has a higher Most attractive park score	Quant1	🟠	🔴	🟠	🟠	🟢	🔴	🟠	Neutral	Yes
**Public Transport**												
Public Transport	16	PT access and availability is better in Off+	Qual	🟠	🟠	🟠	🟠	🟠	🔴	🔴	Neutral	Yes
Public Transport	17	Distance (access) to PT is shorter in Off+	Quant1	🟠	🟠	🟠	🟠	🔴	🟠	🟠	Neutral	
Public Transport	18	A higher proportion of Off+ is within a PT stop (400m bus stop/600m tram/800m train or ferry)	Quant1	🟠	🟠	🟠	🔴	🔴	🟠	🟢	Neutral	Yes
**Housing**												
Public housing	19	Presence of public housing is less in Off+	Qual	🟠	🟢	🟢	🟢	🟢	🟢	🔴	Yes	Yes
Public housing	20	Proportion of public renters is less in Off+	Quant3	🟢	🟢	🟢	🟠	🟢	🟢	🟠	Yes	
Housing density	21	Housing type: Perceived higher housing density in OnDis than Off+ (this refers to number of storeys)	Qual	🟠	🟢	🟢	🟢	🟢	🟢	🔴	Yes	Yes
Housing density	22	There is a lower proportion of high density housing (3 or more storeys) in Off+	Quant1<br>Quant3	🟠	🟢	🟠	🟠	🟢	🟢	🟢	Yes	
Housing type	23	Public housing type: Compared with OnDis, there are more public housing classified as separate houses in Off+ compared with public housing classified as town houses/ apartments	Qual<br>Quant3	🟠	🟢	🟢	🟢	🟢	🟢	🔴	Yes	No
Housing type	24	Higher proportion of housing classified as separate houses in Off+ compared with OnDis (this is not necessarily public housing)*	Quant1<br>Quant3	🟠	🟠	🟠	🟠	🟢	🟢	🟢	Neutral	
Housing type	25	Higher proportion of housing classified as townhouses or apartments in OnDis than Off+ (this is not necessarily public housing)*	Quant1<br>Quant3	🟠	🟢	🟠	🟠	🟢	🟢	🟢	Yes	Yes

Matched-disadvantaged community pair				VIC 1	NSW 2	NSW 3	NSW 4	QLD 5	QLD 6	ACT 7		
Sub-domain		Hypothesis for theme/factor	Data	Off-diagonal positive (Off+) vs. On-diagonal disadvantaged (OnDis) *within* each matched pair							Overall[a]	Triangulation[b]
**Traffic exposure**												
Traffic	26	Traffic exposure: Lower perceived exposure to traffic in Off+	Qual	● (Neutral)	● (Neutral)	● (Neutral)	● (Neutral)	● (Mixed)	● (Yes)	● (Mixed)	Neutral	Yes
	27	Perceived lower traffic (TrafficSafety score) in Off+	Quant2	● (Neutral)	● (Neutral)	● (Neutral)	● (Neutral)	● (Neutral)	● (Neutral)	● (Neutral)	Neutral	
	28	Lower traffic exposure in Off+	Quant1	● (Neutral)	● (Neutral)	● (Neutral)	● (Neutral)	● (Neutral)	● (Neutral)	● (Yes)	Neutral	Yes

Off+: Off-diagonal positive; OnDis: On-diagonal disadvantaged

Qual: Qualitative data (interviews and focus groups); Quant1: GIS; Quant2: Community survey; Quant 3: ABS

[a]Summary finding in ≥4 community pairs; [b]Overall triangulation of qualitative and quantitative data (Yes=Match; N/A= Not sure/ only qual OR quant measure available; No=Not matched)

POS: Public Open Space; PT: Public Transport

- ● (green) Yes: Data supports theory
- ● (orange) Neutral: Data does not differentiate between local communities
- ● (red) No: Data does not support theory (supports opposite direction)
- ● (blue) Mixed: Not sure/not enough data

Social Domain

Matched-disadvantaged community pair				VIC 1	NSW 2	NSW 3	NSW 4	QLD 5	QLD 6	ACT 7		
Sub-domain		Hypothesis for theme/factor	Data	Off-diagonal positive (Off+) vs. On-diagonal disadvantaged (OnDis) *within* each matched pair							Overall[a]	Triangulation[b]
1	Stigma	**Perceived** Stigma is greater in OnDis	Qual	🟢	🟢	🟢	🟢	🟢	🔵	🔵	Yes	N/A
2	Sense of community	**Perceived sense of community** Sense of community (a different issue to stigma) is more evident in Off+	Qual	🟢	🟡	🔴	🟢	🔴	🟢	🔵	Mixed	No
		**Sense of community scale** There is perceived better sense of community in Off+	Quant1	🟡	🟡	🟡	🟡	🟡	🟡	🟡	Neutral	
3	Crime	**Perceived crime** Perceived risk of crime is greater in OnDis	Qual	🟢	🟢	🟢	🟢	🟢	🟡	🔵	Yes	No
		**Crime safety scale** Perceived crime safety is better in Off+	Quant1	🟡	🟡	🟡	🟡	🟡	🟡	🟡	Neutral	
		**Crime rates** Lower rates of crime against person in Off+	Quant2	🔴	🟢	🔵	🔴	🔵	🔵	🟢	Mixed	No
4	Social capital - bonding	**Perceived bonding social capital** Bonding capital is more evident in Off+	Qual	🟢	🟡	🟡	🟡	🔴	🟡	🔵	Neutral	Yes
		**Social cohesion scale** Perceived better social cohesion in Off+	Quant1	🟡	🟡	🟡	🟡	🟡	🟡	🟡	Neutral	
5	Social capital - bridging	**Bridging capital** Bonding capital is more evidence in Off+	Qual	🟢	🟡	🟡	🟢	🔵	🔵	🔵	Mixed	N/A

Off+: Off-diagonal positive; OnDis: On-diagonal disadvantaged

Qual: Qualitative data (interviews and focus groups); Quant1: Community survey; Quant2: Crime rates data

[a] Summary finding in ≥4 community pairs; [b] Overall triangulation of qualitative and quantitative data (Yes=Match; N/A= Not sure/ only qual OR quant measure available; No= Not Matched)

🟢 Yes: Data supports theory

🟡 Neutral: Data does not differentiate between local communities

🔴 No: Data does not support theory (supports opposite direction)

🔵 Mixed: Not sure/not enough data

SES Domain

Matched-disadvantaged community pair				VIC 1	NSW 2	NSW 3	NSW 4	QLD 5	QLD 6	ACT 7		
Sub-domain		Hypothesis for theme/factor	Data	Off-diagonal positive (Off+) vs. On-diagonal disadvantaged (OnDis) *within* each matched pair							Overall[a]	Triangulation[b]
1	Cultural diversity	**Language diversity** Proportion of adults aged 25-54 who speak a language other than English at home is greater in Off+	Quant1	🟠	🔴	🟠	🟠	🔴	🟠	🔴	Mixed	Yes
		**Cultural diversity** Level of cultural diversity is greater in Off+	Qual	🟠	🔴	🔴	🟠	🟠	🔴	🔴	Mixed	
		**People from similar backgrounds*** There is a higher proportion of people in Off+ who perceive others in the neighbourhood to be from similar backgrounds	Quant2	🟢	🟢	🟠	🟠	🟢	🟢	🟢	Yes	N/A
		**English Proficiency** Off+ has higher proportion speaking English only	Quant1	🟠	🟢	🟠	🟠	🟢	🟠	🟢	Neutral	No
		**Arrived in last 10 years** Proportion of residents arrived in last 10 years (2006-2016) is less in Off+	Quant1	🟠	🟠	🟠	🟠	🟢	🟠	🟢	Neutral	No
		**Aboriginal and Torres Strait Islander** Proportion of residents identifying as Aboriginal, Torres Strait Islander or both is less in Off+	Quant1	🟠	🟠	🟢	🟠	🟢	🟠	🟠	Neutral	No
2	Income	**Median weekly household income** Median household income is higher in Off+	Quant1	🟢	🟢	🟢	🔴	🟢	🟢	🟢	Yes	Yes
		**Higher income** Level of SES (economic diversity) is greater in Off+	Qual	🟢	🟢	🟢	🟢	🟢	🟢	🔴	Yes	
		**Household Income** Off+ has a higher proportion of residents aged 25-54 years with equivalised household weekly incomes greater than $2000/week	Quant1	🟠	🟠	🟠	🟠	🟠	🟠	🟠	Neutral	No
3	Age diversity	**Mix of younger and older people** Level of age diversity is greater in Off+	Qual	🟢	🔵	🟠	🟠	🟠	🟠	🔵	Neutral	Yes
		**Residents aged 65-84 years** Higher proportion of residents aged 65-84 years in Off+	Quant1	🟠	🟠	🟠	🟠	🟢	🔴	🟠	Neutral	

Matched-disadvantaged community pair				VIC 1	NSW 2	NSW 3	NSW 4	QLD 5	QLD 6	ACT 7		
Sub-domain		Hypothesis for theme/factor	Data	Off-diagonal positive (Off+) vs. On-diagonal disadvantaged (OnDis) *within* each matched pair							Overall[a]	Triangulation[b]
4	Housing affordability	**Housing affordability** Housing affordability is becoming more of an issue in the Off+ than OnDis: Higher SES families are moving into the area and displacing more disadvantaged groups	Qual	🟠	🟢	🟢	🟢	🔵	🟠	🟢	Yes	N/A
		**Housing affordability** Housing in Off+ is more desirable leading to less affordable housing in OnDis	Qual	🟠	🟢	🟢	🟢	🟢	🟠	🔴	Yes	
		**Housing affordability** In OnDis, A higher proportion of lower income households (lower 40%) are paying more than 30% of their income on housing costs	Quant1	🟠	🟠	🟠	🟠	🟢	🟠	🟢	Neutral	No with above
5	Housing Tenure	Lower proportion of renters in Off+ than on-diagonal	Quant1	🔴	🟢	🟢	🟢	🟢	🟢	🟢	Yes	N/A
6	Highest level of schooling	Off+ had higher levels of Year 12 than on-diagonal	Quant1	🟠	🟢	🟢	🟠	🟢	🟠	🟢	Yes	N/A
7	University degree (aged 25 to 54 years)	Off+ had higher proportion of residents aged 25-54 with a university degree	Quant1	🟠	🟢	🟠	🟠	🟠	🟠	🟠	Neutral	N/A
8	Working as managers or professionals (aged 25 to 54 years)	Off+ had high proportion of residents aged 25-54 years working as managers or professionals	Quant1	🟠	🟢	🟠	🟠	🟠	🟠	🟠	Neutral	N/A
9	Gentrification	Gentrification occurring more rapidly in Off+ compared to OnDis	Qual	🟢	🟢	🟢	🟢	🟢	🔵	🔵	Yes	N/A

Off+: Off-diagonal positive; OnDis: On-diagonal disadvantaged; *Needs to be explored further. Community survey was not reliable for use in KiCS

Qual: Qualitative data (interviews, focus groups); Quant1: ABS; Quant2: Community survey

[a]Summary finding in ≥4 community pairs; [b]Overall triangulation of qualitative and quantitative data (Yes=Match; N/A= Not sure/ only qual OR quant measure available; No=Not matched)

ECEC: Early Childhood Education Care; PS: Primary school

🟢 Yes: Data supports theory

🟠 Neutral: Data does not differentiate between local communities

🔴 No: Data does not support theory (supports opposite direction)

🔵 Mixed: Not sure/not enough data

Service Domain

Matched community pair				VIC 1	NSW 2	NSW 3	NSW 4	QLD 5	QLD 6	ACT 7		
	Sub-domain	Hypothesis for theme/ factor	Data	Off-diagonal positive (Off+) vs. On-diagonal disadvantaged (OnDis) *within* each matched pair							Overall[a]	Triangulation[b]
**Quantity**												
1	Service availability (location)	ALL: There are more services available in Off+ LCs than OnDis LCs (more services in OnAdv than Off-)	Qual	Green	Red	Red	Green	Green	Red	Blue	Mixed	No
1	Service availability (location)	HEALTH: A higher proportion of perceived availability of Doctors/ Medical Clinics in Off+ than OnDis	Quant2	Green	Orange	Orange	Orange	Orange	Green	Green	Neutral	No
1	Service availability (location)	ECEC: A higher proportion of perceived Childcare/ occasional care services in my suburb or local area*	Quant2	Green	Green	Green	Orange	Green	Red	Orange	Yes	No
1	Service availability (location)	ECEC: A higher proportion of perceived preschools or kindergartens in my suburb or local area*	Quant2	Green	Green	Orange	Orange	Green	Red	Red	Mixed	No
2	ECEC Availability	There are more perceived ECEC services located in Off+ LCs than OnDis LCs	Qual	Green	Green	Red	Green	Green	Red	Red	Yes	No
2	ECEC Availability	Higher average density of ECEC services (count/LC km2) in Off+ than OnDis	Quant1	Orange	Red	Red	Red	Green	Red	Red	No	No
2	ECEC Availability	ECEC: A higher proportion of perceived Childcare/ occasional care services in my suburb or local area*	Quant2	Green	Green	Green	Orange	Green	Red	Orange	Yes	Yes
2	ECEC Availability	ECEC: A higher proportion of perceived preschools or kindergartens in my suburb or local area*	Quant2	Green	Green	Orange	Orange	Green	Red	Red	Mixed	No
3	Primary School Availability	There are more Primary Schools located in Off+ LCs than OnDis LCs	Qual	Green	Blue	Orange	Green	Green	Red	Red	Mixed	No
3	Primary School Availability	Higher average density of Primary Schools (count/LC km^2) in Off+ than OnDis	Quant1	Orange	Orange	Orange	Orange	Orange	Orange	Red	Neutral	No
4	Whole-of-community service Availability	Whole-of-community services are located in Off+ more than OnDis LCs	Qual	Green	Orange	Orange	Orange	Orange	Red	Red	Neutral	N/A
4	Whole-of-community service Availability	No quantitative equivalent	Quant	-	-	-	-	-	-	-	N/A	N/A

Matched community pair				VIC 1	NSW 2	NSW 3	NSW 4	QLD 5	QLD 6	ACT 7		
Sub-domain		Hypothesis for theme/ factor	Data	Off-diagonal positive (Off+) vs. On-diagonal disadvantaged (OnDis) *within* each matched pair							Overall[a]	Triangulation[b]
**Access**												
5	Service Utilisation	Families in Off+ LCs use a greater range of service types (health, education, social) more than families in OnDis	Qual	green	orange	orange	green	orange	orange	orange	Neutral	No
		Families living in Off+ use services in different areas more than families in OnDis	Qual	orange	green	green	blue	green	green	orange	Yes	
		A higher proportion perceived use of services within suburb in Off+ LCs than OnDis*	Quant2	green	orange	orange	orange	orange	red	orange	Neutral	Yes
6	ECEC Utilisation	ECEC Utilisation: Perceived engagement with Early Childhood Education and Care (ECEC) services is greater in Off+ LCs than OnDis LCs	Qual	green	orange	blue	orange	green	orange	blue	Mixed	No
		A higher proportion perceived use of local preschool and kindergartens in Off+ compared with OnDis*	Quant2	orange	orange	orange	orange	orange	orange	green	Neutral	
		A higher proportion perceived use of local childcare and occasional care services in Off+ compared with OnDis*	Quant2	orange	orange	green	orange	orange	orange	orange	Neutral	
7	ECEC Access (cost)	Cost of ECEC services is more affordable in Off+ LCs than OnDis LCs	Qual	orange	orange	orange	orange	orange	red	red	Neutral	Yes
		Average mean cost of ECEC services is less in Off+ LCs than OnDis LCs	Quant3	orange	orange	orange	orange	orange	orange	orange	Neutral	
8	ECEC Access (capacity/ workforce, opening hours)	Capacity (waiting lists, meeting demand and workforce) of ECEC services is better in Off+ than OnDis LCs	Qual	orange	orange	blue	orange	orange	red	orange	Neutral	N/A
		Opening hours of ECEC is greater in Off+ than OnDis LCs	Qual	blue	orange	blue	blue	orange	blue	blue	N/A	
		Average opening hours is greater in Off+ LCs than OnDis LCs	Quant3	orange	orange	orange	orange	orange	orange	orange	Neutral	

Matched community pair				VIC	NSW	NSW	NSW	QLD	QLD	ACT		
				1	2	3	4	5	6	7		
Sub-domain		Hypothesis for theme/ factor	Data	Off-diagonal positive (Off+) vs. On-diagonal disadvantaged (OnDis) *within* each matched pair							Overall[a]	Triangulation[b]
9	Primary School Utilisation	Primary School Utilisation: Engagement with Primary Schools is greater in Off+ LCs than OnDis LCs	Qual	🟠	🟠	🟠	🟠	🟢	🟠	🔵	Neutral	N/A
		A higher proportion perceived use of preschools and kindergartens in Off+ LCs than OnDis* (note not exactly primary schools)	Quant2	-	-	-	-	-	-	-	N/A	
10	Primary School Access (cost, capacity/ workforce)	Capacity (waiting lists, meeting demand and workforce) of primary schools is better in Off+ than OnDis LCs	Qual	🟠	🟠	🟠	🔴	🟠	🟠	🔵	Neutral	N/A
		Cost of primary school is perceived to be better in Off+ LC than OnDis LC	Qual	🟠	🟠	🟠	🟠	🟠	🔴	🔴	Neutral	
		Cost of primary schools in Off+ LCs are cheaper compared with OnDis Note: many are government primary schools	Quant3	-	-	-	-	-	-	-	N/A	
**Quality**												
11	Primary School Quality (perception and accreditation)	Quality of primary schools is perceived to be better in Off+ LCs than OnDis LCs	Qual	🟢	🟢	🟢	🟢	🟢	🔴	🟢	Yes	N/A
		Quality accreditation/ licensing of schools is better in OFF+ LC than OnDis LC	Quant3	-	-	-	-	-	-	-	N/A	
		A higher proportion of residents in Off+ LCs (or OnAdv) perceive better quality preschools and kindergartens than OnDis** (note not exactly primary schools)	Quant2	-	-	-	-	-	-	-	N/A	
12	ECEC Quality (perception and accreditation)	Perceived quality of ECEC services is better in Off+ LCs than OnDis LCs	Qual	🟢	🟢	🟠	🟠	🟠	🔴	🟢	Mixed	No
		Quality accreditation/ licensing of early childhood education and care services (ECEC) is better in OFF+ LCs than OnDis LCs	Quant3	🔵	🟠	🔵	🔵	🔵	🔵	🟠	N/A	
		A higher proportion of services in Off+ LCs (or OnAdv) have an ACECQA quality rating of exceeding or meeting recommended guidelines compared with OnDis LCs	Quant3	🟠	🔴	🟠	🟢	🟢	🔴	🟠	Mixed	

Matched community pair				VIC	NSW	NSW	NSW	QLD	QLD	ACT		
				1	2	3	4	5	6	7		
Sub-domain		Hypothesis for theme/ factor	Data	Off-diagonal positive (Off+) vs. On-diagonal disadvantaged (OnDis) *within* each matched pair							Overall[a]	Triangulation[b]
**Coordination**												
13	Coordination (linkage, collaboration)	There is a stronger focus on service coordination in Off+ than OnDis LCs.	Qual	🟠	🟠	🔴	🟢	🟢	🟠	🟠	Neutral	N/A
		No quantitative equivalent	Quant	-	-	-	-	-	-	-	N/A	
14	Service Coordination (co-location)	Co-location of services is more evident in Off+ LCs than OnDis LCs	Qual	🟠	🟠	🟠	🟠	🟢	🔴	🔴	Neutral	N/A
		No GIS measure at the moment to assess co-location	Quant1	-	-	-	-	-	-	-	N/A	
15	ECEC Coordination (co-location)	Co-location of services at ECEC sites is more evident in Off+ LCs than OnDis LCs.	Qual	🟠	🟠	🟠	🟠	🟠	🔴	🔵	Neutral	N/A
		No GIS measure at the moment to assess co-location	Quant1	-	-	-	-	-	-	-	N/A	
16	Primary School Coordination (co-location)	Co-location of services at primary school sites is more evident in Off+ LCs than OnDis LCs.	Qual	🟠	🟢	🔴	🟠	🟠	🔴	🔴	Mixed	N/A
		No quantitative equivalent	Quant	-	-	-	-	-	-	-	N/A	

Off+: Off-diagonal positive; OnDis: On-diagonal disadvantaged; *Needs to be explored further. Community survey was not reliable for use in KiCS

Qual: Qualitative data (interviews and focus groups); Quant1: GIS; Quant2: Community survey; Quant3: Service information

[a]Summary finding in ≥4 community pairs; [b]Overall triangulation of qualitative and quantitative data (Yes=Match; N/A= Not sure/ only qual OR quant measure available; No=Not matched)

ECEC: Early Childhood Education Care; PS: Primary school

- 🟢 Yes: Data supports theory
- 🟠 Neutral: Data does not differentiate between local communities
- 🔴 No: Data does not support theory (supports opposite direction)
- 🔵 Mixed: Not sure/not enough data

Governance Domain

Matched-disadvantaged community pair				VIC 1	NSW 2	NSW 3	NSW 4	QLD 5	QLD 6	ACT 7		
**Sub-domain**		**Hypothesis for theme/factor**	**Data**	**Off-diagonal positive (Off+) vs. On-diagonal disadvantaged (OnDis) *within* each matched pair**							**Overall[a]**	**Triangulation[b]**
Characteristics	1	**Historical events:** There are (historical) events that are associated with a stronger sense of citizenship and/or participation in Off+ (or there are events that are associated with a weaker sense of citizenship in OnDis)	Qual	🟢	🔵	🔴	🟢	🟢	🟢	🟠	Yes	N/A
Characteristics	2	**Priorities, policies and approaches:** Presence of local community groups/ initiatives are more evident in Off+ (or OnAdv)	Qual	🟢	🟠	🔴	🟢	🔴	🟢	🟠	Mixed	N/A
Characteristics	3	**Multi-level governance** More positive perceptions of multi-level governance and coordination in Off+	Qual	🟢	🟠	🟠	🟠	🟢	🔵	🔴	Mixed	N/A
Characteristics	4	Role of history of LCs are different in on- and off-diagonal	Qual	🟢	🟠	🔵	🔵	🔵	🟠	🔴	Mixed	N/A
Coordination and Vision	5	**Leadership:** Presence of local champions/key leaders are more evident in Off+	Qual	🟢	🟠	🔴	🟢	🔴	🔴	🟢	Mixed	N/A
Coordination and Vision	6	**Shared vision:** There is a more clearly articulated vision for children in Off+	Qual	🟠	🟠	🟠	🟢		🔴	🟠	Neutral	N/A
Coordination and Vision	7	**Data and evidence:** Local data and consultation is more likely to guide decision making in Off+	Qual	🟠	🟠	🔴	🟢	🟢	🟠	🟠	Neutral	N/A
Coordination and Vision	8	**Local decision-making:** As a result of local decision-making, 'novel approaches' or locally tailored initiatives or solutions have been developed in Off+	Qual	🟢	🟠	🔴	🟢	🟠	🟢	🟢	Yes	N/A
Representation	9	**Community involvement:** A more positive perception of community involvement, engagement and the ability to participate and have a say in Off+	Qual	🟢	🟠	🟠	🟢	🟠	🟠	🟠	Neutral	N/A
Representation	10	**Community involvement:** There is greater engagement in community groups or activities in off diagonal Off+	Quant	🟢	🟠	🟠	🟢	🟢	🔴	🟠	Neutral	Yes
Representation	11	**Ability to have a say:** More likely to agree there are opportunities to have a real say on local issues in Off+	Quant	🟢	🟢	🟢	🟠	🟠	🟠	🟠	Neutral	
Cross domain	12	**Partnerships:** A stronger partnership between early years and the formal education system in Off+	Qual	🟠	🔵	🟠	🟢	🟠	🔴	🟠	Neutral	N/A
Cross domain	13	**Locally based group** actively working on improving social cohesion and fostering social capital in Off+	Qual	🟢	🔴	🔴	🟢	🔴	🟢	🟢	Yes	No

Off+: Off-diagonal positive; OnAdv: On-diagonal advantaged

[a]Summary finding in ≥4 community pairs; [b]Overall triangulation of qualitative and quantitative data (Yes=Match; N/A= Not sure/ only qual OR quant measure available; No=Not matched)

Qual: Qualitative data (interviews, focus groups); Quant: Community survey

🟢 Yes: Data supports theory

🔴 No: Data does not support theory (supports opposite direction)

🟠 Neutral: Data does not differentiate between local communities

🔵 Mixed: Not sure/not enough data

PART 8 **References**

1. Atkinson R, Wulff M, Reynolds M, Spinney A. Gentrification and displacement: the household impacts of neighbourhood change. Melbourne: 2011.
2. Brown D. Good Practice Guidelines for Indicator Development and Reporting (OECD). Third World Forum on 'Statistics, Knowledge and Policy': Charting Progress, Building Visions, Improving Life; 27-30 October; Korea. New Zealand2009.
3. Goldfeld S, Villanueva K, Tanton R, Katz I, Brinkman S, Woolcock G, et al. Kids in Communities Study (KiCS) study protocol: a cross-sectional mixed-methods approach to measuring community-level factors influencing early child development in Australia. BMJ Open. 2017;7:e014047.
4. Brooks-Gunn J, Duncan G, Klebanov P, Sealand N. Do Neighborhoods Influence Child and Adolescent Development. American Journal of Sociology. 1993;9(2):353-95.
5. Leventhal T, Brooks-Gunn J. The neighborhoods they live in: the effects of neighborhood residence on child and adolescent outcomes. Psychological bulletin. 2000;126(2):309.
6. Kohen DE, Leventhal T, Dahinten VS, McIntosh CN. Neighborhood disadvantage: Pathways of effects for young children. Child development. 2008;79(1):156-69.
7. Irwin L. Early child development: A powerful equalizer. Final report for the World Health Organization's Commission on the social determinants of health. http://wwwwho int/social_determinants/resources/ecd_kn_report_07_2007 pdf. 2007.
8. Department of Social Services. Families and Children Services 2016 [cited 2017 16 October]. Available from: https://www.dss.gov.au/our-responsibilities/families-and-children/programs-services/family-support-program/family-and-children-s-services.
9. Children Of. Opportunities for Children date unknown [cited 2017 16 October]. Available from: https://opportunitychild.com.au.
10. Together L. Logan Together date unknown [cited 2017 16 October]. Available from: http://logantogether.org.au/logan-together/.
11. Goldfeld S, Woolcock G, Katz I, Tanton R, Brinkman S, O'Connor E, et al. Neighbourhood Effects Influencing Early Childhood Development: Conceptual Model and Trial Measurement Methodologies from the Kids in Communities Study. Social Indicators Research. 2015;120(1):197-212.
12. Advisory Committee on Official Statistics. Good practice guidelines for the development and reporting of indicators. Wellington: Statistics New Zealand 2009.
13. Davern M, Gunn L. Community Indicators Victoria Melbourne, Australia2006 [cited 2017 10 October]. Available from: http://www.communityindicators.net.au/files/docs/CIV%2BResource%2BGuide.pdf.
14. Tanton R, Dare M, Brinkman S, Corti B-G, Katz I, Woolcock G, et al. Identifying off-diagonal communities using the Australian Early Development Census results. Social Indicators Research. 2015:1-16.
15. Shonkoff J, Richter L, van der Gaag J, Bhutta Z. An Integrated Scientific Framework for Child Survival and Early Childhood Development. Pediatrics. 2012;129(2):e460-e72.
16. Brinkman SA, Gregory TA, Goldfeld S, Lynch JW, Hardy M. Data Resource Profile: The Australian Early Development Index (AEDI). International Journal of Epidemiology. 2014;43(4):1089-96.
17. Bronfenbrenner U. The Ecology of Human Development: Experiments by Nature and Design. Cambridge, Massachussets: Harvard University Press; 1979.
18. Sampson R, Morenoff J, Gannon-Rowley T. Assessing "Neighbourhood Effects": Social Processes and New Directions in Research. Annu Rev Sociol 2002;28:443-78.
19. Goldfeld S, Mathews T, Brinkman S, Woolcock G, Myers J, Kershaw P, et al. The Kids in Communities Study: measuring community level factors influencing children's development. Report for VicHealth. Melbourne: Murdoch Childrens Research Institute, 2010.
20. Australian Bureau of Statistics. The Australian Statistical Geography Standard (ASGS) 2013 [cited 2014 09 May]. Available from: http://www.abs.gov.au/websitedbs/D3310114.nsf/home/Australian+Statistical+Geography+Standard+(ASGS).
21. Bazeley P. Qualitative Data Analysis. London: SagePublications; 2013.
22. Atkinson R, Flint J. Accessing hidden and hard-to-reach populations: Snowball research strategies. Social research update. 2001;33(1):1-4.
23. TableBuilder [Internet]. 2017. Available from: http://www.abs.gov.au/websitedbs/censushome.nsf/home/tablebuilder.
24. Community Profiles [Internet]. 2017. Available from: http://www.abs.gov.au/websitedbs/censushome.nsf/home/communityprofiles?opendocument&navpos=230.
25. Bradbury B. Spatial inequality of Australian men's incomes, 1991 to 20112017.
26. ESRI (Environmental Systems Resource Institute). ArcGIS v10. Redlands, California2010.
27. Murdoch Children's Research Institute, Parenting Research Centre, The Royal Children's Hospital Melbourne AGDoSS. Raising Children's Network Melbourne, Australia2006-2017. Available from: http://raisingchildren.net.au/.
28. Australian Urban Research Infrastructure Network [Internet]. n.d. Available from: https://aurin.org.au/.
29. QSR International. NVivo qualitative data analysis Software Version 11. QSR International Pty Ltd; 2015.
30. Harry B, Sturges KM, Klingner JK. Mapping the process: An exemplar of process and challenge in grounded theory analysis. Educational researcher. 2005;34(2):3-13.
31. Morse J. Critical Analysis of Strategies for Determining Rigor in Qualitative Inquiry. Qualitative Health Research. 2015;25(9):1212-22.
32. StataCorp. Stata Statistical Software: Release 13. In: College Station TSL, editor. TX: StataCorp LP.: College Station,; 2013.
33. Fielding NG. Triangulation and mixed methods designs data integration with new research technologies. Journal of Mixed Methods Research. 2012;6(2):124-36.
34. Villanueva K, Badland H, Kvalsvig A, O'Connor M, Christian H, Woolcock G, et al. Can the neighborhood built environment make a difference in children's development? Building the research agenda to create evidence for place-based children's policy. Academic pediatrics. 2016;16(1):10-9.
35. Bradley R, Corwyn R. Socioeconomic Status and Child Development Annual review of psychology. 2002;53(1):371-99.
36. Baum A, Garofalo J, Yali A. Socioeconomic Status and Chronic Stress: Does Stress Account for SES Effects on Health? Annals of the New York Academy of Sciences. 1999;896(1):131-44.
37. Duncan GJ, Brooks-Gunn J, Klebanov PK. Economic deprivation and early childhood development. Child development. 1994;65(2):296-318.

38. Formoso DW, R; Atkins, M. Gentrification and Urban Children's Well-Being: Tipping the Scales from Problems to Promise. American Journal of Community Psychology. 2010(3-4).
39. Joseph M. Early resident experiences at a new mixed-income development in Chicago. Journal of Urban Affairs. 2008;30(3):229-57.
40. Minh A, Muhajarine N, Janus M, Brownell M, Guhn M. A review of neighborhood effects and early child development: How, where, and for whom, do neighborhoods matter? Health & Place. 2017;46:155-74.
41. Barrera A. The role of maternal schooling and its interaction with public health programs in child health production. Journal of Development Economics. 1990;32(1):69-91.
42. Carneiro P, Meghir C, Parey M. Maternal Education, Home Environments, and the Development of Children and Adolescents. Journal of the European Economic Association. 2013;11(suppl_1):123-60.
43. Bicego GT, Ties Boerma J. Maternal education and child survival: A comparative study of survey data from 17 countries. Social Science & Medicine. 1993;36(9):1207-27.
44. Boyle MH, Georgiades K, Racine Y, Mustard C. Neighborhood and Family Influences on Educational Attainment: Results from the Ontario Child Health Study Follow-Up 2001. Child development. 2007;78(1):168-89.
45. Brooks-Gunn J, Duncan G, Aber J, editors. Neighborhood poverty, Volume 1: Context and consequences for children. New York: Russell Sage Foundation; 1997.
46. Brooks-Gunn J, Duncan G, Aber J, editors. Neighborhood poverty, Volume 2: Policy implications in studying neighborhoods. . New York: Russell Sage Foundation; 1997.
47. Boyle MH, Racine Y, Georgiades K, Snelling D, Hong S, Omariba W, et al. The influence of economic development level, household wealth and maternal education on child health in the developing world. Social Science & Medicine. 2006;63(8):2242-54.
48. Wilson William J. When work disappears: The world of the new urban poor. Alfred Knopf. 1996.
49. Ainsworth JW. Why Does It Take a Village? The Mediation of Neighborhood Effects on Educational Achievement*. Social Forces. 2002;81(1):117-52.
50. Waanders C, Mendez JL, Downer JT. Parent characteristics, economic stress and neighborhood context as predictors of parent involvement in preschool children's education. Journal of School Psychology. 2007;45(6):619-36.
51. Australian Bureau of Statistics. Census of Population and Housing: Nature and Content, Australia, 2016 Australia2015 [cited 2017 23 October]. Available from: http://www.abs.gov.au/ausstats/abs@.nsf/Lookup/by%20Subject/2008.0~2016~Main%20Features~Highest%20year%20of%20schooling%20completed~114.
52. Galster GC. The mechanism (s) of neighbourhood effects: Theory, evidence, and policy implications. Neighbourhood effects research: New perspectives: Springer; 2012. p. 23-56.
53. Atkinson R, Wulff M. Gentrification and displacement: a review of approaches and findings in the literature. Melbourne: 2009.
54. Formoso D, Weber R, Atkins M. Gentrification and Urban Children's Well-Being: Tipping the Scales from Problems to Promise. American journal of community psychology. 2010;46(3-4):395-412.
55. Wynn J, Deener A. Gentrification? Bring it. The Conversation. 2017.
56. Freeman L, Braconi F. Gentrification and displacement New York City in the 1990s. J Am Plan Assoc. 2004;70(1):39-52.
57. Dockery A, Kendall G, Li J, Mahendran A, Ong R, Strazdins L. Housing and children's development and wellbeing: a scoping study. Melbourne: 2010.
58. Newman S, Holupka S. Housing Affordability And Children's Cognitive Achievement. Health Affairs. 2016;35(11):2092-9.
59. Bratt R. Housing and Family Well-Being. Housing Studies. 2002;17(1):13-26.
60. Yates J, Milligan V. Housing affordability: a 21st century problem. Melbourne: 2007 21 September. Report No.
61. Harkness J, Newman S. Housing affordability and children's well-being: Evidence from the national survey of America's families. Housing Policy Debate. 2005;16(2):223-55.
62. Conger R, Ge X, Elder G, Lorenz F, Simons R. Economic stress, coercive family process, and developmental problems of adolescents. Child development. 1994;65(2):541-61.
63. Robinson E, Adams R. Housing stress and the mental health and wellbeing of families. Australian Institue of Family Studies, 2008.
64. Cutrona C, Wallace G, Wesner K. Neighbourhood characteristics and depression. Current Directions in Psychological Science. 2006;15(4):188-92.
65. Haurin D, Parcel T, Haurin R. Does Homeownership Affect Child Outcomes? American Real Estate & Economics Association. 2002;30(4):635.
66. Boyle M. Home Ownership and the Emotional and Behavioral Problems of Children and Youth. Child Development. 2002;73(2):883-93.
67. Taylor M, Edwards B. Housing and children's wellbeing and development: Evidence from a national longitudinal study. Family Matters. 2012(91):47-61.
68. Wood D, Halfon N, Scarlata D, Newacheck P, Nessim S. Impact of family relocation on children's growth, development, school function, and behavior. The Journal of the American Medical Association. 1993;270(11).
69. Leventhal T, Newman S. Housing and child development. Children and Youth Services Review. 2010;32(9):1165-74.
70. Weatherburn D, Lind B, Ku S. "Hotbeds of crime?" crime and public housing in urban Sydney. Crime & delinquency. 1999;45(2):256-71.
71. Ruel E, Oakley D, Wilson GE, Maddox R. Is public housing the cause of poor health or a safety net for the unhealthy poor? Journal of Urban Health. 2010;87(5):827-38.
72. Kearns A, Hiscock R, Ellaway A, Macintyre S. 'Beyond Four Walls'. The Psycho-social Benefits of Home: Evidence from West Central Scotland. Housing studies. 2000;15(3):387-410.
73. Evans G, Wells NM, Moch A. Housing and Mental Health: A Review of the Evidence and a Methodological and Conceptual Critique. Journal of Social Issues. 2003;59(3):475-500.
74. Yancey WL. Architecture, interaction, and social control: The case of a large-scale public housing project. Environment and Behavior. 1971;3(1):3-21.
75. Ferguson K, Cassells R, MacAllister J, Evans G. The physical environment and child development: An international review. International Journal of Psychology. 2013;48(4):437-68.
76. Kearns A, Whitley E, Mason P, Bond L. 'Living the High Life'? Residential, Social and Psychosocial Outcomes for High-Rise Occupants in a Deprived Context. Housing Studies. 2012;27(1):97-126.
77. Miles R, Coutts C, Mohamadi A. Neighborhood Urban Form, Social Environment, and Depression. Journal of Urban Health: Bulletin of the New York Academic of Medicine. 2011;89(1):1-18.
78. Giles-Corti B, Ryan K, Foster S. Increasing density in Australia: maximising the health benefits and minimising the harm. Melbourne: 2012.
79. McCulloch A. Housing density as a predictor of neighbourhood satisfaction among families with young children in urban England. Popul Space Place. 2012;18(1):85-99.

80. Buys L, Miller E. Residential satisfaction in inner urban higher-density Brisbane, Australia: role of dwelling design, neighbourhood and neighbours. J Environ Plan Manag. 2012;55(3):319-38.
81. Molnar BE, Gortmaker SL, Bull FC, Buka SL. Unsafe to Play? Neighborhood Disorder and Lack of Safety Predict Reduced Physical Activity Among Urban Children and Adolescents. American journal of health promotion. 2004;18(5):378-86.
82. Carver A, Timperio A, Crawford D. Playing it safe: The influence of neighbourhood safety on children's physical activity—A review. Health & Place. 2008;14(2):217-27.
83. Evans G, Ferguson K. Built Environment and Mental Health. In: Editor-in-Chief: Jerome ON, editor. Encyclopedia of Environmental Health. Burlington: Elsevier; 2011. p. 446-9.
84. Whitzman C, Mizrachi D. Creating Child-Friendly High-Rise Environments: Beyond Wastelands and Glasshouses. Urban Policy and Research. 2012:1-17.
85. Villanueva K, Giles-Corti B, Bulsara M, Timperio A, McCormack G, Beesley B, et al. Where do Children Travel to and What Local Opportunities Are Available? The Relationship Between Neighborhood Destinations and Children's Independent Mobility. Environment and Behavior. 2013;45(6):679-705.
86. Foster S, Wood L, Francis J, Knuiman M, Villanueva K, Giles-Corti B. Suspicious minds: Can features of the local neighbourhoods ease parents' fears about stranger danger? Journal of Environmental Psychology. 2015;42:48-56.
87. Carroll P, Witten K, Kearns R. Housing intensification in Auckland, New Zealand: Implications for children and families. Housing Studies. 2011;26(03):353-67.
88. Coley RL, Sullivan WC, Kuo FE. Where does community grow? The social context created by nature in urban public housing. Environment and behavior. 1997;29(4):468-94.
89. Palmer C, Ziersch A, Arthurson K, Baum F. Challenging the stigma of public housing: preliminary findings from a qualitative study in South Australia. Urban Policy and Research. 2004;22(4):411-26.
90. McCormick NJ, Joseph ML, Chaskin RJ. The new stigma of relocated public housing residents: Challenges to social identity in mixed-income developments. City & community. 2012;11(3):285-308.
91. MacLeod WB, Urquiola M. Anti-lemons: school reputation and educational quality. National Bureau of Economic Research, 2009.
92. Skallerud K. School reputation and its relation to parents' satisfaction and loyalty. International Journal of Educational Management. 2011;25(7):671-86.
93. Mashburn AJ, Pianta RC, Hamre BK, Downer JT, Barbarin OA, Bryant D, et al. Measures of classroom quality in prekindergarten and children's development of academic, language, and social skills. Child development. 2008;79(3):732-49.
94. Aturupane H, Glewwe P, Wisniewski S. The impact of school quality, socioeconomic factors, and child health on students' academic performance: evidence from Sri Lankan primary schools. Education Economics. 2013;21(1):2-37.
95. Hamre BK, Pianta RC. Early teacher–child relationships and the trajectory of children's school outcomes through eighth grade. Child development. 2001;72(2):625-38.
96. Catalano RF, Oesterle S, Fleming CB, Hawkins JD. The importance of bonding to school for healthy development: Findings from the Social Development Research Group. Journal of School Health. 2004;74(7):252-61.
97. Barnett WS, Ackerman DJ. Costs, benefits, and long-term effects of early care and education programs: Recommendations and cautions for community developers. Community Development. 2006;37(2):86-100.
98. Elliott A. Early childhood education: Pathways to quality and equity for all children. 2006.
99. Christian K, Morrison FJ, Bryant FB. Predicting kindergarten academic skills: Interactions among child care, maternal education, and family literacy environments. Early Childhood Research Quarterly. 1998;13(3):501-21.
100. Brilli Y, Del Boca D, Pronzato CD. Does child care availability play a role in maternal employment and children's development? Evidence from Italy. Review of Economics of the Household. 2016;14(1):27-51.
101. Cloney D, Cleveland G, Hattie J, Tayler C. Variations in the availability and quality of early childhood education and care by socioeconomic status of neighborhoods. Early Education and Development. 2016;27(3):384-401.
102. Baxter J, Hand K. Access to early childhood education in Australia: Australian Institute of Family Studies Melbourne; 2013.
103. Breunig R, Gong X, Treasury A, Weiss A, Yamauchi C. Child care availability, quality and affordability: Are local problems related to maternal labour supply: The Treasury; 2010.
104. Borrell LN, Graham L, Joseph SP. Associations of neighborhood safety and neighborhood support with overweight and obesity in US Children and Adolescents. Ethnicity & disease. 2016;26(4):469.
105. Singh GK, Ghandour RM. Impact of neighborhood social conditions and household socioeconomic status on behavioral problems among US children. Maternal and child health journal. 2012;16(1):158-69.
106. Pebley A, Sastry N. Concentrated Poverty vs. Concentrated Affluence: Effects on Neighborhood Social Environments and Children's Outcomes. Paper presented at the Annual Meeting of the Population Association of America; Minneapolis, MN, 2003.
107. Leventhal T, Brooks-Gunn J. The neighborhoods they live in: the effects of neighborhood residence on child and adolescent outcomes. Psychol Bull. 2000;126(2):309-37.
108. Sampson RJ. Family management and child development: Insights from social disorganization theory. Advances in criminological theory. 1992;3:63-93.
109. Foster S, Villanueva K, Wood L, Christian H, Giles-Corti B. The impact of parents' fear of strangers and perceptions of informal social control on children's independent mobility. Health & place. 2014;26:60-8.
110. Baklin S. Victimization rates, safety and fear of crime. Soc Probs. 1978;26:343.
111. Doran BJ, Burgess MB. Putting fear of crime on the map: Investigating perceptions of crime using geographic information systems: Springer Science & Business Media; 2011.
112. Ainsworth JW. Why does it take a village? The mediation of neighborhood effects on educational achievement. Social Forces. 2002;81(1):117-52.
113. Drukker M, Kaplan C, Feron F, van Os J. Children's health-related quality of life, neighbourhood socio-economic deprivation and social capital. A contextual analysis. Social Science & Medicine. 2003;57(5):825-41.
114. Wood L, Frank L, Giles-Corti B. Sense of community and its relationship with walking and neighborhood design. Social Science & Medicine. 2010;70(9):1381-90.
115. Leyden K. Social Capital and the Built Environment: The Importance of Walkable Neighborhoods. American Journal of Public Health. 2003;93(9):1546-51.
116. Wood L, Giles-Corti B. Is there a place for social capital in the psychology of health and place? Journal of Environmental Psychology. 2008;28:154-63.
117. Sen A. Development as Freedom. New York: Anchor Books; 1999.

118. Cornwall A, Coelho VS. Spaces for change? The politics of participation in new democratic arenas. In: Cornwall A, Coelho VS, editors. Spaces for Change? The politics of citizen participation in new democratic arenas. London: Zed Books; 2007. p. 1 - 32.
119. Eversole R. Community Agency and Community Engagement: Re-theorising Participation in Governance. Journal of Public Policy. 2011;31(01):51-71.
120. Torfing J, Peters BG, Pierre J, Sørensen E. Interactive Governance: Advancing the Paradigm. Oxford, UK: Oxford University Press; 2012.
121. Barnes M, Newman J, Sullivan H. Power, participation and political renewal: case studies in public participation. UK: The Policy Press; 2007. 228 p.
122. Mansbridge JJ, Morris A. Oppositional consciousness: The subjective roots of social protest: University of Chicago Press; 2001.
123. Carey G, Crammond B. What works in joined-up government? An evidence synthesis. International Journal of Public Administration. 2015;38(13-14):1020-9.
124. Carey G, Buick F, Malbon E. The Unintended Consequences of Structural Change: When Formal and Informal Institutions Collide in Efforts to Address Wicked Problems. International Journal of Public Administration. 2017:1-12.
125. Brennan D. Federalism, childcare and multilevel governance in Australia. In: Haussman M, Sawer M, Vickers J, editors. Federalism, feminism and multi-level governance. Surrey: Ashgate Publishing; 2010. p. 37-50.
126. Barraket J, Archer V, Mason C. The effects of hybridity on local governance: The case of social enterprise. In: Landvogt K, Carey G, Barraket J, editors. Creating and implementing public policy: Cross-sectoral debates. Oxford: Taylor and Francis; 2016.
127. Wilks S, Lahausse J, Edwards B. Commonwealth Place-Based Service Delivery Initiatives: Key Learnings project, Research Report 32. Melbourne: Australian Institute of Family Studies; 2015.
128. Reddel T. Exploring the Institutional Dimensions of Local Governance and Community Strengthening: Linking Empirical and Theoretical Debates. Brisbane: 2004.
129. Cornwall A, Schattan V, Coelho P. 1 Spaces for Change? The Politics of Participation in New Democratic Arenas. 2007.
130. Hancock L, Mooney G, Neal S. Crisis social policy and the resilience of the concept of community. Critical Social Policy. 2012;32(3):343-64.
131. Halpern R. Rebuilding the Inner City: a history of neighbourhood initiatives to address poverty in the United States. New York: Columbia University Press; 1995.
132. Steans J, Tepe D. The global and gendered dimensions of citizenship, community and 'cohesion'. International Politics. 2012;49(2):195-214.
133. Purdue D. Neighbourhood governance: Leadership, trust and social capital. Urban Studies. 2001;38(12):2211-24.
134. Bradford NJ. Cities and communities that work: Innovative practices, enabling policies: Canadian Policy Research Networks Canada; 2003.
135. Williams P. Collaboration in public policy and practice: Perspectives on boundary spanners: Policy Press; 2012.
136. Lowndes V, Sullivan H. How low can you go? Rationales and challenges for neighbourhood governance. Public administration. 2008;86(1):53-74.
137. Wiseman J. Local heroes? Learning from recent community strengthening initiatives in Victoria. Australian Journal of Public Administration. 2006;65(2):95-107.
138. Larsen L, Harlan SL, Bolin B, Hackett EJ, Hope D, Kirby A, et al. Bonding and bridging: Understanding the relationship between social capital and civic action. Journal of Planning Education and Research. 2004;24(1):64-77.
139. Goldfeld S, Mathers M, Mathews T, Katz I, Kershaw P, Brinkman S, et al. Understanding the community level factors that influence children's developmental outcomes: A literature review undertaken for the Kids in Communities Study (KICS) collaboration. 2010.
140. Sallis JF, Floyd MF, Rodríguez DA, Saelens BE. Role of built environments in physical activity, obesity, and cardiovascular disease. Circulation. 2012;125(5):729-37.
141. Napier MA, Brown BB, Werner CM, Gallimore J. Walking to school: Community design and child and parent barriers. Journal of Environmental Psychology. 2011;31(1):45-51.
142. Page A, Cooper A, Griew P, Jago R. Independent mobility, perceptions of the built environment and children's participation in play, active travel and structured exercise and sport: the PEACH Project. International journal of behavioral nutrition and physical activity. 2010;7(1):17.
143. Tappe KA, Glanz K, Sallis JF, Zhou C, Saelens BE. Children's physical activity and parents' perception of the neighborhood environment: neighborhood impact on kids study. Int J Behav Nutr Phys Act. 2013;10(39):1479-5868.
144. Kaczynski A, Besenyi G, Stanis S, Koohsari M, Oestman K, Bergstrom R, et al. Are park proximity and park features related to park use and park-based physical activity among adults? Variations by multiple socio-demographic characteristics. International journal of behavioral nutrition and physical activity. 2014;11(1):146.
145. Rosier K, McDonald M. The relationship between transport and disadvantage in Australia. Australian Institute of Families Studies, 2011.
146. Giles-Corti B, Vernez-Moudon A, Reis R, Turrell G, Dannenberg AL, Badland H, et al. City planning and population health: a global challenge. The Lancet. 2016;388(10062):2912-24.
147. Giles-Corti B, Kelty S, Zubrick S, Villanueva K. Encouraging Walking for Transport and Physical Activity in Children and Adolescents How Important is the Built Environment? Sports Med. 2009;39(12):995-1009.
148. Rothman L, Buliung R, Macarthur C, To T, Howard A. Walking and child pedestrian injury: a systematic review of built environment correlates of safe walking. Injury Prevention. 2014;20(1):41-9.
149. Chiesura A. The role of urban parks for the sustainable city Landscape and Urban Planning 2004;68:129-38.
150. Ward J, Duncan J, Jarden A, Stewart T. The impact of children's exposure to greenspace on physical activity, cognitive development, emotional wellbeing, and ability to appraise risk. Health & Place. 2016;40:44-50.
151. Christian HE, Klinker CD, Villanueva K, Knuiman MW, Foster SA, Zubrick SR, et al. The Effect of the Social and Physical Environment on Children's Independent Mobility to Neighborhood Destinations. Journal of Physical Activity and Health. 2015;12(6 Suppl 1):S84-93.
152. Loukaitou-Sideris A, Sideris A. What Brings Children to the Park? Analysis and Measurement of the Variables Affecting Children's Use of Parks. J Am Plan Assoc. 2009;76(1):89-107.
153. Francis J, Giles-Corti B, Wood L, Knuiman M. Creating sense of community: The role of public space. Journal of Environmental Psychology. 2012;32:401-209.
154. Schipperijn J, Ekholm O, Stigsdotter U, Toftager M, Bentsen P, Kamper-Jørgensen F, et al. Factors influencing the use of green space: Results from a Danish national representative survey. Landscape and Urban Planning. 2010;95:130-7.

155. Komro K, Flay B, Biglan A. Creating Nurturing Environments: A Science-Based Framework for Promoting Child Health and Development Within High-Poverty Neighborhoods. Clinical Child and Family Psychology Review. 2011;14(2):111-34.

156. Bejleri I, Steiner RL, Provost RE, Fischman A, Arafat AA. Understanding and Mapping Elements of Urban Form That Affect Children's Ability to Walk and Bicycle to School. Transportation Research Record: Journal of the Transportation Research Board. 2009;2137(1):148-58.

157. Chomitz VR, Aske DB, McDonald J, Cabral H, Hacker KA. The Role of Recreational Spaces in Meeting Physical Activity Recommendations Among Middle School Students. Journal of Physical Activity & Health. 2011;8:S8-S16.

158. Australian Children's Education & Care Quality Authority. ACECQA Information sheets 2018 [cited 2018 6 February]. Available from: https://www.acecqa.gov.au/resources/information-sheets.

159. Australian Government Department of Education and Training. Universal Access to Early Childhood Education 2018 [cited 2018 6 February]. Available from: https://www.education.gov.au/universal-access-early-childhood-education.

160. Findlay T. Report to Communities - Democratic Community Governance: Early Childhood Development Regimes in Canada. June. unpublished. 2015.

161. Bradford N. Cities and Communities that Work: Innovative Practices, Enabling Policies. 2003.

162. Saegert S. Building civic capacity in urban neighbourhoods: an empirically grounded anatomy. Journal of Urban Affairs. 2006;28(3):275-94.

163. Griffin L. Where is power in governance? Why geography matters in the theory of governance. Political studies review. 2012;10(2):208-20.

164. Leutz WN. Five laws for integrating medical and social services: Lessons from the United States and the United Kingdom. The Milibank Quarterly. 1999;77(1):77-110.

165. Moore T, McDonald M, Carlon L, O'Rourke K. Early childhood development and the social determinants of health inequities. Health Promotion International. 2015;30(S2):ii102-ii15.

166. Leutz WN. Five laws for integrating medical and social services: lessons from the United States and the United Kingdom. The Milbank Quarterly. 1999;77(1):77-110.

167. Zwi K, Henry R. Children in Australian society. Medical Journal of Australia. 2005;183(3):154-60.

168. Edwards B, Bromfield L. Neighbourhood influences on young children's emotional and behavioural problems. Family Matters. 2010;84:7-19.

169. Fjørtoft I, Sageie J. The natural environment as a playground for children: Landcape description and analyses of a natural landscape. Landscape and Urban Planning. 2000;48:83-97.

170. Bagot K. The importance of green play spaces for children - aesthetic, athletic and academic. Journal of the Victorian Association for Environmental Education. 2005;28(3):11-5.

171. Ellen IG, Turner MA. Does neighborhood matter? Assessing recent evidence. Housing policy debate. 1997;8(4):833-66.

172. Hewko J, Smoyer-Tomic KE, Hodgson MJ. Measuring neighbourhood spatial accessibility to urban amenities: does aggregation error matter? Environment and Planning A. 2002;34(7):1185-206.

173. Kwan M-P. The uncertain geographic context problem. Annals of the Association of American Geographers. 2012;102(5):958-68.

174. Handy S. Critical assessment of the literature on the relationships among transportation, land use, and physical activity. Transportation Research Board and the Institute of Medicine Committee on Physical Activity, Health, Transportation, and Land Use Resource paper for TRB Special Report. 2005;282(1):1-81.

175. Learnihan V, Van Niel K, GILES-CORTI B, Knuiman M. Effect of scale on the links between walking and urban design. Geographical Research. 2011;49(2):183-91.

176. Talen E, Anselin L. Assessing spatial equity: an evaluation of measures of accessibility to public playgrounds. Environment and planning A. 1998;30(4):595-613.

177. Bronfenbrenner U. The bioecological model from a life course perspective: Reflections of a participant observer. 1995.
